# Functional Electrolyte Additives: A Pinch of Salt/Solvent to an Electrolyte for High Energy Density Lithium‐Ion and Lithium–Metal Batteries

**DOI:** 10.1002/smll.202504276

**Published:** 2025-07-09

**Authors:** Chenrayan Senthil, Amutha Subramani, Ram K. Gupta, Zdeněk Sofer

**Affiliations:** ^1^ National Institute for Materials Advancement Pittsburg State University Pittsburg KS 66762 USA; ^2^ Department of Inorganic Chemistry Faculty of Chemical Technology University of Chemistry and Technology Prague Technická 5 Prague 6 16628 Czech Republic; ^3^ Department of Chemistry Pittsburg State University Pittsburg KS 66762 USA

**Keywords:** electrolyte formulation, electrode/electrolyte interphase, functional additive, lithium metal battery, lithium‐ion battery

## Abstract

Active electrode material in‐conjunction with an electrolyte allows to expand the horizons of electrochemical properties and safety of Li‐ion/Li–metal batteries. The role of electrolyte is pivotal in determining the performance metrics of rechargeable Li‐based batteries. However, the unavoidable side‐reactions imposed on the electrode active material and electrolyte due to the thermodynamic and kinetic conditions in a battery limits its performances. Electrolytes being ubiquitous for both the electrodes, a rational formulation containing a functional additive holds a great promise to tune the interfacial properties of electroactive material and to control the parasitic side reactions. Functional electrolyte additives are target specific, which serve the purpose of mitigating critical concerns related to material's properties, electrochemical performance, and safety of batteries scattered under different identities/chemistries. This review exclusively focuses on the significant impact of functional electrolyte additives on advancing the electrochemical performance, cycle‐life, and safety of Li‐ion/Li–metal batteries. The classification of additives and their mechanistic role is detailed on the basis of target‐oriented actions involving film formation and its abnormal growth, capacity retention, overcharge/over‐discharge, high‐voltage, wide‐temperature operation, acid and water contaminant, gas evolution, flammability, and aluminum corrosion. A perspective on the electrolyte and additive engineering considerate with the battery performances is detailed.

## Graphical abstract

Electrolytes, often termed as the “blood” of battery play a decisive role in determining their performance metrics. This review thoroughly analyzes the role of functional electrolyte additives improving the performance and safety of Li‐ion/Li–metal batteries involving film formation, overcharge control, wide temperature and voltage operation, scavenging contaminants, limiting gas evolution, fire‐safety, and anti‐corrosion. The mechanistic action of additives is discussed, and guidance for additives engineering and its formulation in electrolytes consensus to the practical applications, is projected.

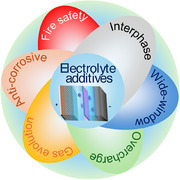

## 1 Introduction

Lithium(Li)‐ion batteries are a prodigious source of energy storage devices with applications spanning from portable technologies to smart transportation and grid storage.^[^ ^1^, ^2^, ^3^ ^]^ The widely spread modern utilities relying on Li‐ion batteries demand high energy density, which is quite beyond their current limitations and device engineering.^[^ ^4^, ^5^, ^6^ ^]^ Coupling lithium (Li) metal as an anode with a suitable high‐voltage cathode would open the space for high energy density batteries.^[^ ^7^, ^8^ ^]^ Conventional Li‐ion batteries integrate graphite as an anode, lithiated transition metal oxides/phosphates as a cathode, and a mono or multi‐layered polymer separator sandwiched between the electrodes; as a whole, they are wetted with an adequate amount of non‐aqueous electrolyte solution.^[^ ^9^, ^10^, ^11^, ^12^ ^]^ Under this configuration, the Li‐ions shuttle between the electrodes, i.e., Li‐ions released from the cathode migrate to the anode and occupy the graphite layers via an insertion mechanism. This process is electrochemically reversible and continues to cycle for over several hundreds of charge/discharge repetitions, anticipated to deliver high capacity and retention along with long cycle life. However, the formidable battery chemistries associated with the cathode, anode, and electrolyte solution do not remain as such that were available during the initial cycles.

From the materials perspective, graphite remains as an unmatched anode in the existing Li‐ion batteries due to its cycle life and low cost. However, limited theoretical capacity of graphite 372 mAh g^−1^ (LiC_6_) restricts its adoption as an anode for next‐generation high energy density batteries.^[^ ^13^, ^14^ ^]^ Lithium metal as an anode exemplifies a higher theoretical capacity 3860 mAh g^−1^, lowest electronegative potential −3.04 V versus SHE (standard hydrogen electrode), and very low density 0.534 g cm^−3^.^[^ ^15^, ^16^ ^]^ The promising attributes of Li metal enables it to be coupled with several cathodes such as LiCoO_2_ (≈140 mAh g^−1^), nickel‐rich layered oxides LiNi_1‐x‐y_Mn_x_Co_y_O_2_ (1‐x‐y > 0.5, > 200 mAh g^−1^), LiMn_2_O_4_ (≈120 mAh g^−1^), LiFePO_4_ (≈160 mAh g^−1^), lithium‐rich layered oxides Li_1+x_TM_1‐x_O_2_ (TM is a combination of Ni, Co, Mn, and Fe, >250 mAh g^−1^) and sulfur (1675 mAh g^−1^) paving the way for the development of next‐generation high energy density batteries.^[^ ^7^ ^−^ ^11^, ^17^, ^18^ ^]^ However, the inability of the electrode material and electrolyte refraining from the unavoidable side reactions promotes serious concerns limiting the performance and safety. For example, Li‐ion battery configured as graphite (or Li metal) ǀǀ LiMO_2_ (where M is transition metal(s)) suffer from issues like evolution of lattice oxygen when the voltage exceeds 4.3 V versus Li/Li^+^ (LiCoO_2_), metal dissolution in mixed metal layered cathodes, solvent co‐intercalation and exfoliation of graphite layers (propylene carbonate), Li plating, dendrites in Li metal anode, capacity fade, structural collapse, etc., which hampers the cyclability, capacity retention, cycle‐life, and poses a serious safety threat.^[^ ^19^, ^20^, ^21^, ^22^ ^]^ Several of such factors relevant to the operation of a battery in consensus with the active material, electrolyte, and supporting components contributing to the battery degradation were partially understood, while leaving a copious gap in expanding the knowledge on the material–performance–safety nexus.

Critical properties centric to the electrochemical performance safety which includes; active material‐electrolyte involving the formation of stable electrode/electrolyte interphase, i.e., solid‐electrolyte interphase (SEI), cathode‐electrolyte interphase (CEI), capacity retention, cycling stability, cycle life; safety properties like gas evolution, flammability, thermal instability, overcharge and high voltage control, and protection of passive component like corrosion of current collector must be promptly addressed to raise the standards of Li‐based batteries. Engineering the above properties relevant to material performance safety nexus through electrolyte formulations containing functional additives is regarded as a versatile solution. The rationally designed additive‐based electrolyte meticulously addresses the multi‐directional concerns and enables to reinforcement of high performance and the achievement of safer Li‐ion/Li–metal batteries. It is meanwhile worth noting that most of the electrolytes developed were centric to the graphite anode, thus, a rational electrolyte formulation garnered to wide variety of electrode chemistries is necessary. To be more specific on the need for and importance of specific electrolyte formulation and additives; ether‐based electrolytes show better interfacial stability with Li metal than the carbonate electrolytes, while the voltage window of carbonate electrolytes is comparatively higher. Formulating electrolyte composition desired for electrode chemistry requires screening and selection of appropriate electrolyte components which include salts, solvents, diluents, and additives. Scrutinizing the optimal electrolyte recipe chemistry could be experimentally carried out, however, it is a tedious and trial‐and‐error process. Machine learning (ML), a branch of artificial intelligence (AI) offers new opportunities to gather and accelerate knowledge in materials discovery and optimization.^[^ ^23^ ^]^ The exploitation of ML in realm of electrolyte chemistry offers predictive information on set of electrolyte components and formulations, ion‐solvation and interfacial chemistry, and desired functionality by analyzing extensive statistical and simulation‐derived datasets.^[^ ^24^ ^]^ Thus, a suitable electrolyte formulation and additive engineering are very much essential to achieve batteries of high‐energy density, capacity retention, extended cycle‐life, and safety.

**Figure 1:**
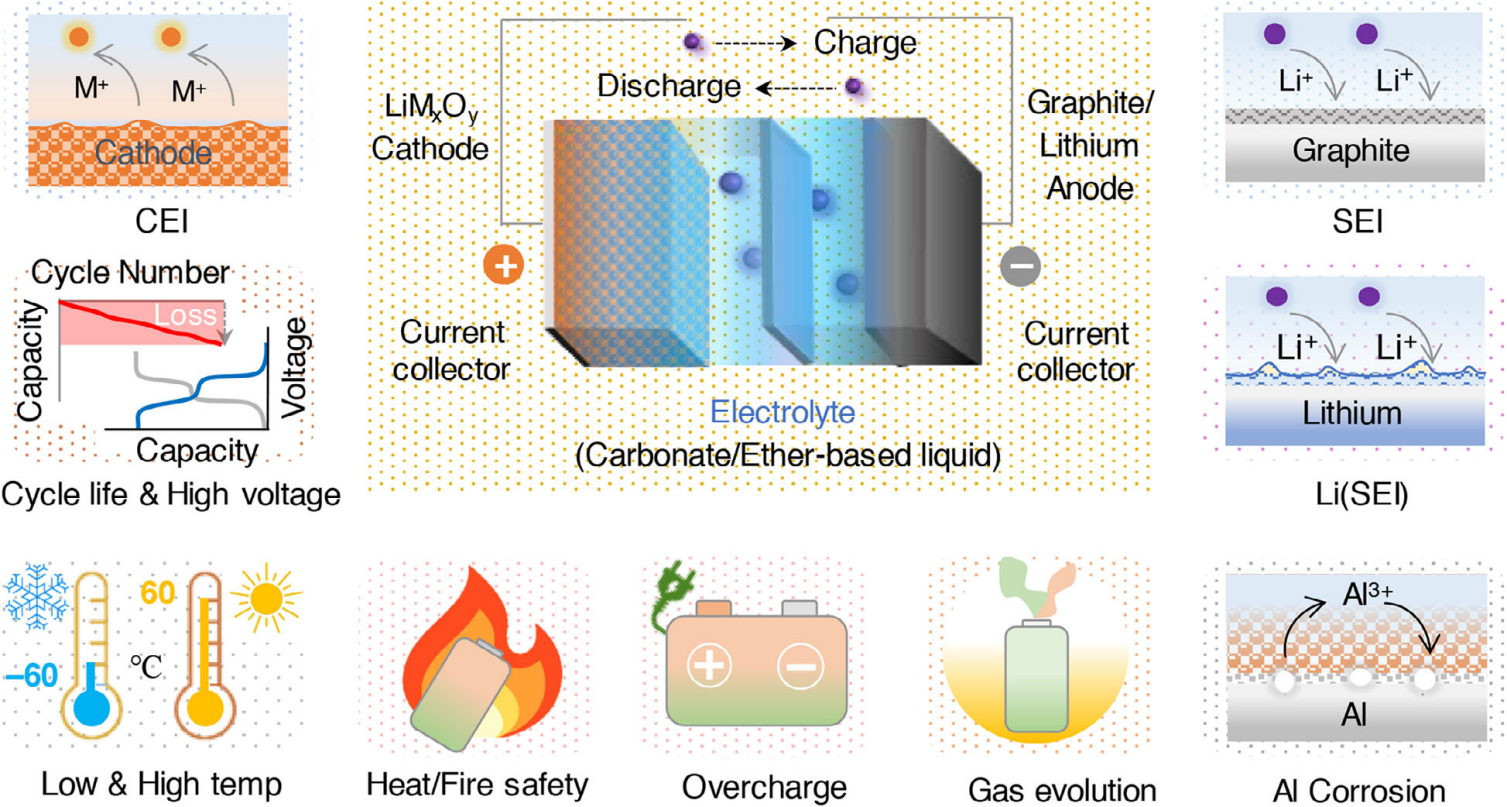
Schematic illustration of Li‐ion/Li–metal batteries and the respective interface and device‐engineering concerns related to their electrochemical performance, cycling stability, long‐cycle life, and safety.

The anode and cathode chemistries and safety of Li‐ion batteries, in a way, are dependent on the electrolyte solution and, importantly its formulation. This review exclusively deals with the rational optimization of non‐aqueous electrolytes, especially in the presence of functional additives. Engineering electrolyte formulation containing appropriate amount/concentration of additives in the form of salt and solvents for Li‐ion and Li–metal batteries were discussed. Since the role of functional additives is target‐oriented, the classification and discussion on the impact of electrolyte additives over the battery performance are based on the target specific actions. Performance metrics involving film formation, capacity retention, overcharge/over‐discharge protection, high‐voltage and wide‐temperature operation, acid and water scavenging, limiting gas evolution, flame retardant, and inhibiting aluminum corrosion are specifically deliberated as shown in **Figure** **1**. The role of elements and the chemical structures of functional additives are summarized to provide comprehensive understanding on their impact on Li‐based battery's performance. The underlying mechanism of the additive's role in promptly patching the critical concerns related to the material's properties, electrochemical performance, and safety of batteries is elaborated. Finally, guidance toward engineering additives and prospects on the development of functional additives and the electrolyte formulation toward accomplishing a high energy density, long cycle‐life, and safer batteries are projected.

## 2 Liquid Electrolyte: Components and Properties

Electrolytes, either liquid or solid‐based electrolytes in any rechargeable battery, serve as a medium to transport ionic charges between the active electrodes. In a liquid electrolyte, the solvated ions migrate either toward the anode or cathode and accommodate via any of the mechanism which includes (de‐)insertion/(de‐)intercalation, (re‐)conversion, and (de‐)alloy mechanisms. Meanwhile, the electrons are drawn through the external circuit to complete the electrochemical reaction. The electrolyte being a sole component in a battery system responsible for the ion transport, designing an electrolyte is crucial to achieve desirable cell performance. Especially, the redox properties of the electrodes in a way depend on the liquid electrolyte, which dictates the electrochemical performance such as capacity retention, long cycle life, rate capability, and safety of the rechargeable battery. The liquid electrolyte for a Li‐ion battery typically contains lithium salts, a mixture of organic solvents, and additives with desired concentrations/proportions. A rationally designed electrolyte is expected to possess a good solvation structure, ionic conductivity, ion transference number, and viscosity. The ionic conductivity for an electrolyte is calculated through Stokes law,

(1) $$\kappa =\sum_{i}\frac{{\left(z_{i}\right)}^{2}FC_{i}}{6\pi r_{i}}$$

where, Z*_i_* is charge number in charge transfer, *C*_i_ is molar concentration, F is Faraday constant, r*_i_* is radius of solvation ions. It is obvious that the physical properties of solute, concentration, and viscosity of electrolyte influence the ionic conductivity. Alongside, the dielectric constant of a solvent is an important parameter, where a solvent of higher dielectric constant allows to attain highly concentrated electrolytes.

### 2.1 Lithium Salts

Alkali salts, which are strictly based on the battery chemistries, are the source of ions that actively shuttle between the electrode through a non‐aqueous solvent medium. Initially, ions are solvated in a solvent environment and migrate toward the electrodes. At the juncture of electrode/electrolyte interface, the solvation sheath is shelled out making way for the ions to diffuse to an electrode. The ideal salts (Li salt for Li‐ion battery) are expected to possess properties such as their ability to dissolve and dissociate completely in a solvent, high mobility for the Li‐ions, and remain inert with the cell components. Since the cations are charge carriers in a liquid electrolyte, the stability of anions toward the electrochemical oxidation– and reduction–decomposition at the cathode and anode, respectively, must also be accounted for. Considering the above complexities with the anion chemistries, there are limited choice available for the Li‐based salts to be regarded as solutes in formulating liquid electrolytes for Li‐ion or Li–metal batteries. In addition, most of the Li salts fall short of minimum solubility requirement in a low dielectric medium due to the small ionic radius of Li‐ions. To counter the solubility issue, a complex Lewis‐base anion of large radius is preferred, where Li salts like lithium hexafluorophosphate (LiPF_6_), lithium perchlorate (LiClO_4_), lithium hexafluoroarsenate (LiAsF_6_), and lithium tetrafluoroborate (LiBF_4_) are employed to formulate carbonate‐based Li‐ion battery electrolytes. The well‐suited properties of LiPF_6_ such as high ionic conductivity, dissociation constant, and anodic stability enable it to be most preferred solute for the electrolytes in commercialized Li‐ion batteries. It should be noted that the carbonate‐based electrolyte formulations were specifically optimized for the graphite anode for Li‐ion batteries. Considering Li‐metal anodes, the most common Li‐salts are lithium bis(trifluoromethanesulfonyl)imide (LiTFSI, LiC_2_F_6_NO_4_S_2_) and lithium bis(fluorosulfonyl)imide (LiFSI, LiF_2_NO_4_S_2_) dissolved in a mixture of ether‐based solvents.

### 2.2 Solvents

Primarily, the solvent system in a liquid electrolytes dissolve and dissociate Li salts into mobile cations and anions. The commercial electrolyte formulation includes a mixture of non‐aqueous solvents in certain volume or weight proportion. It is well‐known that the ratio and choice of solvents significantly influence the physicochemical properties of a liquid electrolyte and play a critical role in the electrochemical properties such as solid electrolyte interphase (SEI) and cathode electrolyte interphase (CEI) formations.^[^ ^25^, ^26^, ^27^ ^]^ Basically, the non‐aqueous solvent for an electrolyte system perquisite a high dielectric constant (*ε*) to allow sufficient dissolution of salt, good ion transport, low viscosity (*η*), and chemical stability. In addition, the stability of a solvent constituting a liquid electrolyte is considered under two limits; operating voltage window and temperature limits are the fundamental parameters dictating the operational limits of Li‐ion batteries. To be precise, the electrolyte reduction/oxidation at the respective negative/positive potential governs the voltage limit, while the low‐ and high‐temperature and safety limits depend on the boiling temperature (*T*_b_), melting temperature (*T*_m_), and flash point (*T*_f_) of the solvent used. A summary of the most commonly used solvents and their properties in liquid electrolytes is listed in **Table** **1**. Cyclic carbonates such as ethylene carbonate (EC) and propylene carbonate (PC) are predominant choice of solvents, while the high dielectric permittivity and good SEI film forming ability of EC on graphite anode supersede the latter one as electrolyte solvent for Li‐ion batteries.^[^ ^28^ ^]^ However, to control the *T*_m_ of liquid electrolyte containing an EC, a mixture of one or more carbonate solvents such as acyclic carbonate or carboxylic esters are employed to formulate a liquid electrolyte. The common choices of acyclic carbonate or carboxylic esters are dimethyl carbonate (DMC), diethyl carbonate (DEC), and ethyl methyl carbonate (EMC) in certain proportions. It is important that the choice of salt and solvent constituting an electrolyte formulation certainly influences several properties, particularly film forming ability and cycling performances of batteries.

**Table 1:** Summary of properties of common Li salts for Li‐metal and Li‐ion batteries.

Li salts	Anionic radii [nm]	*T*_m_ [°C]	*T*_decomp._ [°C]	Ionic conductivity [mS cm^−1^]	Note
LiPF_6_	0.254	200	350	10.8	Thermally unstable
LiClO_4_	0.236	236	236	5.6	Explosive
LiBF_4_	0.227	296	100	4.9	Hydrolytic
LiAsF_6_	0.259	349	240	11.1	Toxic
LiFSI	0.264	124	200	12.3	Corrosive
LiTFSI	0.379	236	365	9	Corrosive
LiBOB	–	300	302	–	Low conductivity
LiDFOB	–	272	240	–	Resistive interface at low temperature

### 2.3 Additives

The selection of functional additives to fulfil a specific criterion in a battery demands properties based on the roles like film formation, high‐voltage, overcharge protection, fire retardant, etc. In general, the additives for liquid electrolytes are either solid particles or solvents being employed in a minimal amount/volume as compared to the primary electrolyte components solute and solvent. The maximum concentration of additives in an electrolyte could reach about 10% (mass or volume fraction) in comparison to electrolyte solvents, while a higher concentration is referred to as a part of base electrolyte.^[^ ^29^, ^30^ ^]^ To be more specific about the ratio, Solchenbach et al. proposed that a ratio of the additives to active material might be more relevant than considering a specific concentration, especially while comparing practical cells and lab‐scale coin cells.^[^ ^31^ ^]^ Generally, the electrolytes for commercial battery applications contain several functional additives, where the additive mixtures are expected to induce a synergistic effect rather than agonistic effect. Thus, ensuring the on‐demand role of additive mixtures and compatibility among the other battery components gains considerable importance while designing additive combinations. Burns et al. demonstrated that the additive mixture vinylene carbonate (VC) and fluoroethylene carbonate (FEC) improved the cycle‐life of graphite ǀǀ LiNi_0.33_Mn_0.33_Co_0.33_O_2_ Li‐ion cells than the cells that possessed one of the two additives.^[^ ^32^ ^]^ Mechanistically, the functional additive in an electrolyte operates in a several ways; alters the reactive decomposition of electrolyte solvent(s), getting decomposed prior to the solvent molecules, tuning the solvation shell, interaction with metal to control metal dissolution, generate scavengers via free radicals or compounds, etc., to fulfil the desired objective. As a result of an altered reaction mechanism, improved properties such as physical, chemical, mechanical, and electrochemical conditions relevant to the roles are observed, which certainly enhance the capacity and retention, lifetime, and safety of batteries.

**Figure 2:**
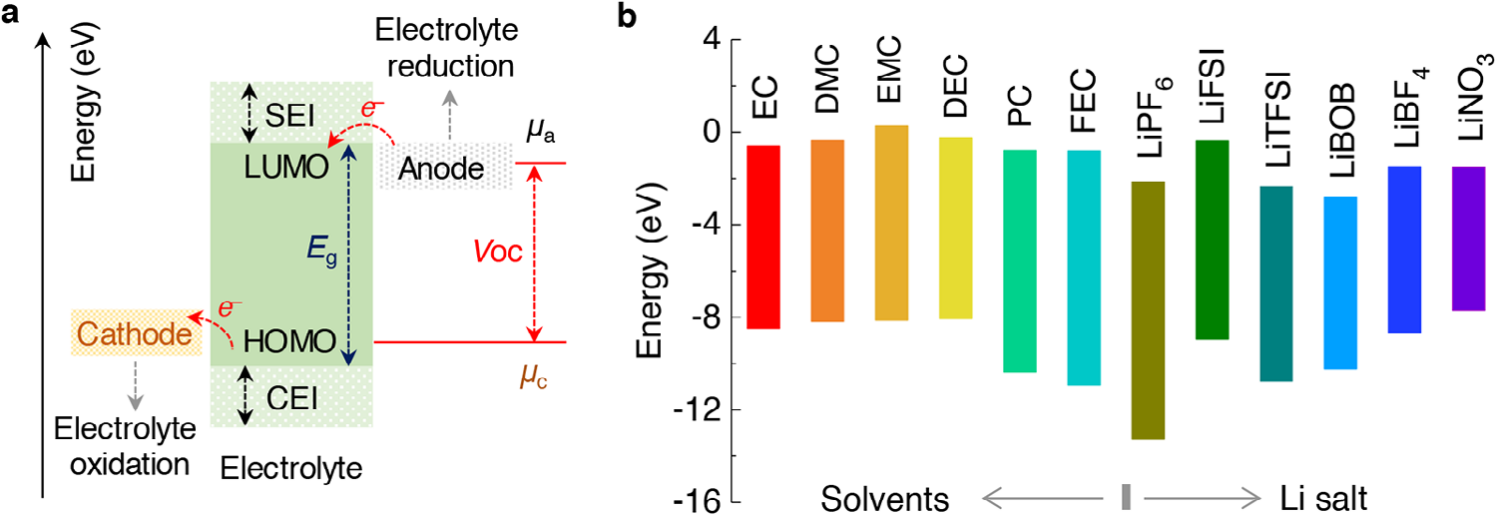
a) Energy diagram of HOMO and LUMO of electrolyte solution. b) Comparison of HOMO and LUMO energy levels for the common solvents and salts used in non‐aqueous Li‐based batteries.

The choice of functional additive to be introduced into an electrolyte depends on the energy levels, highest occupied molecular orbital (HOMO) and lowest unoccupied molecular orbital (LUMO) level that pertain to the cathodic and anodic reactions, respectively. Typically, the additive for SEI film formation is projected to possess a lower LUMO energy with higher reduction potential, i.e., the additive will be reduced first rather than the electrolyte decomposition on the anode surface. On the other hand, the additives must be stable to have a low HOMO energy with respect to the cathode material. Theoretical calculations suggest that the selection of organic additives to realize stable SEI films is expected to fit within the energy window −11–−9 eV in HOMO energy and −1–1 eV in LUMO energy.^[^ ^33^ ^]^ A summary of the HOMO and LUMO energies of few solvents and salts commonly used in Li‐based batteries are presented in **Figure** **2**. For example, the formation of SEI on the graphite anode and its stability over several cycles is not so effective in the presence of propylene carbonate (PC) solvent. It is likely understood that the PC molecules in an electrolyte along with the Li‐ions (Li(PC)_x_) co‐intercalates into the graphite causing a large degree of layer expansion. The residual stress due to solvent molecules and the expanded *c*‐spacing of graphite leads to a structural collapse perceived as exfoliation of graphene layers or crumbled graphite chunks.^[^ ^34^, ^35^ ^]^ Certainly, the addition of about 1 wt.% of any of the additives such as vinylene carbonate (VC), triphenyl phosphate (TPP), ethylene sulfate (DTD) or 10 wt.% of fluoroethylene carbonate (FEC) to EC:DEC electrolyte developed a stable SEI on graphite anode, offered stability toward electrolyte decomposition and mitigated the collapse of lithiated graphite.^[^ ^36^ ^]^ Thus, the rational knowledge on the energy levels and computability with other battery components is required to design efficient additives, which are effective in playing around the reductive/oxidative decomposition of electrolyte solvents.

### 2.4 Electrolytes for Li‐Ion/Li Metal Batteries

The electrolytes for Li‐ion/Li–metal batteries constitute Li salt, aprotic carbonate solvents and additives formulated in certain proportions. Among the components, the salt concentration and solvent ratio could be tuned, while maintaining a minimal dosage of additives to attain a balance among the ionic conductivity, stability, and safety of batteries. A standard electrolyte for Li‐ion batteries contains a blend of cyclic and linear carbonate solvents as 1 molar (M) salt solution, where salt LiPF_6_ is widely preferred due to its high ionic conductivity, dissociation constant, and anodic stability. It is widely noted that the maximum bulk electrolyte conductivity in most of the non‐aqueous electrolyte solutions occur at 1 M salt concentration, while an increase in the salt concentration impacts the conductivity, viscosity, solvation structure, coordination environment, and formation of interphases.^[^ ^37^, ^38^ ^]^ Commercial Li‐ion battery electrolytes often use salt concentration exceeding standard “1 M legacy” like 1.15 M or 1.2 M of LiPF_6_ containing EC and either one or mixture of solvents like DMC, DEC, and EMC in different volume proportions along with minimal dosage of additives. The adoption of diverse solvent mixtures in an electrolyte solution plays a critical role, the solvent EC possessing a high dielectric constant and viscosity principally dissociates the ions, while the co‐solvents DMC, DEC, and EMC of low viscosities and melting points afford a good balance of the physical and electrochemical properties at certain limitation imposed due to temperature and voltage range.^[^ ^39^, ^40^ ^]^ For Li–metal batteries, ether‐based solvents are a good choice due to good compatibility and stability with lithium metal anode. Electrolyte formulated as 1 M LiTFSI dissolved in 1,3‐dioxolane (DOL) and dimethoxyethane (DME) with *x*% LiNO_3_ (*x* = 0.5–3%, w/v) additives is the most common choice for Li–metal batteries, especially lithium–sulfur (Li–S) batteries. Typically, in the binary DOL:DME solvent mixture, cyclic ether DOL contributes to a stable and robust SEI on the Li metal, while the chain ether DME lowers the viscosity and improve the conductivity of electrolyte solutions.

Increasing the salt concentration in an electrolyte over 1.2 M leads to a new class of electrolytes termed as highly‐concentrated electrolytes (HCEs), the concentration exceeds 5 M or even 10 M (molality is more appropriate for HCEs). The HCEs encompass the advantage of increased electrochemical stability, improved ion transport properties, lower volatility, and high thermal stability at the expense of depleted solvation shell, electrolyte viscosity, and cost.^[^ ^41^ ^]^ Yamada et al. demonstrated reversible intercalation of Li into a graphite at HCEs > 3 mol dm^−3^ without any significant side reactions or solvent co‐intercalation.^[^ ^42^ ^]^ Further, binary LiTFSI‐EC HCEs (1:6 molar ratio) improved the Li plating/stripping Coulombic efficiency (CE) to about 97%.^[^ ^43^ ^]^ Likely, the adoption or transition toward HCEs requires extensive understanding on ion mobility, conductivity, and solvation structure. It must be noted that the mobility of ions is inversely proportional to the product of viscosity and ionic radius, denoting significantly reduced ion mobility while approaching higher salt concentration in electrolytes.^[^ ^44^ ^]^ To realize the benefit of highly concentrated electrolytes, solvents categorized as “diluents” are added to electrolyte solution forming a localized high‐concentration electrolyte (LHCEs), without compromising the ion transport and solvation structure. Ether‐based LCHEs demonstrated a cyclability over 100 cycles using LiFSI in TMS‐TTE and TMS‐TTE electrolytes.^[^ ^45^, ^46^ ^]^ Electrolytes HECs and LHCEs are appealing due to surplus supply of ions, while eradicating the ion depletion in electrolytes during long‐term cycling, yet high viscosity and poor wettability and performances at high‐rate must be considered.

**Figure 3:**
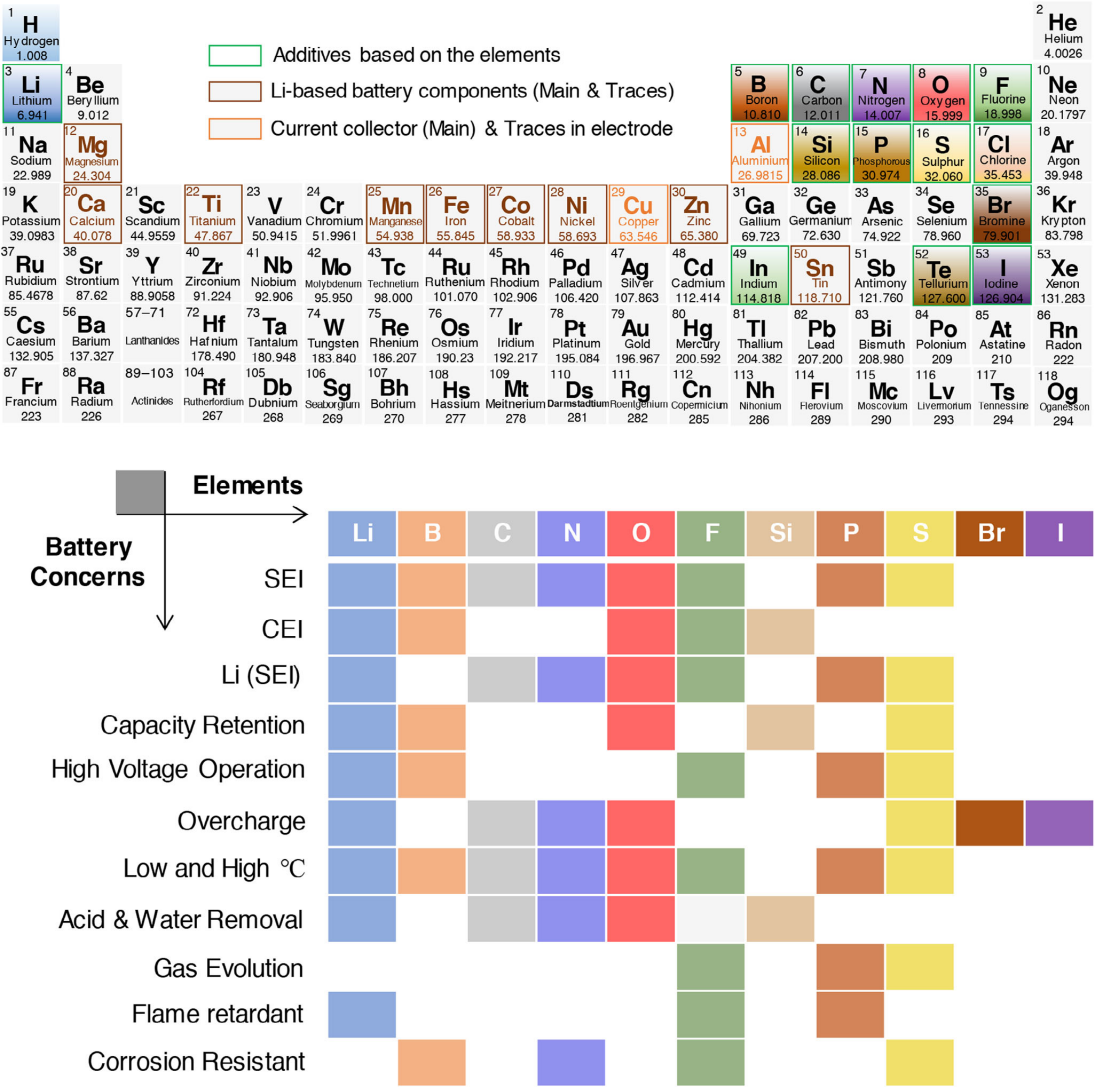
Periodic table of elements highlighting various roles of elements in battery chemistries (commercial), which include anode, cathode, electrolyte, additives, separator, and current collector. Summary of the elements constituting functional electrolyte additives and their desired roles in fulfilling Li‐ion/Li–metal battery concerns pertaining to improving the overall performances.

The electrochemically induced side reactions and the concerns that restrict the performance and compromise the safety of Li‐ion/Li–metal batteries include, weak solid electrolyte and cathode electrolyte interphases, Li metal passivation, capacity fading, instability at high voltage and at wide temperature, overcharging, acid and water contaminants, gas evolution, thermal risk and flammability, and corrosion of current collector. This review exclusively focuses on the above prime concerns and the developments achieved in the presence of additives‐based electrolytes. Broadly, the functional additives and their role in mitigating the performance and safety related properties of Li‐ion/Li–metal batteries are illustrated in Figure 1. Typically, the classification and specification of additives under a single roof is quite challenging. Alongside, the role of a single functional additive could not be expected to address or compensate for the collective critical concerns in Li‐based batteries. Bearing this, the discussion is laid on the functional additives that were adopted to fulfil specific battery issues that are categorized above. The elements that are part of the additive composition are organized based on the atomic number, and their role in combination with other common elements Li, C, N, and O are summarized in **Figure** **3**.

## 3 Computational and Experimental Methods

### 3.1 Computational‐Assisted Screening of Additives

Computational design and screening functional additive for an electrolyte solution rely on approaches such as first principles density functional theory (DFT), ab initio molecular dynamics (AIMD) calculation, and data‐driven machine‐learning. Density functional theory is used to compute properties like diffusion energy, band structures, and reaction energy, whereas information on chemical and structural properties of electrode–electrolyte interphase could be obtained from AIMD calculations.^[^ ^47^, ^48^ ^]^ To be adaptable as an additive for electrolytes, the energies of molecular orbitals must correlate to the decomposition potential, the HOMO energy relates to the oxidative decomposition potential and the LUMO energy corresponds to reductive decomposition. Specifically, the additive possessing higher HOMO energy than the solvents are expected to oxidize forming a cathode electrolyte interphase (CEI) layer, while the lower LUMO energy than the electrolyte solvent be reduced to modify the solid electrolyte interphase (SEI) layer.^[^ ^49^ ^]^ For example, the successful functional additives for CEI must possess lower oxidation potentials than the solvent molecules of electrolyte. Thus, the additives get oxidized prior to the electrolyte solvent molecule resulting in the formation of a CEI film.^[^ ^50^ ^]^ An alternative to DFT approach, Zhang et al. developed Gaussian process regression ML models based on vast datasets and predicted the redox potential of electrolyte additives.^[^ ^51^ ^]^ It is worth noting that selecting efficient variables to define appropriate criteria is critical for the estimation models to render reliable combinations.

Ab initio MD simulations provided information on the reaction of Li with electrolyte solvents and the electrode–electrolyte interface. The interaction of Li with the solvent molecules followed a stepwise two‐electron reduction of EC, where initial attack led to release of CO gas followed by an attack on other EC molecules contributing SEI products.^[^ ^52^, ^53^ ^]^ Further, details on Li‐ion transport, diffusion in the SEI, and ionic conductivity could be gathered. For instance, DFT tools shed light on the Li‐ion conductivity which is the sum of contributions from defects like interstitials, vacancies, Frenkel pairs, and Schottky pairs. It was revealed that the diffusion carrier in Li_2_CO_3_ varies with the open circuit voltage (OCV), where low OCV favored interstitial in Li metal, while Li^+^ vacancy at high OCV, say cathode surface. For LiF, the Li^+^ vacancies serve as dominating diffusion carriers; balanced either by F^−^ vacancies (Schottky pair) at low OCV or by electrons at high OCV.^[^ ^54^, ^55^ ^]^ The simulation studies decoded that an increase in the concentration of diffusion carriers promptly raises the Li‐ion conductivity.

The influence of ML has been profound in screening liquid electrolytes, redox potentials, solvation dynamics, and performance–safety metrics as a function of additives.^[^ ^56^, ^57^ ^]^ The ML‐driven data eventually arrive at optimal electrolyte design and formulation for proactive testing of novel electrolytes. Statistical data pertaining to the conduction of Li‐ions, decomposition of electrolyte, anode/cathode electrode interface, and correctional properties that influence the voltage range, temperature, and cycling stability of batteries could be gathered.^[^ ^47^ ^]^ Nakayama et al. estimated the coordination energy of solvent molecules with Li‐ion using an ML model.^[^ ^58^ ^]^ Further, prognostic information on the interfacial properties and ionic conductivity of electrolyte materials could be acquired from supervised ML models combined with DFT‐molecular dynamics (DFT‐MD) simulations.^[^ ^59^ ^]^ Kim et al. developed a novel electrolyte formulation containing fluorine‐free solvent methyl butyl ether, methyl *t*‐butyl ether, and dibutyl ether for Li metal anode through supervised ML model. A vast dataset related to a set of electrolytes, concentrations, and compositions that offered C.E of Li ǀǀ Cu cell between 80% and 99.5% were considered. Electrolytes using MTBE and MBE mixed with toluene demonstrated a high C.E of 99.7%.^[^ ^60^ ^]^ Since the ML discovery relies of datasets, the need for large and high‐quality datasets and inaccuracies with less trained models must be considered.

### 3.2 Experimental Methods

The oxidation potential of the electrolyte containing additives could be assessed using cyclic voltammetry. A configuration constituting Pt disk as a working electrode (WE), Li foil as a reference (RE), and counter electrode (CE) enables to determine the oxidation limit.^[^ ^61^ ^]^ Electrochemical impedance spectroscopy (EIS) provides details on the ionic conductivity of the electrolyte, resistive behavior at the electrode/electrolyte interphase, and the SEI formation. Owing to the complex nature of SEI, a direct observation through EIS alone is limited, where more advanced ex situ and in situ characterization techniques like X‐ray photoelectrochemical spectroscopy, cryo‐transmission electron microscopy, atomic force microscopy, nanoindentation, soft X‐ray absorption spectroscopy, etc. must be complemented. Several advanced characterization techniques would allow to further fetch in depth understanding on the underlying mechanisms. The evolution of Li dendrites was successfully observed through cryo‐TEM imaging which devoid any sensitivity due to environment and electron beam. Li et al. exposed the evolution of Li dendrite which occur at three growth directions, <111>, <110>, and <211> occupy about 49%, 32% and 19%, respectively.^[^ ^62^ ^]^ Time‐of‐flight secondary‐ion‐mass spectrometer (TOF‐MS) was adopted to understand the Li‐ion transport into the SEI layer. Lu et al. demonstrated the Li‐ion diffusion in a pre‐formed SEI using isotope ^7^Li modified ^7^LiClO_4_ salt and later was washed and soaked in an electrolyte containing ^6^LiBF_4_ salt. The TOF‐MS studies validated that the passage of BF_4_^−^ was up to ≈5 nm, while the ^6^Li^+^ penetrated deep over ≈20 nm thick SEI reaching the interphase of SEI ǀ current collector.^[^ ^63^ ^]^ In terms of electrochemical studies, galvanostatic charge/discharge tests enable to understand the capacity retention, cycle‐life, and CE of Li‐ion cells.

## 4 Functional Electrolyte Additives

### 4.1 Film Forming Additives

#### 4.1.1 Solid Electrolyte Interphase

**Figure 4:**
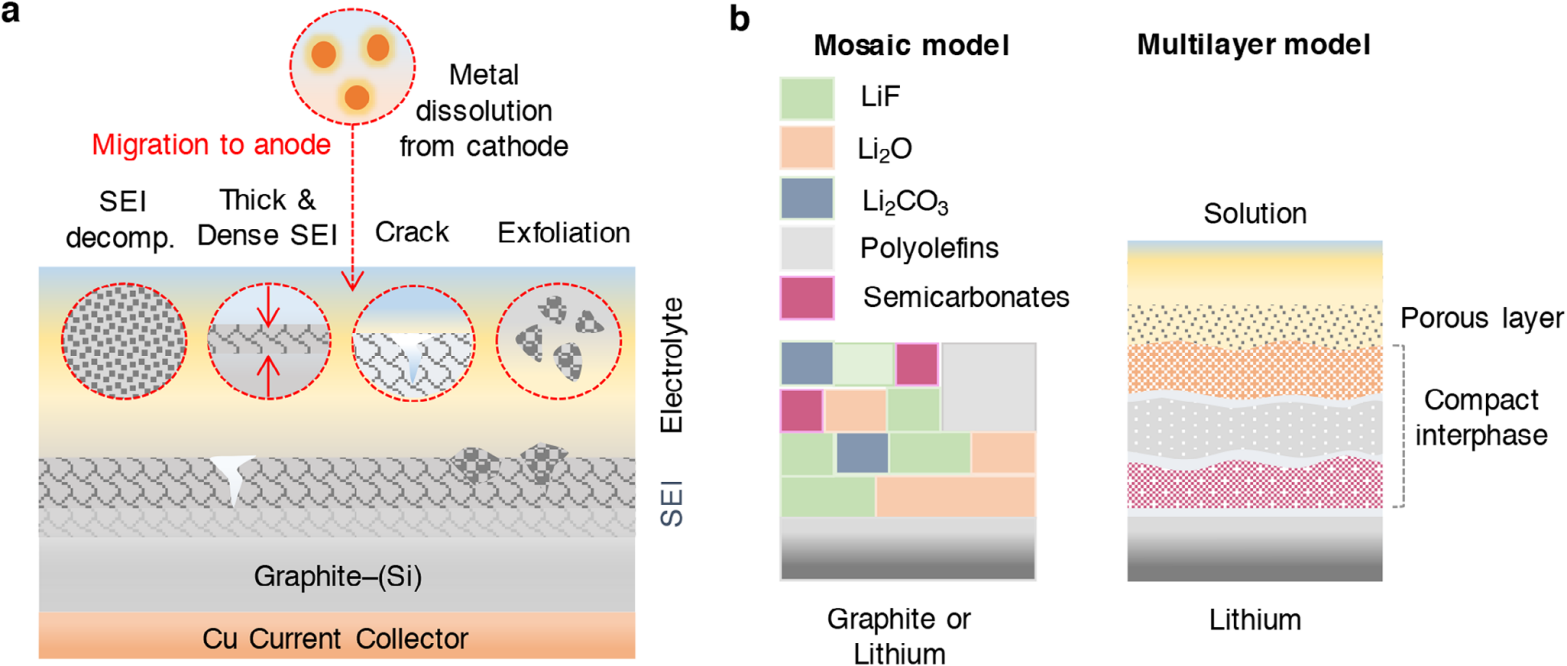
a) Schematics representing critical issues related to the solid‐electrolyte interphase in a graphite and graphite–silicon (–Si) anode material for Li‐ion batteries. b) SEI models: mosaic and multilayer models proposed by Peled and Aurbach, respectively.

A passivation layer/film termed as “solid electrolyte interphase” (SEI) develops on the surfaces of an anode material due to the electrolyte decomposition as portrayed in **Figure** **4**a.^[^ ^64^ ^]^ The formation of an SEI film is related to the redox potential of an electrode under condition that the potential lies outside the electrochemical window of the electrolyte solution as illustrated in Figure 2.^[^ ^65^, ^66^ ^]^ Typically, the SEI layer encompasses an organic and inorganic layer with several components such as Li_2_COLi, Li_2_CO_3_, and RCOOLi species. Besides, the inorganic compounds like LiF, LiCl, and Li_2_O settled on the electrode surfaces are the reduction products of salt anions as portrayed in Figure 4b. The chemical composition of SEI varies with the type of electrolyte formulation, where the role of salt and solvent employed is crucial for chemical (re‐)structuring of SEI layer. It is widely acknowledged that the formation and existence of such a protective layer over the electrode surface is essential for the operation of batteries in numerous ways, preventing further decomposition of electrolyte solvent, avoiding the exposure of fresh electrode surface to an electrolyte solution, and preventing the dissolution of an active material. Typically, the chemo‐mechanical functionality of SEI layer allows only the transport of Li‐ions while restricts the passage of electrons, i.e., electronically insulating, thereby preventing any further decomposition of electrolyte solvents. Alongside, the continuous growth of SEI film as a function of cycle number largely impacts the electrochemical performance of Li‐ion batteries, especially irreversible capacity, capacity loss, rate capability, cycle life, and safety due to consumption of electrolyte components.^[^ ^64^, ^67^, ^68^ ^]^ A more in‐depth knowledge on the SEI is needed; however, it is quite a challenging task due to the complex organic–inorganic structure of SEI and the capability of existing analytical tools. Alongside, the low and high temperature cycling of Li‐ion/Li–metal batteries adds more intricacy in expanding the knowledge of SEI films. Several factors are directly associated with the SEI growth and stability, which includes the film forming ability, mechanical strength, ion mobility, *T*_m_ of solvent, composition, ionic conductivity, etc. A collection of critical issues associated with the formation, growth, and retention of SEI on the graphite and graphite–silicon (G–Si) anodes are categorized as decomposition, layer thickness, microstructural cracks, exfoliation, and migration and diffusion of metal ions from cathode materials are illustrated in Figure 4a. Thus, the interfacial understanding of the structure and chemical composition of SEI layer on any anode materials like graphite, silicon, tin, lithium‐metal, etc. has gained paramount interest, which is seen as one among the potential ways to develop a stable high energy density Li‐ion and Li–metal batteries. An elaborate state‐of‐understanding on the SEI and its properties relevant to Li‐ion batteries is discussed elsewhere.^[^ ^66^, ^67^ ^]^

**Figure 5:**
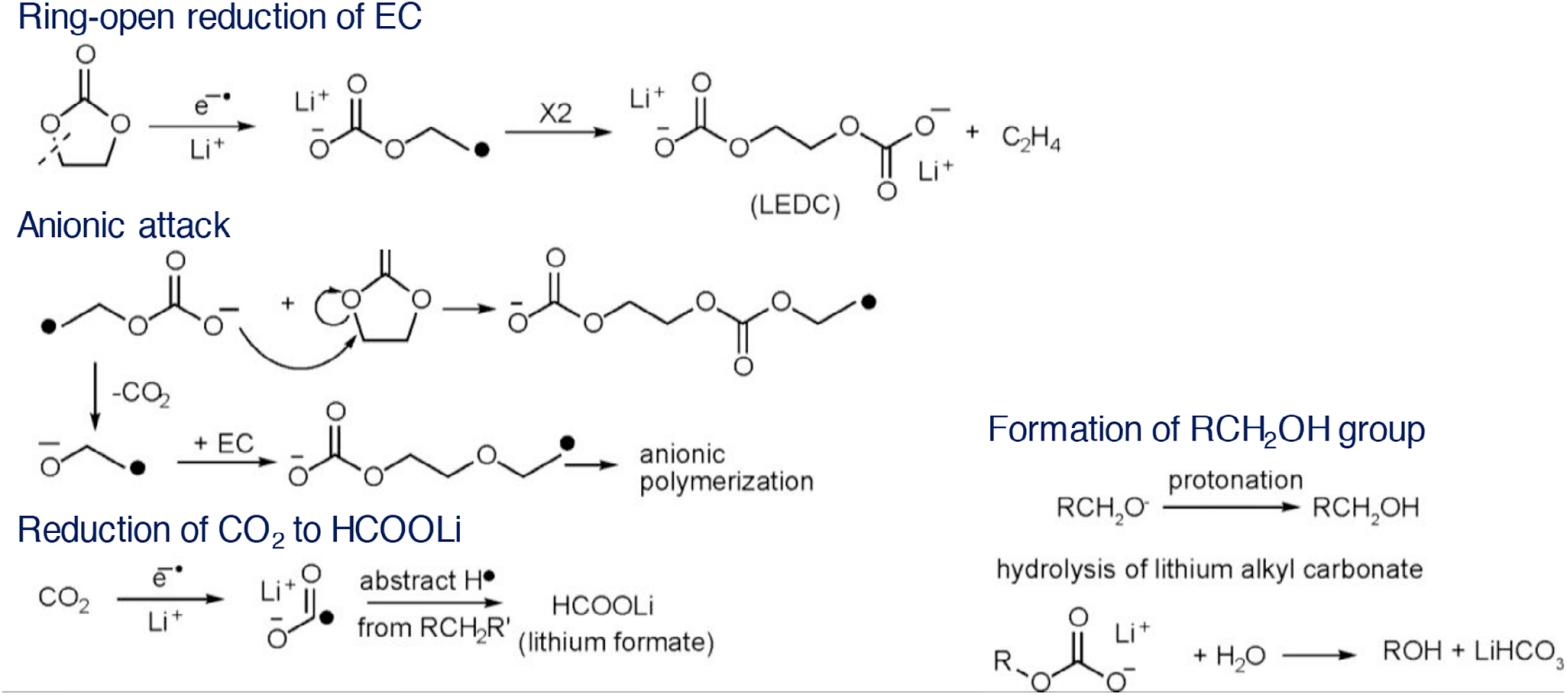
Schematics depicting the ring‐opening reaction of ethylene carbonate and the evolution of side‐products. Reproduced with permission.^[^ ^69^ ^]^ Copyright 2017, American Chemical Society.

In a commercial electrolyte for Li‐ion batteries, the widely used graphite anode and PC solvent does not realize a stable SEI which is capable of withstanding for a long‐term cycling. Typically, during the discharge of a graphite anode, the solvent molecules of propylene carbonate co‐intercalates with the Li‐ions into the graphite structure which certainly leads to an excessive expansion of graphite layers and subsequent exfoliation.^[^ ^36^ ^]^ Thus, it is important that the choice of salt and solvent in an electrolyte formulation be optimized to realize a stable film forming ability and thereafter improved cycling performances. Tracking the electrochemical decomposition pathway of solvents leaves a broad understanding on the role of additives. To illustrate, cyclic carbonates are prone to ring opening reaction during the reduction process, which is the most probable step involved in the formation of SEI film as portrayed in **Figure** **5**. Typically, the ring‐opening reduction of EC forms a lithium ethylene dicarbonate (LEDC) as a decomposition product followed by an evolution of ethylene gas. Further, the alkyl carbonate radical ion elongates the chain through nucleophilic attack on EC molecule. On the other hand, there are possibilities of losing CO_2_ to obtain ethylene oxide radical anion and subsequent formation of linear carbonates. Further, the lithium formate could be formed via the reduction of CO_2_, resulting in lithium carbon dioxide radical along with hydrogen abstraction from species in the electrolyte solution. The short‐chain alkyl carbonates like LEDC are soluble in the electrolyte medium and do not effectively protect the Si anode.^[^ ^69^ ^]^ In the case of PC as a solvent, the ring opening reaction occurs with the reduction of Li‐ions at the anode. The reduced Li‐ions readily interact with the oxygen that is bonded with carbon (C–O) to promote the opening of cyclic structure forming a linear carbonate molecule, which is a part of SEI component on the electrode's surface. Nevertheless, in the presence of cyclic carbonate, especially PC as a solvent for the graphite anode, the phenomenon of “solvent co‐intercalation” hampers the formation of a stable SEI which adversely limits the cyclic stability and electrochemical performances.^[^ ^70^, ^71^ ^]^

**Figure 6:**
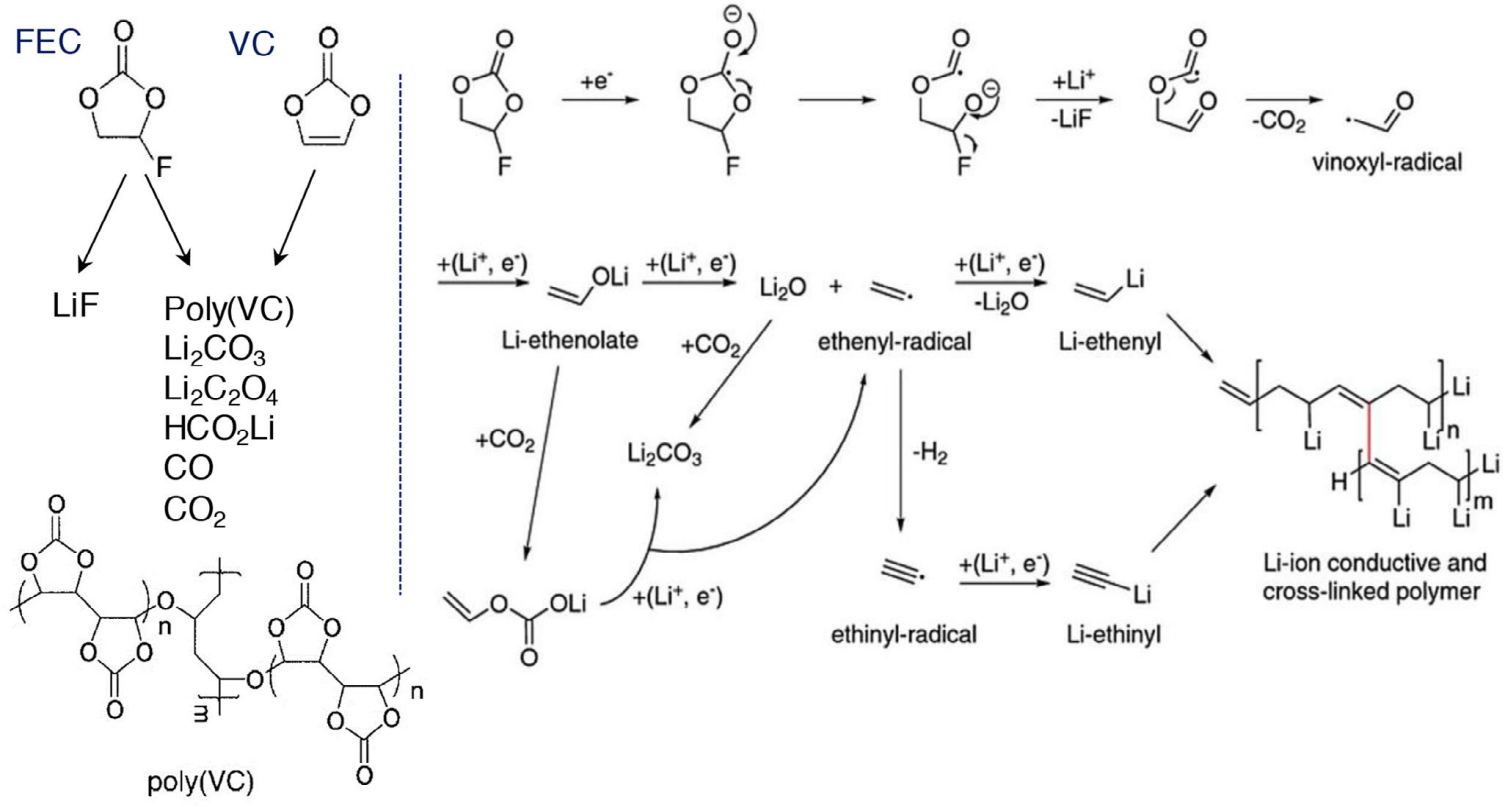
Schematic representation of the decomposition mechanism of vinylene carbonate and fluoroethylene carbonate to develop polymeric and side products. Reproduced with permission.^[^ ^91^ ^]^ Copyright 2016, American Chemical Society. Reproduced with permission.^[^ ^100^ ^]^ Copyright 2016 The Authors, The Electrochemical Society.

Hamamoto et al. found that the addition of sultone as an additive about 0.1–4 wt.% showed effectiveness in mitigating the PC co‐intercalation in graphite and carbon‐based anodes. Lithium‐ion cells configured as graphite ǀǀ LiCoO_2_ wetted using 1 M LiPF_6_–PC/DMC (1:1 wt.%) electrolyte containing 0.1 wt.% 1,3‐propane sultone (PS) showed cyclability for over 50 cycles with 82% capacity retention.^[^ ^72^ ^]^ It is likely that the reduction of 1,3‐propane sultone on graphite anode in the presence of carbonate electrolyte occur at a much higher potential than the carbonates forming a pre‐defined layer. Typically, the reduction of PS additive differed with various carbonate‐based electrolytes; 0.7 V versus Li/Li^+^ for 1 wt.% PS in 1 M LiPF_6_–PC/EC/EMC (1:1:3 wt.%) against a blank electrolyte at 0.5 V and 0.9 V for 2 wt.% PS in 1 M LiPF_6_–EC/EMC (1:2 wt.%) against 0.6 V for the blank electrolyte.^[^ ^73^, ^74^ ^]^ Li et al. employed PS as a bifunctional additive to simultaneously develop interfacial films on a graphite anode and LiMn_2_O_4_ cathode. The PS based electrolyte infiltrated graphite ǀǀ LiMn_2_O_4_ cells exhibited a capacity retention of 91% after 150 cycles at a 1 C rate under 50 °C, while the blank and VC additive‐based electrolytes showed lower capacity retention of 68% and 82%, respectively. It was noted that the SEI modified via PS additive was more resistant to an electrolyte decomposition and Mn deposition at a high temperature than the VC based electrolyte.^[^ ^75^ ^]^ Structural modification of the cyclic carbonates via introduction of either unsaturated bond or functional groups was found to be a prolific strategy to improve the film forming ability. Several of such modified carbonate structures including vinylene carbonate (VC),^[^ ^76^ ^]^ fluoroethylene carbonate (FEC), allyl methyl carbonate (AMC),^[^ ^33^ ^]^ allyl ethyl carbonate (AEC),^[^ ^77^ ^]^ 4,5‐dimethyl‐[1,3]dioxol‐2‐one (DMDO),^[^ ^78^ ^]^ methyl ethylene carbonate (MEC),^[^ ^79^, ^80^ ^]^ vinylethylene carbonate (VEC),^[^ ^81^ ^]^ and dimethyl 2‐oxo‐1,3‐dioxolane (ODC),^[^ ^82^ ^]^ were developed and reported as functional additives that effectively does the purpose of developing a stable SEI film either on graphite or Li metal anodes. From the mechanistic understanding on the structurally modified carbonates, the VC and FEC solvent molecules function as an additive through lowering the LUMO energy to facilitate the reduction of carbonate ring. Specifically, the lower reductive activation energy (13 kcal mol^−1^) and higher reduction potential (1.05–1.4 V versus Li/Li^+^) of VC than the EC (24.9 kcal mol^−1^ and 0.65–0.9 V versus Li/Li^+^) and PC (26.4 kcal mol^−1^ and 0.5–0.75 V versus Li/Li^+^) facilitated the solvent mixture to decompose much earlier than the native carbonate solvent.^[^ ^83^ ^]^ Further, the unsaturated C═C bond in VC molecule is disposed to polymerize as poly(VC) due to the prevalence of delocalized charges leading to a stable anion during the formation of SEI film.^[^ ^84^, ^85^ ^]^ In the case of FEC molecule, the strong electron withdrawing nature of fluorine reduces the LUMO level to undergo the decomposition of carbonates.^[^ ^86^, ^87^ ^]^ The reaction kinetics of FEC follows through the decomposition of intermediates VC and HF, where the poly(VC) forms a stable SEI film, while the HF combines with Li‐ions to convert as an inorganic LiF species. The decomposition reaction of FEC and VC molecules is illustrated in **Figure** **6**. It is well known that the fluorine rich SEI surfaces retain the uniform flux enabling stable Li‐ion deposition and its morphology.^[^ ^88^, ^89^ ^]^ A rational comparison of the SEI products formed through the VC and FEC additive revealed a higher degree of cross‐linked polymeric species for the VC, while inorganic LiF is enriched in the FEC additive‐based cells.^[^ ^90^ ^]^ Moreover, the inorganic species such as HCO_2_Li, Li_2_C_2_O_4_, and Li_2_CO_3_ constituting the SEI are common for both the VC and FEC additives, while the relative concentration of the species was higher for the VC due to differences in their reduction mechanisms.^[^ ^91^, ^92^ ^]^

**Figure 7:**
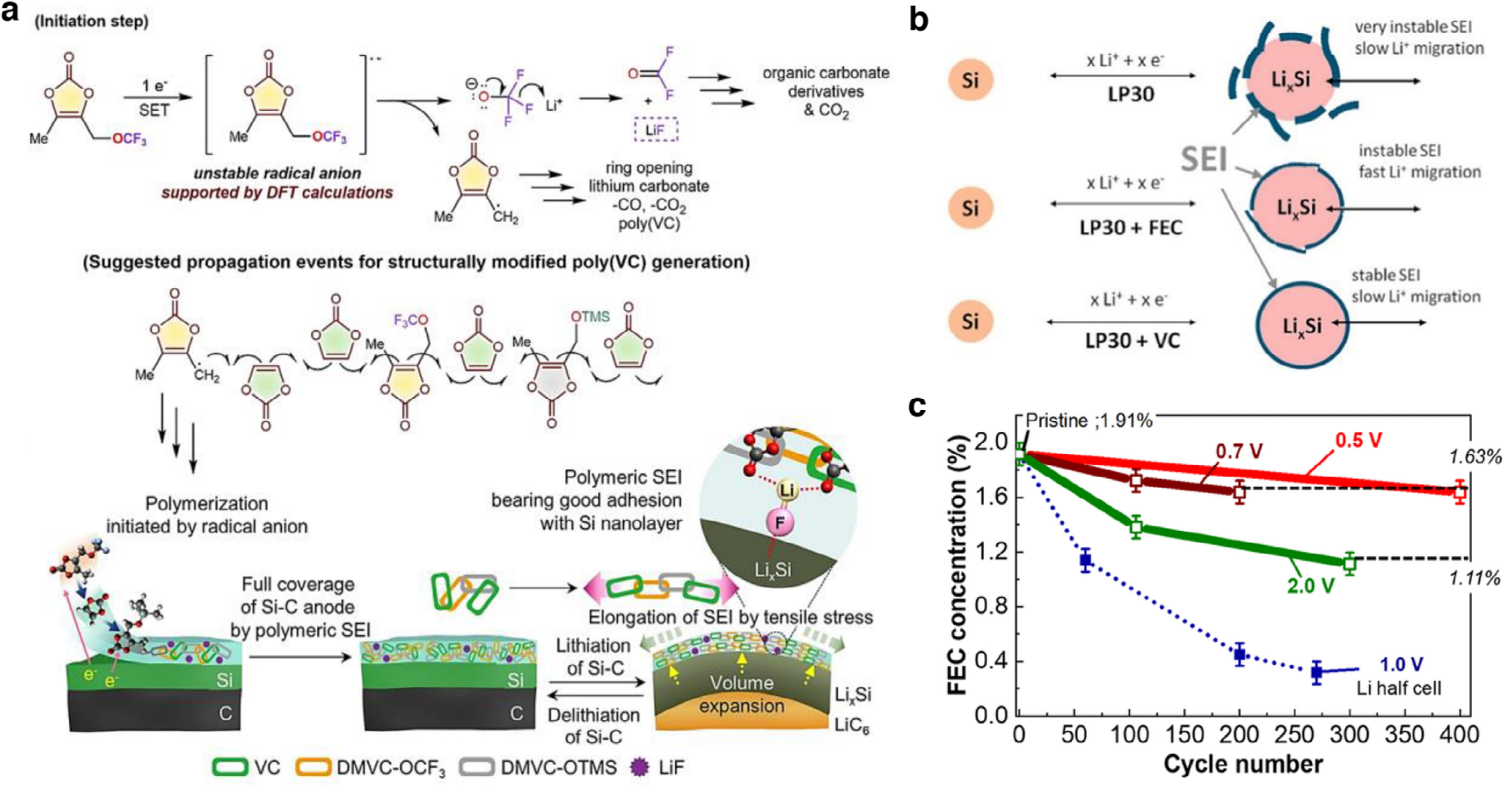
a) Mechanism depicting the transformation of DMVC derivative to construct SEI on the Si–G anodes. Reproduced with permission.^[^ ^101^ ^]^ Copyright 2021 The Authors, Springer Nature. b) Scheme depicting the influence of FEC and VC additive on the formation of SEI and electrochemical properties in the Si anodes. Reproduced with permission.^[^ ^102^ ^]^ Copyright 2017 The Authors, Elsevier. c) Cycling performance elucidating the consumption of FEC additive related to the cut‐off voltage in a G–Si ǀǀ LiFePO_4_ cells tested with LiPF_6_ in EC:DMC electrolyte. The quantification values are obtained through the gas chromatograph mass spectrometry (GS–MS) analysis. Reproduced with permission.^[^ ^108^ ^]^ Copyright 2023 The Authors, American Chemical Society.

The polymeric nature of VC, i.e., poly(VC) species formed during the reduction of electrolyte solvent is more compatible to anode materials being prone to severe volume changes during the electrochemical cycling. The Li‐ion insertion in the graphite anode (LiC_6_) with an ideal theoretical capacity of 372 mAh g^−1^ undergoes a volume expansion of about 13.2%, which is still by far reasonable among the most reported anode materials for Li‐ion batteries. Significant consequence of volume changes in graphite relates to losing the mechanical integrity of electrode through several micro‐macro cracks.^[^ ^93^ ^]^ However, high theoretical capacity anode materials such as silicon (Si, Li_4.4_Si is 4200 mAh g^−1^), tin (Sn, Li_4.4_Sn is 996 mAh g^−1^) are prone to large volume expansion/contraction (about 400% for Si and 300% for Sn) requiring the SEI film to be mechanically robust, elastic, and self‐repairable during the long‐term electrochemical cycling.^[^ ^94^ ^]^ Specific to Si anode, the existence of native oxide (SiO*_x_*) layer are vulnerable to the cyclability of Li‐ion batteries as they react with electrolyte forming low ion conductive Si phases like Li_4_SiO_4_ or Li_2_Si_2_O_5_.^[^ ^95^, ^96^ ^]^ Additives such as VC and FEC are used in combination or as co‐solvents with the electrolytes for Si and Sn anode materials. For the Si anode, the formation of SEI continues with the cycling as the low dense –Si–C and –Si–O groups are formed in a carbonate‐based electrolyte. Owing to a weak bonding of –Si–C and –Si–O groups, the decomposition of electrolyte continuous with the eventual SEI growth. Fluoroethylene carbonate as a co‐solvent even at a low concentration suppressed the continuous growth of SEI film as owed to strongly bonded –Si–F and LiF moieties.^[^ ^97^, ^98^ ^]^ Alongside, the construction of Poly(VC) molecules from the FEC upon electrochemical cycling provided a polymeric nature to the SEI, which remained robust over an extended cycle‐life.^[^ ^99^ ^]^ Further, the stability and capacity improvements of Si‐C anodes depend on the consumption or continuous decomposition of FEC, and such an enhancement in the performance could be accomplished as long as FEC is available in the electrolyte.^[^ ^100^ ^]^ Along with a VC additive, Park et al. introduced fluorinated and silylated additive targeting the volume expansion/contraction of Si‐embedded anodes. The combination of VC additive and the derivatives of VC bearing –OCF_3_ group and trimethylsilyloxy (–OTMS) moieties improved the G–Si ǀǀ NMC811 cell's capacities realizing 81.5% capacity retention after 400 cycles at 1 C rate. The developed 5‐methyl‐4‐(trifluromethoxy)methyl)‐1,3‐dioxol‐2‐one and 5‐methyl‐4‐((trimethylsilyloxy)methyl)‐1,3‐dioxol‐2‐one additives afforded spatial flexibility via polymeric propagation with the vinyl group of VC to the VC‐derived SEI (**Figure** **7**a). Mechanistically, the DMVC bearing –OCF_3_ group acted as radical precursor and the–OTMS moiety scavenged the HF in the electrolyte solution.^[^ ^101^ ^]^ Electrolyte formulation engineered to curtail certain issues related to SEI and performance related metrics are investigated by Jaumann et al. A comparative study on the FEC and VC as additives with commercial LiPF_6_ in EC:DMC electrolyte on the aspects of film forming ability and electrochemical performance in nano‐silicon anodes revealed diverse properties. Typically, VC additive showed an improved lifetime and efficiency at the expense of high resistance for Li‐ion migration. In contrast, the FEC showed higher Li‐ion conductivity with less reversibility and flexibility (Figure 7b).^[^ ^102^ ^]^ Based on the reaction pathway and by‐products of FEC (LiF, VC to Poly(VC)), Woods et al. introduced trimethylsilyl lithium (Me_3_SiLi), a silicon nucleophile as an additive to develop a robust polymeric SEI on the Si anodes. Tracking the reaction mechanism of Me_3_SiLi in LiPF_6_ with combinations of EC/EMC/VC/FEC electrolytes revealed that the FEC molecule reacted with Me_3_SiLi additive forming VC and poly(VC) via a base‐elimination reaction. The VC‐based by‐products are subsequently converted to insoluble and chemically stable poly(hydroxymethylene) which certainly improved the interfacial layer on Si anode. Electrochemically, a two‐stage reduction reaction occurs with FEC, and further reactivity of poly(VC) with Me_3_SiLi leads to poly(hydroxymethylene); a polymeric SEI with C═O bonds were formed at 0.9 V (versus Li/Li^+^) followed by transition to C–O bonds at 0.6 V.^[^ ^103^, ^104^ ^]^

The development of polymer based organic SEI layer not only maintained the functionalities of SEI but also remained robust to particle stress related volume changes of the active materials. Jin et al. investigated the effect of pure VC and FEC as an electrolyte for Si nanowire (Si NW) electrodes with an aim on understanding the electrochemical performances and the nature of chemical species developed over the surface and interphase of silicon. Studies through ^13^C NMR (nuclear magnetic resonance) substantiated that the organic layer developed through the decomposition of VC and FEC mainly constituted cross‐linked poly(ethylene oxide) (PEO) and alkyl chains, which comprised about 15% of the carbon content in the organic layer of SEI film. The unavoidable oxide‐based surface contamination like SiO*_x_* and the hydroxyl‐terminated SiO*_x_* on the surface of Si gradually reduced forming organosiloxane species containing Si–C bond. The progression of siloxane groups with Si–C species denoted that the SiO*_x_* groups were bonded with organic layer along with the dominant inorganic Li salts on the Si electrode's surfaces. Significant benefits that arise through the development of cross‐linked PEO boost the cyclability of Si NW electrode, i) limit solvent permeation and swelling of SEI film, ii) control the volume related stress of silicon wires, and iii) better Li‐ion conductivities than the linear PEO observed in EC‐based electrolyte.^[^ ^105^, ^106^ ^]^ In addition to the functional additive's role in stabilizing the SEI on Si anode, the effect of controlling the working voltage limit to preserve the local environment of SEI certainly benefited improved cycling performances.^[^ ^107^ ^]^ Further, the consumption of FEC and its effect on the electrochemical performance of Si anode under the cut‐off voltage limitations were studied by Yamazaki et al. It was found that the high cut‐off voltage 2.0 V for the Li ǀǀ Si–G cells led to a decreased capacity retention, while lowering the cut‐off voltage to 0.5 V caused an increase in the capacity retention to 62% at the end of 200 cycles. The case appeared to be similar with Si–G ǀǀ LiFePO_4_ full cells, where the cut‐off voltage limit based on the Li (for example −3.452 V versus LiFePO_4_ which is equivalent to 0.002 V versus Li/Li^+^) followed the capacity retention at voltages 0.7 > 1.0 > 1.5 > 2.0 V as illustrated in Figure 7c. Meanwhile, the retention deviated for 0.5 V, which may be due to the discharge‐charge capacity differences between the anode and cathode. It was inferred that the consumption of FEC was more with an increase in the cut‐off voltage leading to formation, destruction, and continuous growth of SEI film. As a result, the stability of SEI is compromised at a high cut‐off voltage than the low cut‐off giving rise to variations in their capacity retentions.^[^ ^108^ ^]^

The influence of cathode material's structural properties during the electrochemical cycling also impacted the SEI film on the graphite anode.^[^ ^109^, ^110^ ^]^ For instance, the electrochemical cycling of spinel LiMn_2_O_4_ cathode suffer from severe Mn dissolution and migration. The Mn ions in the electrolyte solution migrate and penetrate to the graphite anode and occupy the edge planes of graphite layers.^[^ ^111^, ^112^ ^]^ Several film forming additives like VC, VEC, and FEC are still short of evading the Mn penetration and related concerns.^[^ ^113^, ^114^ ^]^ Pyridine based additives, namely 2‐vinylpyridine (VP) demonstrated an effective SEI formation on the graphite anode. For the full‐cell graphite ǀǀ LiMn_2_O_4_, the SEI modified via VP additive suppressed the deterioration of graphite anode due to the prevention of dissolved Mn‐ions from the LiMn_2_O_4_ cathode.^[^ ^115^ ^]^ Tracking the electrochemical fate of VP additive, they develop a responsive SEI structure toward the Mn dissolution. In addition to altering the SEI structure, another strategy operated through the direct reaction or interaction of additives with the dissolved Mn ions in the electrolyte aimed to suppress the ion migration. Cyclic ether such as 4,7,13,16,21,24‐hexaoxa‐1,10‐diazabicyclo[8,8.8]hexacosane (C222) as an additive interacted with the Mn‐ions in the electrolyte preventing (de‐)intercalation reaction into the graphite planes. Thus, the redox currents due to Mn (de‐)intercalation were reduced along with lower interfacial film resistance and charge transfer resistance.^[^ ^116^ ^]^ It is obvious that the compatibility of electrolyte and the electrode material is important, where the FEC based electrolyte demonstrated an improved cyclability for over 150 cycles with the Si anode against the LiNi_0.5_Mn_1.5_O_4_ cathode.^[^ ^117^ ^]^

The concentration dependent co‐intercalation behavior of PC with different Li salts, which include LiClO_4_, LiPF_6_, and LiN(SO_2_C_2_F_5_)_2_ was studied by Jeong et al. The electrochemical results inferred that at high electrolyte concentration, i.e., 3.27 mol kg^−1^ of LiClO_4_ and 2.45 mol kg^−1^ of LiPF_6_ and LiN(SO_2_C_2_F_5_)_2_, respectively the Li‐ions from the electrolyte intercalated with graphite forming lithium–graphite intercalation compound (Li–GIC) leaving no evidence for solvent co‐intercalation, while this was apparent at low electrolyte concentrations.^[^ ^118^, ^119^ ^]^ It was reported that the compatibility of PC with graphite anode could be improved by employing a high concentration electrolyte with no requirements for any film forming additives. However, a significant rise in the concentration of liquid electrolyte inadvertently increases the viscosity of electrolyte, which might not only limit ionic mobility but also suppress the formation of a stable SEI layer.^[^ ^120^ ^]^ Additives actively employed in electrolyte formulation could also alleviate the effects imposed by the nature of solvent, concentration, and related viscosity. Alongside, the formation of SEI layer constituting an organic/polymeric layers is highly beneficial to improve the mechanical stability of films, thereby the fracture of anode materials during the electrochemical cycling could be avoided.^[^ ^121^, ^122^ ^]^ Abe et al. reported a series of olefin compounds such as vinyl acetate (VA), divinyl acetate (ADV) and ally methyl carbonate (AMC) as an additive formulated with PC‐based electrolytes for Li‐ion batteries. The electrolyte decomposition in the presence of additive was investigated using three different carbons, namely natural graphite (NG), graphitized mesophase carbon microbeads (MCMB 6–28), and graphitized mesophase carbon fiber (MCF). It was found that the degree of graphitization influenced the cyclability demonstrating that the nature of SEI film differed with the type of graphitic carbon and crystallinity. Establishing a stable SEI via additive reduction, the solvent co‐intercalation into the graphite structure in the presence of PC‐based electrolyte was avoided. In principle, prior to the decomposition of PC solvent, the olefinic additives VA, ADV, and AMC underwent prompt decomposition on the graphite surface forming a stable SEI layer which acted as a barrier to prevent the co‐intercalation.^[^ ^29^ ^]^ Derivatives of butyrolactone were used as an additive in LiClO_4_–PC electrolyte to mitigate the solvent co‐intercalation and exfoliation of graphite. Studies through ^13^C NMR exposed that the suppression of solvent co‐intercalation and decomposition was owed to a decrease in the coordination of PC molecules with Li‐ions and an increase of butyrolactone coordination.^[^ ^123^ ^]^

The apparent role of additives in developing a stable and robust SEI promoting the electrochemical performances of both the anodes, existing graphite and next‐generation high‐capacity silicon anode was prominent. Carbonate additives like VC, FEC, and VEC showed amenable film‐forming ability on the graphite anodes. The reduction of carbonate additives at the graphite surface developed a thin and insoluble interphase composed of reduced products lithium carbonate, lithium carboxylates, and polymeric compounds. For example, the SEI developed in the presence of VC and FEC showed minimal difference in the LiF content, which certainly boosted the ionic conductivity of the FEC constructed interphase. Sulfur‐containing additives like 1,3‐propane sultone and ethylene sulfite were effective in facilitating Li‐ion intercalation rather than solvent co‐intercalation due to the SEI rich in lithium sulfates and lithium alkyl sulfates. Considering high‐capacity Si anodes, the physical properties, electrochemical mechanism, operating voltage, capacity, etc., differ largely with the graphite anodes. Most of the Si anodes are composites with carbon, thus the additives suitable for graphite like VC and FEC have shown promising performances. However, the huge volume changes of Si anodes necessitated the need for additives favoring the construction of polymeric SEI. Hence, a diverse understanding of the role of additives must be clearly sorted on the basis of theoretical and experimental studies. Since most of the carbonate‐based electrolyte formulations were engineered suitable for the graphite anode, adoption of similar formulations, additives, and solutions rendered with any anode material could not be directly executed to other anode materials. Undoubtedly, the domination of graphite as an anode powering millions of electronic portable gadgets to smart e‐mobility is lively, an improvement toward increasing the specific capacity and energy density is meanwhile demanding. Thus, solid solutions to develop Si and/or Sn anodes through mitigating the volume expansion/contraction and extended cycle life, robust polymeric SEI with blended ionic and mechanical properties are much needed for the next‐generation high‐energy density batteries. At the same time, the dependence of graphite anode must also be carried out to fill the gap of energy and power demand for various electronic applications. However, a lot of concerns like structural deterioration, metal‐ion co‐intercalation, and extended cycle life need to be understood.

#### 4.1.2 Cathode Electrolyte Interphase

**Figure 8:**
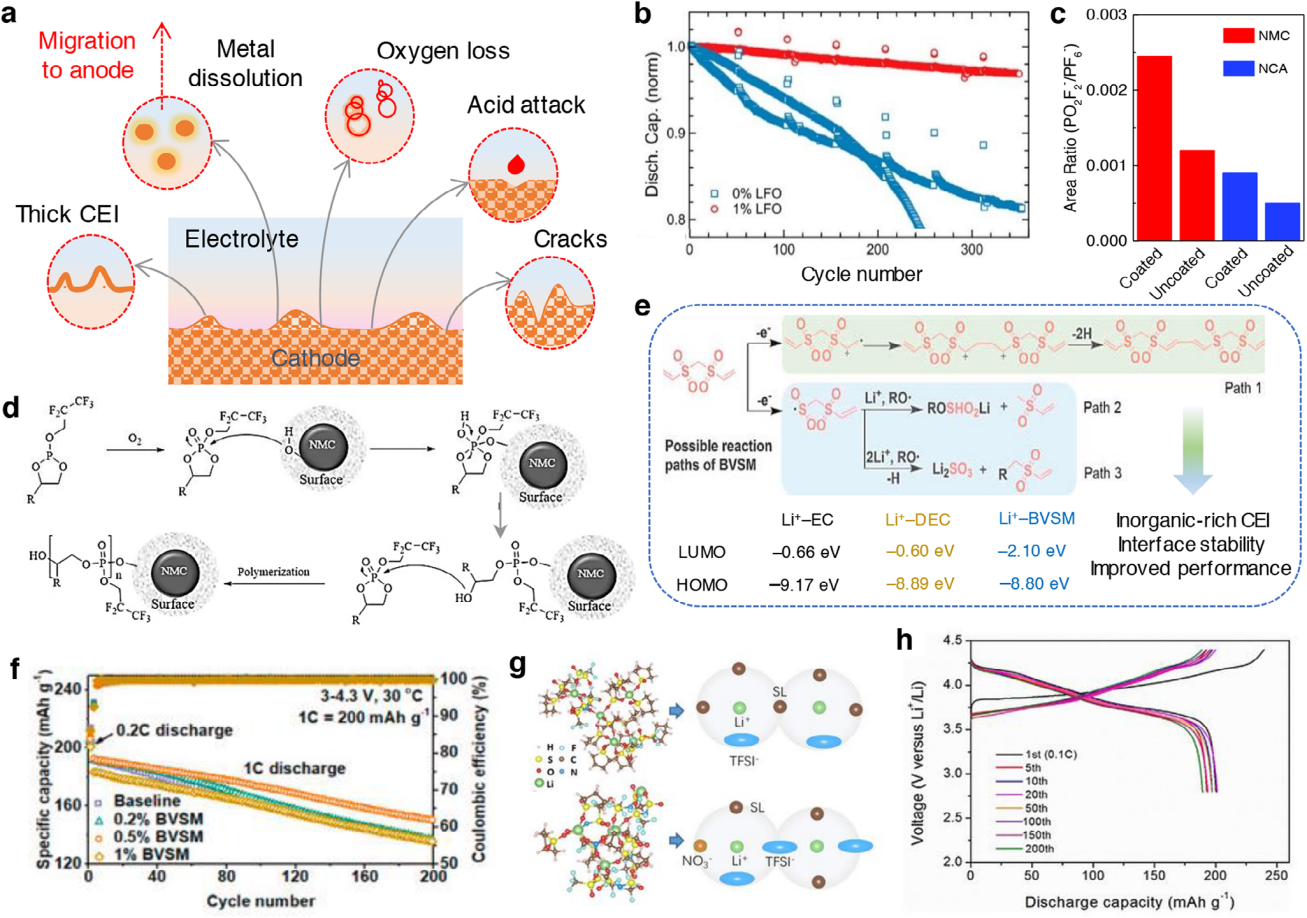
a) Schematics representing the critical issues related to the cathode‐electrolyte interphase in lithiated‐transition metal cathode materials for Li‐ion batteries. b) Capacity retention of graphite ǀǀ NMC532 cells obtained at 0.33 C rate and 40 °C in the presence of LiPF_6_‐EC:DMC as blank and blank+LiPO_2_F_2_ containing electrolytes. c) Chart depicting the effect of electrolyte aging (2 weeks at 40 °C), the ratio of PF_6_^–^ and PO_2_F_2_^–^ species in an electrolyte with Al_2_O_3_ coated and uncoated NMC622 and NCA electrodes are analyzed. Reproduced with permission.^[^ ^138^ ^]^ Copyright 2019, American Chemical Society. d) Mechanism showing the mode of phospholane additive on the NMC cathode particles. Reproduced with permission.^[^ ^139^ ^]^ Copyright 2019, American Chemical Society. e) Possible reaction mechanism of bis(phenylsulphonyl)methane (BVSM) additive toward constructing a CEI film. f) Capacity retention of high nickel NMC cathode obtained with blank as LiPF_6_ in EC:DEC and various concentrations of BVSM additive containing electrolytes. Reproduced with permission.^[^ ^140^ ^]^ Copyright 2022, Elsevier. g) Scheme depicting the solvation structure of LiTFSI‐SL and LiTFSI+LiNO_3_‐SL electrolytes. h) Cycling performance of Li LiFePO4 cells with 3.25 M LiTFSI+LiNO_3_‐SL electrolyte formulation. Reproduced with permission.^[^ ^141^ ^]^ Copyright 2020, Wiley‐VCG.

The structural and interfacial instabilities of cathode materials upon continuous cycling deteriorate the performance resulting to a limited cycle‐life and safety risk of a Li‐ion battery. Layered lithium transition metal oxides, LiMO_2_ (M represent electrochemically active Ni, Mn, Co, etc. in combination with traces of Al, Ti, etc.) and olivine LiFePO_4_ are widely employed cathode materials for Li‐ion batteries owed to their practical capacity ≈200 mAh g^−1^ and ≈160 mAh g^−1^ and an output voltage of 4.3 V and 3.4 V (versus Li/Li^+^), respectively.^[^ ^124^, ^125^ ^]^ Upon multiple charge/discharge cycles, the LiMO_2_ cathodes succumb to capacity fade and voltage decay due to cycling‐induced structural degradation of layered structure. Concerns due to structural degradation of cathode as portrayed in **Figure** **8**a which includes phase transformation, metal dissolution, loss of active material, mechanical damage, thick SEI, cracks, etc., are primarily driven by the oxygen evolution and its loss from the crystal lattice of layered oxides. Thus, the oxygen framework being the fundamental support for the layered oxides gets collapsed with an altered chemical environment and performance degradation.^[^ ^126^, ^127^ ^]^ Hence, a prevalent retention of a formidable interphase on the cathode particles is expected to mitigate the concerns suffered by the layered cathode materials. Solutions pertaining to the development of a robust CEI and the measures to counter the evolution of gas are necessary to improve the battery performances and its lifetime. A separate section is devoted on the progresses related to controlling the gases, especially due to electrolyte decomposition and oxygen loss from the cathode material. This section explicitly deals with the development of a robust and stable CEI on the cathode surface in the presence of functional additives under normal cycling, at low and elevated temperature, and cut‐off voltage cycling limitations.

Lithium cobalt oxide as a cathode material have been used in most of the commercial Li‐ion batteries due to its high theoretical capacity of 274 mAh g^−1^ and energy density of 350 Wh kg^−1^ at a cut‐off 4.2 V. The intrinsic voltage limit of layered LiCoO_2_ allows about 0.5 moles of Li per LiCoO_2_ to be reversibly extracted which leads to a capacity of about 140 mAh g^−1^. The above limitations are owed to structural instability and irreversible phase transition from the hexagonal to monoclinic structure (O3 to H1–3) with a significant drop in the *c*‐lattice parameter of LiCoO_2_ at the voltage above 4.55 V.^[^ ^128^, ^129^ ^]^ Goodenough et al. exposed the existence of the CEI on the surface of layered LiCoO_2_ (0 < x < 0.5) as a function of state‐of‐charge (SOC). The respective changes observed in the interfacial resistance of a de‐lithiated Li_0.1_CoO_2_ were ascribed to a thin amorphous layer ≈3 nm known as CEI film. It was noted that upon an extraction of Li ≥ 0.35 from the LiCoO_2_ structure, the Co^4+^ oxidize the electrolyte solvent PC to develop a polymer shell around the active particles.^[^ ^130^ ^]^ Abe et al. reported biphenyl, *o*‐terphenyl, and *m*‐terphenyl organic additives to improve the electrochemical performance of LiCoO_2_, especially at a high voltage limit. The low oxidation potential of phenyl‐based additives than the organic electrolytes assist in establishing a conductive surface protecting the LiCoO_2_ particles from the electrolyte decomposition. In order to maintain a thinner and less resistive CEI film, the amount of phenyl‐based additives must be kept below 0.2%.^[^ ^131^ ^]^

Inorganic material like Al_2_O_3_ was reported as an additive to improve the electrochemical performances of LiCoO_2_ cathode material. The nano‐Al_2_O_3_ particle containing electrolyte LiPF_6_ in EC:DMC does react with prevailing HF and PF_5_ species to develop Li_3_AlF_6_ species. As a result, the carbonate electrolyte suffered from an increased acidity and reactivity promoting surface deterioration. It was concluded that the corrosion effects due to the reaction of as‐formed AlF_3_/Al_2_O_3_ and AlF_3_/Li_3_AlF_6_ species toward the insulative alkaline species over the surface of LiCoO_2_ particles provided an enhanced ionic conduction of CEI film.^[^ ^132^ ^]^ Multifunctional additive based on phosphate‐functionalized ionic liquid (PFIL) was reported by Ge et al. The modified electrolyte LiPF_6_ in EC: DMC formulated with 5 wt.% PFIL improved the thermal stability with the self‐extinguishing time brought from 196 s g^−1^ to 130 s g^−1^. The cycling stability of Li ǀǀ LiCoO_2_ cells were improved with the modified electrolyte with 92.7% retention of initial capacity which may be due to improved Li‐ion mobility due to coordination between Li‐ions and PFILs.^[^ ^133^ ^]^ Previous reports on PFIL additive showed an improvement in the electrochemical performance of LiFePO_4_ cathode validating the coordination mechanism of Li‐ions and PFILs to improve the properties of olivine cathode.^[^ ^134^ ^]^ Meanwhile, the lower operating voltage of LiFePO_4_ (3.4 V versus Li/Li^+^) cathode surpasses the issues related to oxidative stability of electrolytes, however, the dissolution of iron is still a concern. Thiolane based 4‐propyl‐[1,3,2]dioxathiolane‐2,2‐dioxide (PDTD) as an additive works through the stabilization of cobalt ions that are dissolved in an electrolyte solution. The strong coordination of Co and PDTD strategically prevented the deposition of cobalt metal on the anode and prevented the catalytic decomposition of electrolyte solution in the presence of metal ions. Electrolyte containing 1 M LiPF_6_ in EC:EMC:DEC (3:5:2 wt.%) with 1 wt.% PDTD additive tested with Li ǀǀ LiCoO_2_ battery showed significant electrochemical performance improvements like capacity retention and rate capability.^[^ ^135^ ^]^

Moving to derivatives of LiCoO_2_, combination of transition metals, layered lithiated nickel manganese cobalt oxides LiNi_x_Mn_y_Co_1‐x‐y_O_2_ (NMC) are a class of 4 V‐electrode materials employed as a cathode in commercial Li‐ion batteries. Based on the proportion of transition metals, the NMC‐based cathodes take the stoichiometry as follows, LiNi_0.33_Mn_0.33_Co_0.33_O_2_ (NMC111), LiNi_0.5_Mn_0.3_Co_0.2_O_2_ (NMC532), LiNi_0.6_Mn_0.2_Co_0.2_O_2_ (NMC622), and LiNi_0.8_Mn_0.1_Co_0.1_O_2_ (NMC811) showing unique electrochemical performances. The role of each transition metal in a layered NMC plays a significant role both structurally and electrochemically; Ni is the main redox active material furnishing high practical specific capacity, Mn provides good structural stability, and Co contributes to good cycle life. The combination of transition metals in NMC‐based cathodes offer the merits of high voltage stability up to 4.6 V, reversible capacity as high as 160 mAh g^−1^, good cycling life, and thermal stability.^[^ ^136^, ^137^ ^]^ Meanwhile, there are few concerns where the exposed surfaces of NMC cathodes are the sites for the decomposition of electrolyte, associated metal dissolution and capacity fading. Surface coating using metal oxides, carbon‐based and heteroatom doped carbon are some of the strategies explored to protect NMC cathodes.^[^ ^114^, ^126^, ^129^ ^]^ Electrolyte additive engineering is another strategy to improve the electrochemical performance of NMC‐based cathode materials. Hall et al. coated Al_2_O_3_ over LiNi_0.8_Co_0.15_Al_0.05_O_2_ and observed an increase in the cycling stability and lifetime. It was noted that the Al_2_O_3_ coated cathode particles experienced a spontaneous reaction with the LiPF_6_ in an electrolyte solution to produce LiPO_2_F_2_ species, which is a well‐known electrolyte additive to reduce the cell impedance and improve the cell performance in terms of capacity and cycle life (Figure 8b). The atomic ratio of PO_2_F_2_^−^/PF_6_ was found to be higher for the Al_2_O_3_ coated NMC and NCA cathodes denoting the readily reactive nature of LiPF_6_ with Al_2_O_3_ forming LiPO_2_F_2_ species (Figure 8c) benefitting an improved cell performance.^[^ ^138^ ^]^ It should be noted that the metal oxide‐based coating strategy comes with drawbacks such as controlling the composition and their low electronic and ionic conductivities. Aspern et al. reported that the introduction of –CF_3_ groups in cyclic phosphorous‐based compounds 2‐(2,2,3,3,3‐pentafluoropropoxy)‐1,3,2‐dioxaphospholane (PFFPEPi) forming 2‐(2,2,3,3,3‐pentafluoropropoxy)‐4‐(trifluoromethyl)1,3,2‐dioxaphospholane (PFPOEPi‐1 CF_3_) impacted the formation of CEI film. The sacrificial decomposition of phospholane via polymerization reaction on the surface of NMC811 developed CEI, while the presence of additive with (PFPOEPi‐1 CF_3_ showed increased resistance and lower electrochemical performance than the PFPOEPi additive. The phosphorous present in the additive interacted with the hydroxyl groups on the NMC111 surface resulting in a ring‐open reaction. Subsequently, the hydroxyl groups could react with additional phospholane molecule followed by polymerization reaction as portrayed in Figure 8d. It was noted that the insulative LiF deposition of NMC surface was more with –CF_3_ containing additive leading to decreased cell performance.^[^ ^139^ ^]^ It is worth denoting that the properties of LiF endorse a positive benefit on the surface of Li metal, while it does an adverse effect on the surface of NMC cathode particles. However, the diverse results with the –CF_3_ species on the formation of interphase needs a clearer understanding.

Bis(vinylsulfonyl)methane (BVSM) as an additive improved the electrochemical performances of Ni‐rich LiNi_0.90_Mn_0.05_Co_0.05_O_2_ cathodes. The electrolyte containing even 0.5% of BVSM additive certainly enriched the CEI with elements S, F, and C through a competitive coordination among the solvents, additive, and anion (Figure 8e). The resultant CEI exhibited lowered interfacial resistance and supressed both the metal dissolution and phase transition in Ni‐rich particles. Full‐cells graphite ǀǀ LiNi_0.90_Mn_0.05_Co_0.05_O_2_ containing BVSM‐based electrolyte (1% BVSM) (Figure 8f) demonstrated a capacity retention of 78% at the end of 200 cycles at 1 C rate.^[^ ^140^ ^]^ Fu et al. reported LiTFSI–sulfone‐based electrolyte using 1,1,2,2‐tetrafloraethy‐2′,2′,2′‐trifuoroethyl (HFE) as an additive. Interestingly, the sulfone‐based electrolyte formulation containing LiNO_3_ was capable of operating at a high‐voltage. Sulfone electrolytes are in general weak toward the formation of SEI on the Li metal, which could be improved by increasing the concentration of electrolyte. With highly concentrated electrolyte containing HFE additive, it was found that each Li^+^ in 3.25 M LiTFSI‐SL electrolyte was solvated by about 3.44 and 1.03 SL molecules and TFSI^−^ anions, respectively (Figure 8g). As a result, the modified formulation with HFE enabled the construction of a stable interphases SEI and CEI and improved the cyclability of Li ǀǀ LiNi_0.80_Mn_0.1_Co_0.1_O_2_ cells (Figure 8h). The electrolyte 3.25 M LiTFSI–SL containing LiNO_3_ showed high oxidation tolerance than the nitrate‐free electrolyte due to the SL solvent. The lower steady‐state oxidative decomposition of LiNO_3_–SL electrolyte than the electrolyte without LiNO_3_ indicated that the CEI films developed in the former case restrained further decomposition of electrolyte solution. The resistive nature of CEI (*R*_CEI_) studied through EIS was found to be high at 218.7 Ω for LiTFSI–SL electrolyte, while the introduction of LiNO_3_ reduced the resistance to 168.6 Ω. It was concluded that the dense CF_x_‐rich SEI developed in the presence of LiNO_3_ protected the CEI and reduced the accumulation of products bearing high‐resistance.^[^ ^141^ ^]^

**Figure 9:**
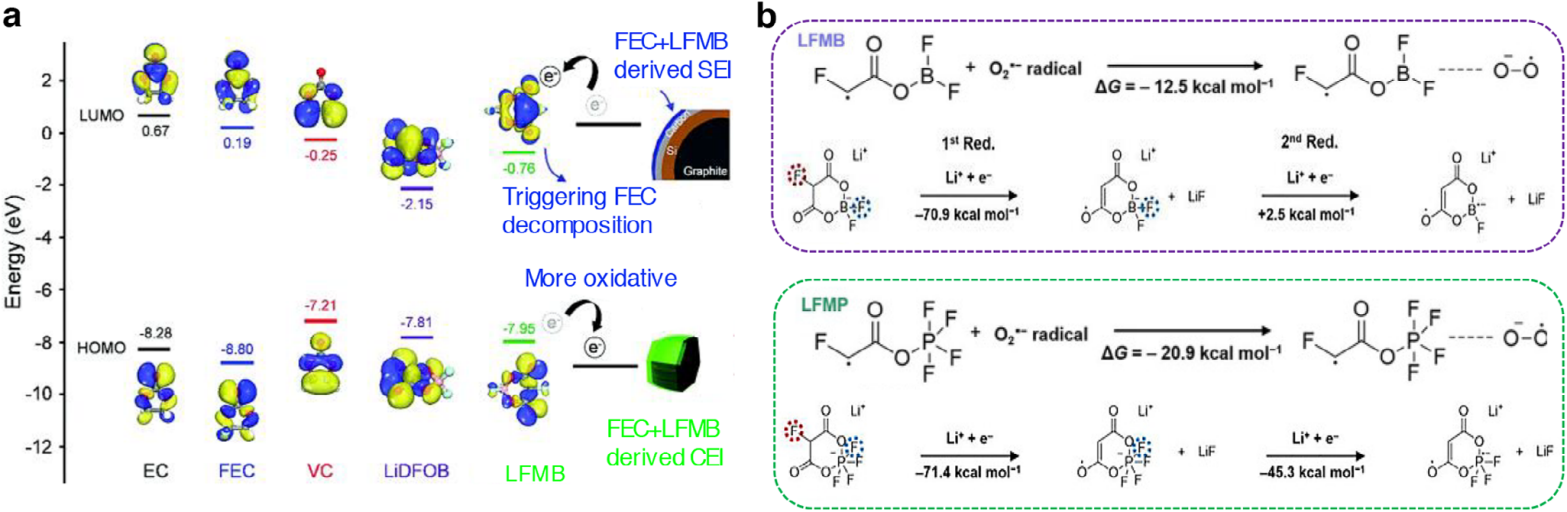
a) Energy level diagram of solvents EC, FEC, and VC and additives LiDFOB and LiFMDFB, followed by representation of interphase formed on both the anode and cathode in the presence of LiFMDFB and FEC. Reproduced with permission.^[^ ^146^ ^]^ Copyright 2018, Royal Society of Chemistry. b) Scheme depicting Gibbs free energy for the oxidative decomposition of LFMB and LFMP with superoxide radical. Reproduced with permission.^[^ ^147^ ^]^ Copyright 2022, Elsevier.

A series of perfluoroalkyl (PFA) substituted ethylene carbonates were developed as additives for Li‐ion batteries employing LiPF_6_ in EC:EMC electrolyte. The substituents attached to 1,3‐dioxolan‐2‐one are 4‐(trifluoromethyl) (TFM), 4‐(perfluorobutyl) (PFB), 4‐(perfluorohexyl) (PFH), and 4‐(perfluorooctyl) (PFO) were developed. Among the perfluoroalkyl substituted additives, PFO (0.5 wt.%) presented a better control over the degradation and extended the cycle life of graphite ǀǀ Li_1.2_Ni_0.15_Mn_0.55_Co_0.1_O_2_ batteries. The hydrophobic and lipophobic behaviors of perfluoroalkyl were exploited to form a double‐layered passivating layers on the Ni‐rich cathode's surface which not only prevented the surface degradation but also electrolyte decomposition. Typically, two components of perfluoroalkyl were involved in the construction of an inner and outer layer which was quite stable and impermeable to electrolyte solution. The inner layer evolved due to the reductive or oxidative decomposition of active head‐group of perfluoroalkyl attached to electrode surfaces and outer solvophobic layer formed due to self‐assembly of perfluoroalkyl chain constructed dual‐layered protection.^[^ ^142^ ^]^ Binary additives based on suberonitrile (SUN) and lithium bis(oxalate)borate (LiBOB) were employed to design LiPF_6_ in EC:DME (1:1 vol.%) electrolyte aimed to improve the LiCoO_2_ cathode's electrochemical performances. Primarily, the additive LiBOB gets oxidized prior to the decomposition of carbonate solvent and the higher oxidation potential of SUN offered better electrolyte stability at higher potentials. Further, the decomposition and oxidation roles of SUN–LiBOB binary electrolyte resulted in an altered CEI structure and its composition had a positive influence of LiCoO_2_ cells on the capacity retention about 62% and cycle life up to 500 repetitions.^[^ ^143^ ^]^ The fluorine‐free and high thermal stability of LiBOB endow a good control on the SEI and CEI properties; suppressed metal dissolutions like Mn and Fe and contributed to an SEI on graphite surface and the oxidation of borate‐salt at high potential developed a CEI containing borate and oxalate groups, known to inhibit the decomposition of alkyl carbonates at such potentials.^[^ ^143^, ^144^ ^]^ Ternary additives‐based electrolytes comprised of (2%PES + 1% methylene methane disulfoante (MMDS) + 1% tris‐(trimethylsilyl)‐phosphite (TTSPi)) exhibited notable improvement in graphite NMC442 cells. The additive combinations PES, MMDS, and TTPSi undergoes reaction on the surface of graphite and NMC442, while restricting the degradation of LiPF_6_ salt.^[^ ^145^ ^]^ Compared to Li salts possessing oxalate ligands, malonate‐based salts were also explored as additive for high‐voltage cathodes. Malonate‐based Li‐salts, lithium difluoro(fluromalonate)borate (LFMB) and lithium malonate (difluoro)borate (LMB) were studied as an additive for the Si‐G anode and Li‐rich cathode. The electrolyte containing LFMB in 1 wt.% LiPF_6_ in EC:EMC:DMC (3:4:3 vol.) with 5% FEC exhibited high energy density and rate capability than the blank electrolyte. Through molecular dynamic simulation, it was revealed that the low LUMO energy of LFMB than the FEC (**Figure** **9**a) imposed a prior decomposition of malonate borate salt. In addition, the electron withdrawing character of boron were conducive for the formation of stable CEI film.^[^ ^146^ ^]^ Park et al. synthesized lithium tetrafluro(fluromalonate)phosphate (LTFMB) and studied its role on the electrochemical performance. The LTFMP additive engineered the interface of both the anode and cathode to deliver an improved capacity retention of 52% after 250 cycles, while 13% was shown by LFMB electrolyte‐based graphite ǀǀ NMC811 cells. Precisely, the CEI derived through the LFMP containing phosphorus (ΔG = −20.9 kcal mol^−1^) as shown in Figure 9b demonstrated a better affinity toward the superoxide radical than the boron (ΔG = −12.5 kcal mol^−1^) in LTFMB additive. Alongside, the formation of LiF was more favourable for the LTFMP due to weak P–F bonds in LFMP than the B─F bonds of LFMB.^[^ ^147^ ^]^ The effect of various Li salts associated with the formation of SEI were studied in relation to the aging properties of Li‐ion batteries. A chelation‐based mechanism using glyme compounds CH_3_O(CH_2_CH_2_O)*_n_* CH_3_ (G*_n_* with *n* = 2, 3 or 4) able to coordinate with the Li‐ions in the electrolyte were investigated by Chretien et al. The high donor numbers and a relatively strong Lewis basicity imparted via lone pair on oxygen atom of glyme molecules effectively chelated Li‐ions. It was reported that the Li salts Li_2_CO_3_, LiOCH_3_, and LiOC_2_H_5_ offered beneficial impact on the SEI formation, whereas LiOH and Li_2_O showed negative impact. It was found that the chain length of glyme molecules determines the strength of chelation with Li‐ions which was dependent on the nature and concentration of Li‐salt. A higher degree of complexation corresponds to a high chain length of glyme molecules leaving no more Li‐ions in the solution which resulted to a drastic decrease in the reversible capacity.^[^ ^148^ ^]^

**Figure 10:**
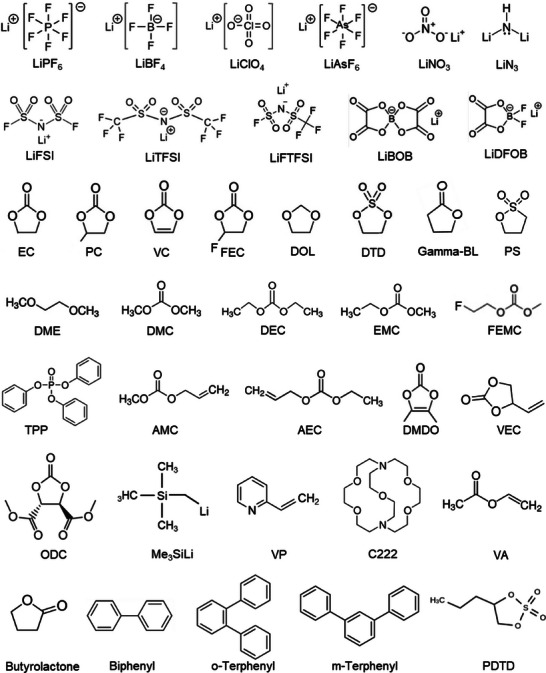
Chemical structures of salts, solvents, and functional additives for Li metal and Li‐ion batteries.

A carbonate‐free sulfone as an electrolyte solvent in combination with lithium bis(fluorosulfonyl)imide (LiFSI) was reported by Alvarado et al. for high voltage graphite ǀǀ LiNi_0.5_Mn_1.5_O_4_ batteries. The synergistic combination of LiFSI salt dissolved in tetramethylene sulfone or sulfolane solvent enabled stable interfacial SEI and CEI films. Tracking the evolution of SEI in sulfone–LiFSI electrolyte system revealed a stepwise reduction of LiFSI and sulfone resulting in the construction of SEI film. Typically, the reduction of FSI^−^ anion at higher potential of >2 V versus Li/Li^+^ developed a LiF‐rich surface followed by a lower potential sulfone reduction at ≈0.4 V. The developed SEI composed of dominant LiF and Li_2_O species which significantly favoured the Li‐ion intercalation. Owed to the high oxidation potential of sulfone, the complexation of Li‐ion and its polymerization established a stable CEI which prevented the dissolution of metal and were impermeable to solvent providing long cycle life.^[^ ^149^ ^]^ Nitriles such as succinonitrile (SN), adiponitrile (AN) and pimelonitrile (PN) were investigated as electrolyte additives for various NMC stoichiometric cathodes at high temperature. The electrolyte containing nitrile does not show any effect on the cycling performance of graphite ǀǀ NMC111 batteries at a cut‐off voltage of 4.2 V versus Li/Li^+^. Upon an increase in the cut‐off voltage to about 4.5 V, the graphite ǀǀ NMC442 cells infiltrated with an electrolyte containing 2 wt.% SN and 2 wt.% VC experienced severe capacity loss and gas generation. The use of nitrile‐based additives certainly increased the cell resistance, however the study determined that the addition of additives in combination with sulfite/sulfate or phosphite‐based compounds such as either ethylene sulphite or trimethylene sulfate with tris(trimethylsilyl)phosphite (TTSPi) could reduce the cell resistance.^[^ ^150^ ^]^ Numerous electrolyte additives were developed in the form of Li‐salts, organic solvents and ionic liquids and characterized their properties in carbonate‐ and ether‐based electrolyte solutions are discussed and some of them are represented in **Figure** **10**.

Structural deterioration of cathode materials upon cycling remained a prime target for the additives via conditioning their interfaces. The mode of action varied with the type of additives; i) an early oxidation decomposition than the electrolyte solvent and ii) direct interaction of dissolved metal in an electrolyte solution, while both the strategies aimed in constructing a robust and conductive CEI layer. Diverse cathode material's chemistries deal with their own challenges, thus selecting a suitable additive is pivotal. Organic additives phenyl and thiolane were affable for LiCoO_2_ cathode, whereas additives designed using dioxaphospholane, malonate phosphate, sulfonyl methane, fluoroalkyl, malonate borate, and nitriles altered and stabilized the CEI of LiNi_x_Mn_y_Co_1‐x‐y_O_2_ cathodes. Some of the additives like nitrile and sulfones and concentrated formulation with phenyls constructed a resistive CEI film. However, the combinations of additives like sulfone/TFSI^−^, sulfite/sulfate or phosphor, LiNO_3_, etc., reformed to conductive CEI reflected in the cycle life. Dual interfacial additives such as (fluromalonate)phosphate, nitrile+bis(oxalate)borate, etc., favoured a robust and high‐performance SEI and CEI films, such additives are advantageous in the practical consensus of lowering the concentration of total additives and indeed cost‐effective. Mechanistically, additive‐driven strategies diminished the dissolution and migration of metal ions from the cathode structure, while preserving the surface of cathode particles from further exposure to the electrolyte. Alongside, the type of species accumulated on the interface facilitated Li‐ion movement and also strengthened the interface to be robust during cycling at normal and at elevated voltage and temperature limits. More focus on the interfacial chemistries of cathode and anode materials developed in the presence of functional additives, the effect of limiting factors such as voltage and temperature is worth investigating.

#### 4.1.3 Lithium Metal Passivation

**Figure 11:**
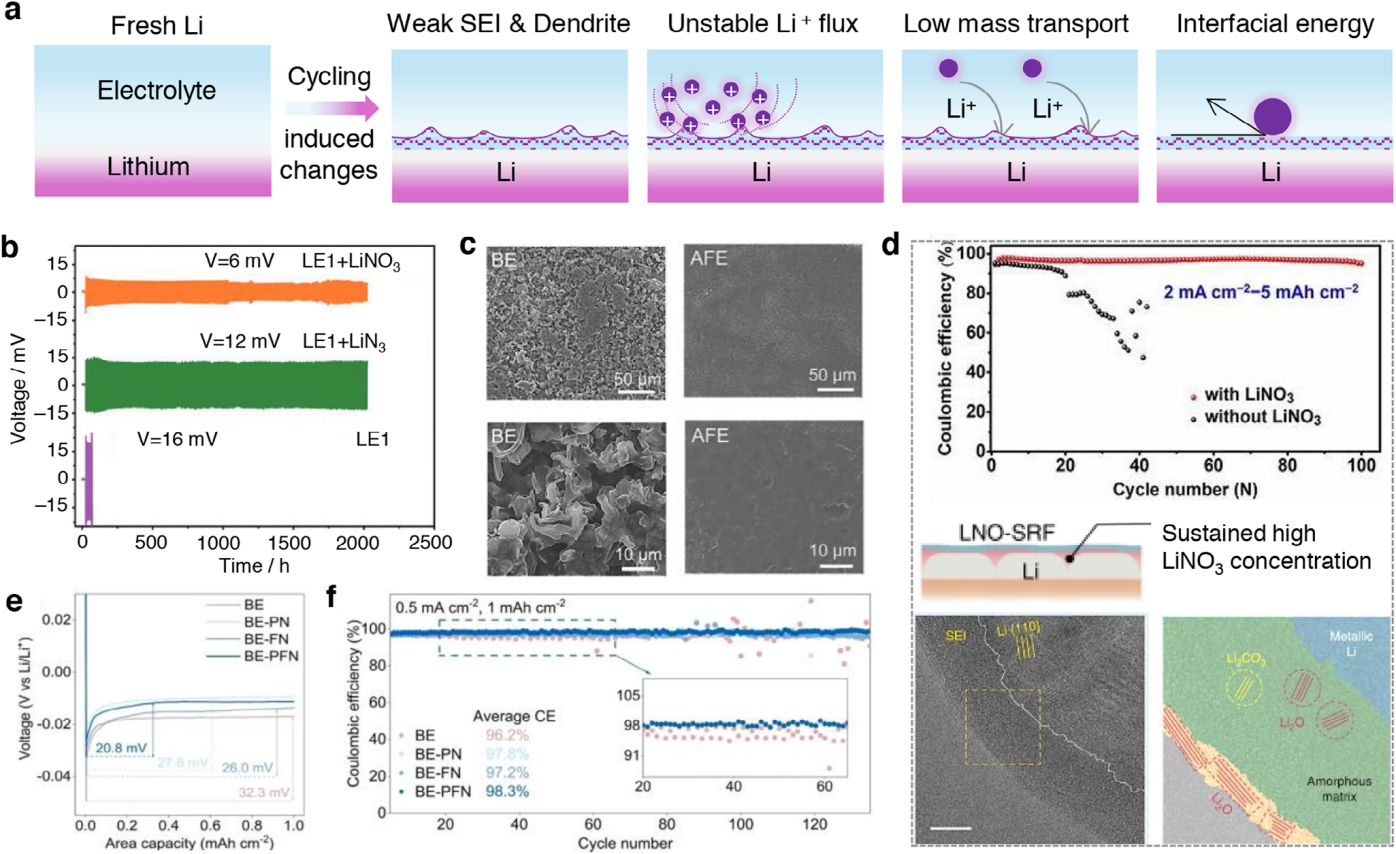
a) Schematics representing critical challenges in long‐term cycling of Li‐metal as an anode for high energy density batteries. b) Overpotential of Li anode studied through Li plating/stripping in the presence of electrolyte containing LiNO_3_ and LiN_3_ additives. Reproduced with permission.^[^ ^160^ ^]^ Copyright 2017, Wiley‐VCH. c) SEM image depicting the surface morphology of Li deposition in blank electrolyte and AFE‐additive based electrolyte. Reproduced with permission.^[^ ^163^ ^]^ Copyright 2022 The Authors, Springer Nature. d) Cycling stability of Li ǀǀ Cu cells tested in carbonate electrolyte and LiNO_3_ additive‐based carbonate electrolyte; Reproduced with permission.^[^ ^169^ ^]^ Copyright 2018, National Academy of Sciences, cryo‐TEM images of bilayer interphase developed through the sustained release of LiNO_3_ additive in a carbonate electrolyte. Reproduced with permission.^[^ ^170^ ^]^ Copyright 2018 The Authors, Springer Nature. e) Nucleation overpotential difference in various electrolyte formulations. f) Li plating/stripping of Cu ǀǀ Li cells at a current density of 0.5 mA cm^−2^. Reproduced with permission.^[^ ^171^ ^]^ Copyright 2024, Wiley‐VCH.

Lithium metal as an anode in any non‐aqueous electrolytic solution tend to react and form an interfacial film known as “SEI”, which primarily constitutes layers of inorganic and organic compounds as illustrated in Figure 2. The SEI layer due to the electrolyte decomposition are about few nanometres during the initial cycles which grow over repetitive cycling to a thick and dense structure. It is understood that the Li deposition behavior vary with the type of electrolyte solution, the most studied carbonate and ether‐based electrolyte systems. Ether electrolytes possess a good compatibility with the Li metal than the carbonate solvents leading to a diverse physical–chemical–mechanical properties of SEI and electrochemical performances.^[^ ^151^, ^152^, ^153^ ^]^ The collective root‐cause for limited cyclability of Li metal are illustrated as **Figure** **11**a, where the primary concerns identified are; dendrite growth, weak SEI, unstable/inhomogeneous Li^+^ flux, low ion transport, and poor interfacial energy (copper or anode‐less batteries). Over an extended cycling, the Li metal develops a needle‐like growth termed as “dendrites” which is identified to cause safety concerns. Several strategies which include surface modification, artificial SEI, restructuring SEI, host, lithiophilic material, electrolyte modifications etc., are actively developed to overcome the aforementioned concerns with Li metal.^[^ ^154^, ^155^, ^156^ ^]^ Specific to the discussion on liquid electrolyte, majority of the research efforts in retrospective to electrolyte additives aim on regulating the morphology of Li deposition and mitigating the loss as “dead Li”.

Lithium nitrate (LiNO_3_) is the most widely considered additive for an ether‐based electrolyte to stabilize Li metal and achieve uniform Li‐ion deposition. As a sacrificial additive, the LiNO_3_ gets reduced on the metal surface and develop an insoluble Li_x_NO_y_/LiN_x_O_y_ species forming a part of SEI constituents. In particular to Li─S batteries, the role of LiNO_3_ is more pronounced, where the Li_x_NO_y_ and Li_x_SO_y_ species generated due to the oxidation of nitrates and sulfides construct a passivation layer which certainly restricts the flow of electrons from the Li metal to an electrolyte solution.^[^ ^157^, ^158^ ^]^ Apart, LiNO_3_ as an additive supports a cost‐effective solution in terms of scalability and industrial viability. Other than the role of LiNO_3_ as an SEI promotor, Ye et al. demonstrated that the utilization of active sulfur and the preservation of carbon matrix could also be improved in the presence of an optimum amount of LiNO_3_ salts. Thus, the role of concentration of LiNO_3_ must be appropriately regulated to optimize the properties of both the Li metal anode and sulfur cathode to achieve better electrochemical performances.^[^ ^159^ ^]^ Lithium nitrate is often used as an additive in LiTFSI–DOL:DME electrolyte, which is a specific electrolyte recipe for Li─S batteries. The effect of lithium azide (LiN_3_) and lithium nitrate (LiNO_3_) as dual additives for stabilizing Li metal was studied as a combination with either LiTFSI or LiFSI based electrolytes. Ionic membranes developed with 2 wt.% LiN_3_ and LiNO_3_ containing LiTFSI and LiFSI showed a minimal voltage hysteresis and prolonged cycling stability (Figure 11b). Surface analysis revealed that the LiN_3_‐based electrolyte developed a smooth morphology over Li metal due to surface enriched Li_3_N species, while the existence of Li_2_O and Li_3_N compounds propagated to a fibre or needle‐like surfaces in the presence of LiNO_3_‐based polymer electrolytes.^[^ ^160^ ^]^ Indisputably, the role of LiNO_3_ toward a stable SEI favoured uniform Li deposition and metal protection in Li–S batteries. However, cell chemistries employing a high‐voltage lithiated cathode such as Ni‐rich, Li‐rich compounds, etc., coupled with a high energy density Li metal anode require robust high‐voltage electrolytes. Ester‐based carbonate electrolyte endows a high voltage limit achievable up to 4.3 V, however they are not so compatible with the Li metal. In addition, the sparse solubility of LiNO_3_ in carbonate‐based electrolytes (≈800 ppm) largely hinders its viability in developing carbonate‐based electrolytes for high energy density Li–metal batteries.^[^ ^161^, ^162^ ^]^

Zhang et al. engineered a carbonate‐based LiPF_6_ electrolyte through a combination of dual additive aluminum ethoxide (AlEtO_3_) and FEC solvent. In the presence of Al‐ethoxide, a smooth surface morphology and uniform deposits were observed as portrayed in Figure 11c, which is in contrary to the dense and dendritic Li deposits observed in the blank electrolytes. A robust and ionically conductive interphase were constructed on the Li metal anode and Ni‐rich cathode through the Al and Al_2_O_3_ by‐products generated via the decomposition of dual‐additives. Further, the Li metal coupled with NMC811 cells containing a modified electrolyte demonstrated a capacity retention of 80.3% and an energy density of 350 Wh kg^−1^.^[^ ^163^ ^]^ The film forming solvent‐based additives such as vinylene carbonate, fluoroethylene carbonate, and ethylene sulphite that were successful for the graphite anode were investigated for the Li metal anode. Mogi et al studied the film formation and Li dissolution ability of the electrolytes in the presence of VC, FEC, and ES molecules. Among the formulated electrolytes containing LiClO_4_ in PC with an individual formulation containing additive VC, FEC, and ES, the electrochemical performances of 5 wt.% FEC showed improved C.E. In contrary, the VC or ES additive based electrolytes suffered a lower efficiency. It was noted that the interfacial films developed with (PC+VC) or (ES+PC) possessed a higher resistance than the (PC+FEC) electrolyte combination. The film forming ability, uniformity, and close packed surface morphology developed in the presence of FEC was conducive for improving the efficiency of Li metal over an extended cycle‐life. In contrary, a more solid and inhomogeneous solid particle were observed for the VC and ES additives, respectively.^[^ ^164^ ^]^ Zheng et al. combined VC with lithium difluorophosphate (LiDFP) to develop a robust film on Li metal which upon modification possessed good mechanical and ionic properties. The introduction of LiDFP slowed down the VC molecule's reduction, meanwhile promoted the breakage of P–F bonds developing a stable and uniform protective layer. It was noted that the decomposition of LiDFP additive developed surface film rich in inorganic compounds LiF–Li_3_PO_4_ and along with lithium ethylene bicarbonate afforded an improved mechanical stability and ionic conductivity. The Cu ǀǀ Li cells containing LiClO_4_ in EC:EMC and 2 wt.% of each LiDFP and VC additives exhibited a C.E of 95% at the end of 80 cycles and also reflected a lower overpotential in Li symmetrical cells.^[^ ^165^ ^]^ Further, LiDFP in combination with LiNO_3_ was reported to develop an inorganic‐rich SEI containing LiF and Li_3_N species. The reformed inorganic‐rich SEI developed on the Li metal due to modified electrolyte offered a high C.E of 98% and cyclability over 700 h for the Cu ǀǀ Li and Li symmetrical cells, respectively.^[^ ^166^ ^]^

Other than triggering the property of additives through its decomposition or altering the solvation sheath, Shen et al. proposed a quinone‐based lithiophilic additive which was capable of forming an additive‐based Li salt to effectively regulate the Li deposition behavior. It is known that the faster kinetics and the formation of amorphous layer are quite interesting properties exemplified by the quinone‐based structures. Tetracholoro‐1,4‐benzoquinone (TCBQ), as an additive with a lowest LUMO energy than the EC and DEC solvent molecules largely showed reactivity with Li metal and gets reduced to Li_2_–TCBQ salt. A good interfacial stability with Li metal was demonstrated by TCBQ and its salt Li_2_–TCBQ. The Li cells containing 0.5 wt.% TCBQ formulated with LiPF_6_ in EC:DEC electrolyte demonstrated a stable Li plating/stripping behavior even at a high current density of 5 mA cm^−2^ and a good control over Li dendrites being guided through the lithiophilic nature of Li_2_–TCBQ salt.^[^ ^167^ ^]^ The organic‐dominated SEI film, especially in the carbonate‐based electrolyte possessed a low interfacial energy with the Li metal and resulted in a high resistance and low C.E. Enriching the surface through inorganic species were undertaken by Piao et al. through FEC, LiNO_3_, and sulfolane additives. The Li metal anodes wetted using carbonate‐based electrolyte 1 M LiPF_6_ in EC:DMC containing additives exhibited a high C.E of 99.6% and a capacity retention of about 90%. Synergistic interactions of LiNO_3_ and FEC additives certainly altered the solvation structure of 1 M LiPF_6_ in EC:DMC electrolytes and rooted to inorganic LiF–Li_3_N species. It is widely understood that the solubility of LiNO_3_ is low in carbonate solvents (<10^−5 ^g mL^−1^) as the Lewis basicity of carbonate solvents are inadequate to break the Li^+^ and NO_3_^−^ electronic interaction. Thus, the use of sulfolane additive's strong affinity toward Li‐ion helped to improve nitrate's solubility. The inorganic enriched compounds observed in the inner SEI provided combined properties; electronic insulation and better interfacial energy of LiF and high Li–ion conductivity of Li_3_N afforded better C.E and cyclability to Li metal anode.^[^ ^168^ ^]^

**Figure 12:**
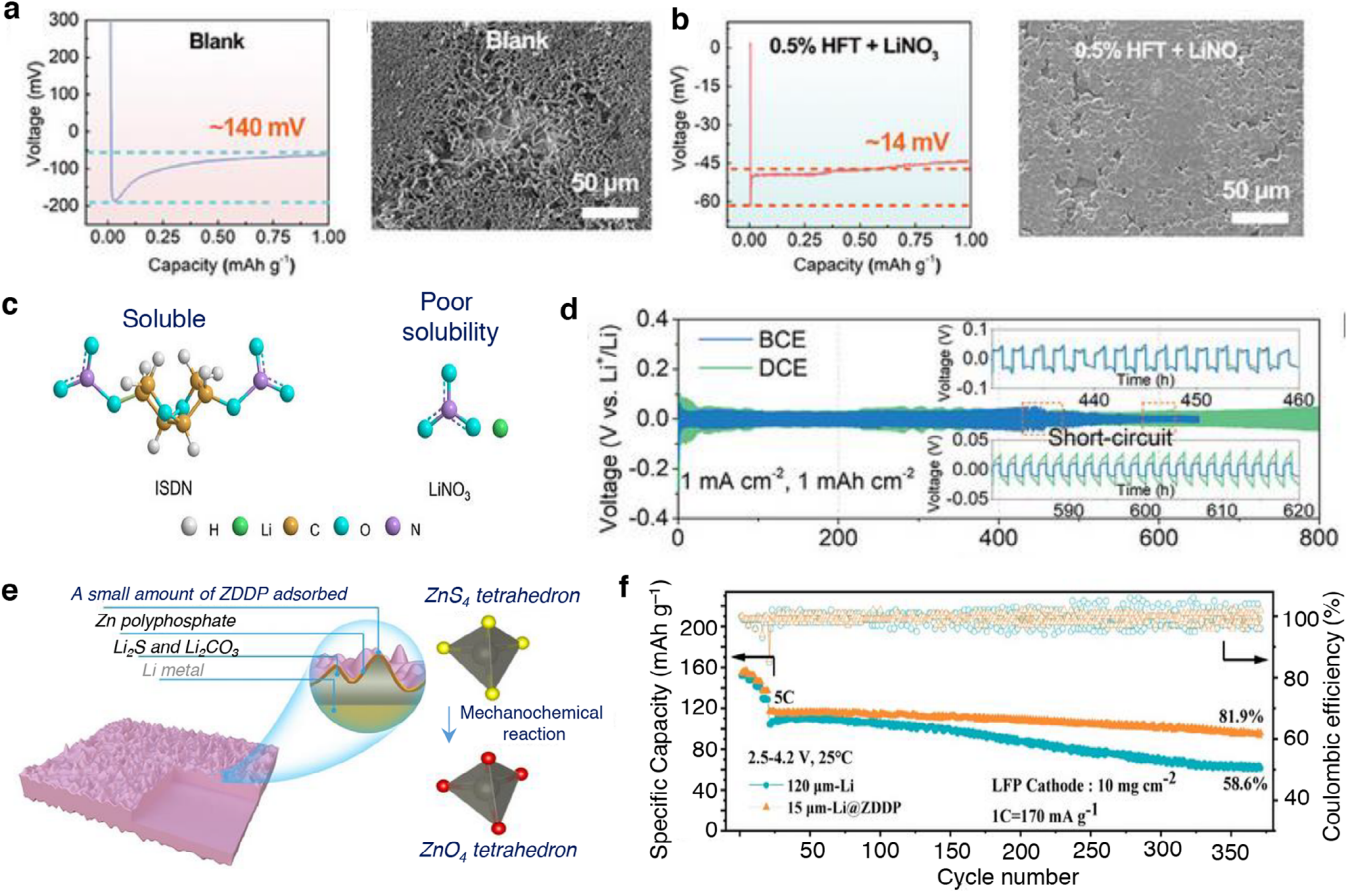
a,b) Lithium nucleation studies and respective SEM micrograph depicting the surface morphology of Li deposition in a blank and HFT+LiNO_3_ electrolyte, respectively. Reproduced with permission.^[^ ^172^ ^]^ Copyright 2024, Wiley‐VCH. c) Molecular structure of additives ISDN and LiNO_3_, where ISDN showed better solubility in a carbonate electrolyte. d) Lithium plating/stripping studies of symmetric Li cells tested at 1 mA cm^−2^ for a capacity of 1 mAh cm^−2^ for the blank and DCE electrolytes. Reproduced with permission.^[^ ^176^ ^]^ Copyright 2023 The Authors, Wiley‐VCH. e) Schematics representing the layers and composition of a strengthened Li metal anode *via* ZDDP additive and the respective phase changes of ZnS_4_ to ZnO_4_ upon mechanical rolling. f) Cycling stability of pristine Li and modified Li metal anode against high‐mass loaded LiFePO_4_ cathode at a current rate of 5 C. Reproduced with permission.^[^ ^181^ ^]^ Copyright 2023 The Authors, Springer Nature.

Shi et al. showed that the sustained release of LiNO_3_ in a LiPF_6_ in EC:DEC (1:1 vol. ratio) electrolyte enabled Li to undergo deep cycling at higher capacity > 10 mAh cm^−2^. The Li cells containing LiNO_3_ additive showed C.E's of 95.1% and 98.3% at 2 and 5 mAh cm^−2^, respectively (Figure 11d), where the nitrate‐free cells could possibly cycle for 30 cycles with low C.E's. Mechanistically, the sustained release of LiNO_3_ due to its limited solubility in carbonate‐based electrolyte underwent a slow decomposition and generated Li_3_N and lithium oxynitrides (LiN_x_O_y_) species establishing a dense and uniform SEI film.^[^ ^169^ ^]^ Further, the SEI formed through the sustained release of LiNO_3_ studied through cryogenic‐electron microscopy (cryo‐EM) revealed a structural change of SEI from an amorphous to bilayer configuration (TEM image, Figure 11d) along with the existence of nitrogen‐containing species.^[^ ^170^ ^]^ Further, to promote the dissolution of LiNO_3_ in carbonate‐based electrolytes, carrier salts or solvents were introduced into the electrolyte solvent. Gao et al. reported a transesterification strategy through compounds 1,3‐dimethylimidazolium dimethyl phosphate (DIDP) and trimethylsilyl trifluoroacetate (TMSF) to produce dimethyl trimethylsilyl phosphate (DTMSP) and 1,3‐dimethylimidazolium trifluoroacetate (DITFA) in carbonate electrolyte. The transesterification generated DTMSP and DITFA additives carry multifunctional properties; dissolution of LiNO_3_ in carbonate electrolytes and scavenging HF and H_2_O in the electrolyte solution. The in situ generated DITFA through transesterification enabled the dissolution of LiNO_3_ in the carbonate solvent via the TFA^−^ anion and the combined role with DTMSP developed thin and robust interphases; CEI rich in P, N, and Si species and Li_3_N and Li_3_P rich SEI on the anode. Modified electrolyte (BE+PFN) exhibited a low Li nucleation overpotential (Figure 11e) and also demonstrated a high C.E of 98.3% (Figure 11f) among the blank, (BE+PN), and (BE+FN) formulations at 96.2%, 97.8%, and 97.2%, respectively.^[^ ^171^ ^]^ Similar to DITFA additive, amide‐based heptafluorobutyramide (HFT) was reported to promote the dissolution of LiNO_3_ in a carbonate electrolyte. Modified electrolyte LiPF_6_ in EC:DMC electrolyte containing (HFT+LiNO_3_) demonstrated a lower nucleation potential of about 14 mV than the blank LiPF_6_ in electrolyte (**Figure** **12**a,b). Ex situ SEM studies revealed a smooth Li deposition achieved using (HFT+LiNO_3_) electrolyte, which was due to the synergistic interaction of HFT and LiNO_3_ along with a favourable Li^+^ transport kinetics. Addition of HFT additive not only lowered the Li‐ion de‐solvation barrier due to an alteration in the solvent shell from solvent‐dominant to anion‐dominated structure, but also, they were reduced along with NO_3_^−^ anions developing an SEI rich in Li_3_N/LiF species. Modified carbonate electrolytes containing 0.5 wt.% HFT and 0.5% LiNO_3_ improved the C.E of Li ǀǀ Cu cells to about 97.1% and showed a good compatibility and capacity retention of about 100% for the LiN_0.33_Mn_0.33_Co_0.33_O_2_ and LiFePO_4_ cathodes. Further, the combination of additive improved the cyclability of LiFePO_4_ cathode even at a low temperature to room temperature cycling.^[^ ^172^ ^]^ Yan et al. employed copper fluoride as a dissolution promotor to dissolve LiNO_3_ in EC:DEC solvents. The CaF_2_‐based carbonate electrolyte tested with Li ǀǀ LiNi_0.80_Co_0.15_Al_0.05_O_2_ cell showed a 53% capacity retention after 300 cycles. Molecular dynamic simulation revealed a stronger interaction between Cu^2+^ and NO_3_^−^ as compared to Li^+^ and the competition of as‐formed Cu^2+^–NO_3_^−^ complex and copper ions facilitated the dissolution of LiNO_3_ in EC:DEC solvents.^[^ ^173^ ^]^ Tin‐based dissolution promoter Sn(OTf)_2_ was reported to improve the C.E of Li to 98.14% for over 150 cycles. Typically, the Lewis acidity of Sn^2+^ can effectively coordinate with NO_3_^−^ and favour dissociation of ion pairs rather than the decomposition of solvents. The dissociation mechanism that was observed with the CuF_2_ compound applies to the Sn(OTf)_2_ on the basis of higher charge difference of Sn^2+^ with the Li^+^ to attract NO_3_^−^ ions in the electrolyte solution. As a result, the solubility of LiNO_3_ in 1 M LiPF_6_ in EC:DEC electrolyte could be increased to about 5 wt.% in the presence of Sn(OTf)_2_ solvation promotor.^[^ ^174^ ^]^

The LiTFSI–sulfone‐based electrolyte reported for improving the CEI properties in LiNi_0.80_Mn_0.1_Co_0.1_O_2_ cathodes was also found to be effective in developing a stable SEI and improving C.E of Li metal. Sulfone‐based electrolyte formulated as 3.25 M LiTFSI–SL with LiNO_3_ enabled a high C.E of 99% for the Li metal and the Li‐ion cells with Li ǀǀ LiNi_0.8_Co_0.1_Mn_0.1_O_2_ exhibited a capacity retention of 99.5% at the end of 200 cycles. Electrochemical cycling even at an elevated temperature of 55 °C, the Li ǀǀ NMC811 cells delivered 60% of initial cycle capacity at the end of 200^th^ cycle. The solvation structure of modified HFE LiTFSI–SL electrolyte understood through the molecular dynamics simulation revealed that the electrolyte was solvated with 3.44 SL molecules and 1.03 TFSI^−^ anions, where the solvation sheaths are connected through SL (Figure 8g). With the addition of 0.1 M LiNO_3_, the concentration of TFSI^−^ anions increased around NO_3_^−^ and additional TFSI^−^ anions were connected to the solvation sheaths of Li ions. Owed to an increased concentration, the binding sites for Li‐ion hopping in the concentrated electrolyte 3.25 M LiTFSI–SL with HFE improved the Li‐ion transference.^[^ ^114^ ^]^ The stability of Li metal can also be improved by adjusting the solvation structure of electrolyte via an interaction between ions and solvents. Lithium trifluoroacetate (LiTFA) as an additive possessing a high donor number was introduced to regulate the solvation structure of carbonate‐based electrolyte. An anion‐rich solvation structure with a decreased solvent coordination capability was developed through a strong interaction between TFA^−^ and Li^+^ ions contributing to the formation of inorganic–organic interphase on Li metal. Under conditions of lean electrolyte and limited Li source (critical parameters for Li‐metal batteries), the developed Li metal interphase realized via LiTFA showed high Li plating/stripping efficiency of 99.4% and the half cells Li ǀǀ NMC811 showed a capacity retention of 87.37%.^[^ ^175^ ^]^ Wang et al. introduced an organic nitrate‐based additive, isosorbide nitrate (ISDN) to stabilize the Li‐metal anode. The ISDN additive showed good solubility and compatibility with the ester electrolyte due to its abundant organic segments; the structure of ISDN compared with LiNO_3_ is depicted in Figure 12c. It was found that the solubility of ISDN appeared to be acceptable until 1.5 M in an FEC:DMC solvent, whereas the concentration of LiNO_3_ could reach to about 0.3 M under similar solvent environment. Modified ISDN‐based carbonate electrolyte showed an improvement in the Li plating/stripping behavior and performances; the Li symmetric cells underwent a minimal polarization of 75 mV at 1 mA cm^−2^ (Figure 12d) and a good C.E of 98.5% for the Li ǀǀ Cu cells at the end of 200 cycles. Further, the Li metal coupled with NMC532 tested with ISDN‐based carbonate electrolyte retained about 80% of the capacity with C.E of 99.5% at 0.4 C rate. It was noted that the decomposition of ISDN leaves out nitrogen containing species like LiN_x_O_y_ as also observed with LiNO_3_ which establishes a stable SEI and smooth Li deposition over several cycles.^[^ ^176^ ^]^

The depletion of Li is obvious over an increase in the cycles; those lost Li are termed as “dead Li”. Functional additives were also effective in the above case where the source of Li is limited to a single electrode in battery chemistries such as Cu ǀǀ Li anode‐less batteries. Lithium iodide (LiI) was introduced as an additive to suppress dendritic growth and reactivate the dead Li. Even the addition of LiI in traces about 50 mM to LiTFSI in DOL:DME with 2 wt.% LiNO_3_ ether electrolytes controlled the loss of dead Li. Mechanistically, the polyiodide and I_2_ migrate and react with the Li anode forming iodide as a product, which can subsequently return back to the cathode and gets oxidized as polyiodide or iodine. Interestingly, this shuttle reaction of polyimide/I_2_ does not produce any electrons or capacity. The Cu ǀǀ LiFePO_4_ cells based on LiI demonstrated 72% and 42% capacity retention after 100 and 200 cycles, respectively. Since, the LiI is known to promote self‐discharge in batteries, the concentration of LiI is critical. Self‐discharge tests with Li‐ion cells using 25 mM of LiI showed improved capacity retention from 88.4% to 76.7% after resting time of 24 h to 72 h along with capacity recoveries exceeding 100%.^[^ ^177^ ^]^ Phosphor‐based additive, alkyl‐triphenyl‐phosphonium bromide was reported to improve the stability of Li metal anode. Molecular dynamic simulations revealed that the presence of phosphonium bromide likely altered the coordination environment. It was noted that the interaction of Li^+^ with solvent molecules are avoided through a strong coordination between Li^+^ and Br^−^ resulting in a firm alkyl‐TPP cation coordination with EC and DMC molecules. The downplay of alkyl‐TPP cation and Br^−^ anion suppress the dendritic growth and stabilize the Li metal interphase. The Li ǀǀ Li_4_Ti_5_O_12_ cells employing amyl‐triphenyl based TTP additive containing LiPF_6_‐EC:DMC electrolyte showed good cyclability over 1000 cycles.^[^ ^178^ ^]^ Similarly, 4,6‐dimethyl‐2‐mercaptopyrimidine (DMP) as an electrolyte additive influenced the Li‐ion solvation structure and reside in the inner Helmholtz plane of Li anode. As a result, the DMP absorbed on the Li metal surface can effectively suppress the parasitic reactions and assist in modifying the Li deposition behavior. The symmetric Li cells constructed with electrolyte LiTFSI in DOL:DME containing DMP additive demonstrated cycling stability up to 800 h at a current density of 3 mA cm^−2^ and Li ǀǀ Cu cells exhibited a C.E of 98% at a current density 1 mA cm^−2^.^[^ ^179^ ^]^

Metalloid tellurium (Te) based lithium tritelluride (LiTe_3_) was reported as an electrolyte additive for anode‐less Li‐metal (Li‐S) batteries. Polysulfides and associated shuttling between Li anode and S cathodes brings about several complexities involving loss of active sulfur and Li, capacity fade, and self‐discharge, etc. It is likely that the existence of LiTe_3_ additive reacted with those polysulfides and serve as a redox mediator to accelerate the sulfur kinetics and its utilization. Anode‐less Ni ǀǀ Li_2_S cells infiltrated with an electrolyte LiTFSI in DOL:DME+0.1 M LiTe_3_ presented a good cyclic stability up to 100 cycles with a capacity of about 340 mAh g^−1^ at a current rate of 0.5 C. The altered surface chemistry of anode and cathode materials and the existence of Li_2_TeS_3_/Li_2_Te‐enriched interphase on the anode enabled the anode‐less Li‐S batteries to undergo better cyclic performances.^[^ ^180^ ^]^ The interfacial stability is one among the essential factor to realize improved performance and cyclability of Li metal anode. Alongside, the mechanical strength of Li, especially when considering negative‐to‐positive (N/P) ratio, thin Li strips with desired strength and interfacial stability are preferred. Huang et al. adopted a zinc dialkyldithiophosphate (ZDDP) as an additive which presented anti‐pressure and anti‐wear properties to engineer the Li metal surface. An in situ tribological reaction between the ZDDP additive and Li metal during the mechanical rolling established an organic/inorganic interface ≈450 nm as illustrated in Figure 12e. The reformed Li metal via ZDDP offered a high mechanical strength with a hardness of 0.84 GPa and Young's modulus 25.90 GPa. The processed thin Li anode (5 to 50 µm) demonstrated an extended cycle life of 1700 cycles at a high current density of 18 mA cm^−2^ using ether‐based LiTFSI in DOL:DME with 2% LiNO_3_ electrolyte. Further, the Li‐metal battery Li ǀǀ LiFePO_4_ containing carbonate‐based LiPF_6_ in EC:EMC:FEC electrolyte exhibited an extended cyclability for over 350 cycles (Figure 12f) and rate capability with a capacity retention of 82%. Evidence fetched through the XPS and Extended X‐ray Absorption Fine Structure (EXAFS) revealed that the Zn–S bonds of ZDDP that were decomposed during the mechanical processing formed Zn–O bonds (Figure 12e), which further takes the form of zinc polyphosphates providing good mechanical properties to the thin Li strips. Thus, the lithiophilic and mechanically strong artificial SEI developed using ZDDP additive significantly suppressed the dendrite growth and interfacial damage to afford a uniform Li plating, extended cycle life, and improved performances.^[^ ^181^ ^]^

Lithium metal is regarded as a “holy grail” of battery technologies due to an exceptionally high energy density. Concerns that arise upon cycling Li metal such as in‐homogeneous ionic flux, uneven deposition, rough morphology, dendritic growth, low Coulombic efficiency, etc., were addressed to a certain extent via functional additives. Lithium nitrate as an additive (0.5–3 wt.%) demonstrated a substantial improvement in the cyclability of Li–metal batteries by forming uniform SEI rich in inorganic species. Fluorinated additives such as FEC, fluorophosphate, fluorobutyramide, trifluoroacetate, etc., showed strong film‐forming capabilities on Li metal anodes. Electrolyte containing combinations of additives are interesting in terms of multiple roles and practicality, electrolyte mixtures employing LiNO_3_ as primary additive with any of the secondary additives like LiDFP, HFT, LiI, etc., in lower concentrations aided the formation of SEI rich in LiF and fluorine‐based species. Since the ether‐based electrolytes and Li metal show good compatibility, the role of additives in the carbonate‐based electrolytes for improving the Li metal interface and its associated properties are comparatively expected to be more. For example, the solubility discrepancy shown by LiNO_3_ in an ester‐based electrolyte promptly exposes the challenges in adopting similar solutions. Additives like DITFA, HFT, Sn(OTf)_2_, and CaF_2_ promoted the solubility of LiNO_3_, where the Lewis acidity of cations coordinated with NO_3_^−^ favored the dissociation mechanism. In spite of such advancements, an in‐depth understanding of the physical, chemical, mechanical, and electrochemical properties of the developed protection layer on Li metal requires the use of advanced characterization techniques. Owed to the complex organic‐inorganic structure of passivating layer, it is quite challenging to study the properties, cryo‐EM is one of the analytical techniques exploited to visualize the physicochemical properties of the surface layer at nano level.

The interfacial properties of both the anode and cathode material certainly influence the cell performance like capacity retention, rate capability, and cycle‐life. The above sections exclusively focussed on the interfacial phenomena; solid‐electrolyte, cathode‐electrolyte, and Li metal interphases and their properties established in the presence of functional electrolyte, associated mechanism, and their performance improvement. In this section, the additives that were reported to improve the capacity retention are discussed, while a thin‐line cuts‐off the discussion between the interface and the capacity retention.

**Figure 13:**
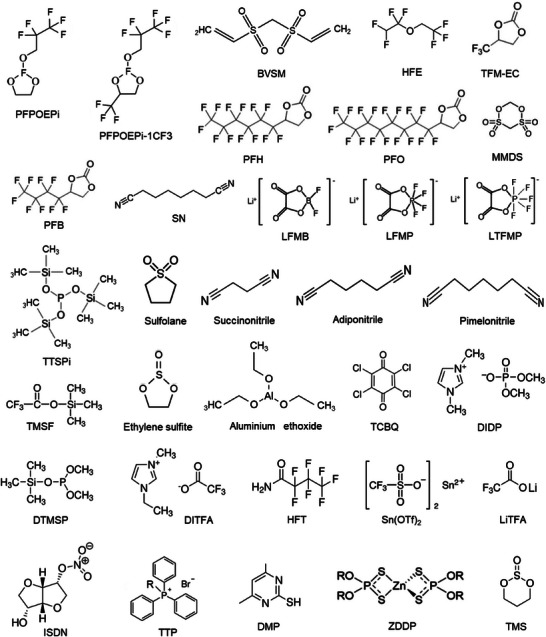
Chemical structures of functional additives adopted for interfacial restructuring and metal passivation in Li–metal batteries.

The film forming additive FEC is reported to show diverse film forming ability on graphite anode and LiMn_2_O_4_ cathode at a high temperature. The SEI formed on the graphite anode assisted by FEC additive exhibited lower interfacial resistance, good rate capability, thermal stability, and enhanced cycle retention. In contrary, a thick layer and higher interfacial resistance on LiMn_2_O_4_ cathode were observed, which caused poor electrochemical performance. It was concluded that the amount of additive is essential when formulating the liquid electrolyte for full cell battery chemistries.^[^ ^182^ ^]^ The effect of cyclic sulfate as additives that include ethylene sulfate (1,3,2‐dioxathiolane‐2,2‐dioxide (DTD)), trimethylene sulfate (1,3,2‐dioxathiane 2,2‐dioxide, (TMS)), and propylene sulfate (4‐methyl‐1,3,2‐dioxathiolane‐2,2‐dioxide, (PLS) were investigated on graphite ǀǀ NMC111 cells. The sulfate‐based additives in combination with 2% VC showed a decrease in the cell resistance and improved C.E with the TMS and DTD additives, in contrary the PLS does not show tendency of an additive.^[^ ^183^ ^]^ Triphenyl borate (TPB) was proposed as a film forming additive to enhance the cycling stability of Li‐rich Li_1.2_Mn_0.54_Ni_0.13_Co_0.13_O_2_ cathode. The capacity fade associated with Li‐rich oxides arises from the electrolyte decomposition and irreversible phase transformation from layered‐to‐spinel and Ni metal dissolution. It is likely that the 3% TPB containing LiPF_6_ in EC:EMC:DEC electrolyte improved the cycling stability of Li ǀǀ Li_1.2_Mn_0.54_Ni_0.13_Co_0.13_O_2_ cells with a highest capacity retention of 78% among 1, 2, and 4 wt.% TPB containing electrolytes at 55%, 59%, and 76% retention, respectively. The improved cycling stability of 3 wt.% of TPB additive‐based electrolyte arose from the improved stability to the electrochemically derived spinel phase, which further restricted the transformation from layered‐to‐spinel structure. Further, the onset decomposition of TPB at around 3.8 V supressed the oxidation of carbonates and the generation of HF triggering the metal dissolution.^[^ ^184^ ^]^ Chen et al. studied the effect of methylboronic acid ester on the cycling properties and stability of high voltage cathodes. The carbonate electrolyte LiPF_6_ in EC:DMC with 1 wt.% boronic ester additive improved the capacity retention of LiNi_0.5_Mn_1.5_O_4_ (LNMO) cathodes to about 96% at 40 °C. It was reported that the boronic ester influenced the solvation structure via B–N and B–O–C = O groups and reaction kinetics of electrolyte solvent. Further evidence through XANES denoted that the site symmetry and coordination geometry of LNMO cathode in an electrolyte was more stabilized in the presence of boronic ester and lead to formation of a smooth and thin CEI film.^[^ ^185^ ^]^ The chemical structure of the functional additives developed for metal passivation and SEI development are depicted in **Figure** **13**.

The capacity retention and cycle life are related to the several properties of interfacial film (strength, ionic conductivity, robust, etc.). Thus, maintaining a robust and stable interface through the functional additives suffices the retention of near to‐pristine nature of active material contributing to enhanced electrochemical performances. Functional additives positively influenced one or more among the properties of interphases on either anode or cathode, in certain cases both the electrode's interface offering improved electrochemical performances. Over an increase in the cycles, retaining all the components in their active and pristine state is quite challenging. Thus, adopting collective approaches like partial substitution of metal ions and inorganic nano‐coating over the active material combined with the functional additives may perhaps benefit the cathode particles to refrain from structural collapse during the cycling.

### 4.2 High‐Voltage Additives

**Figure 14:**
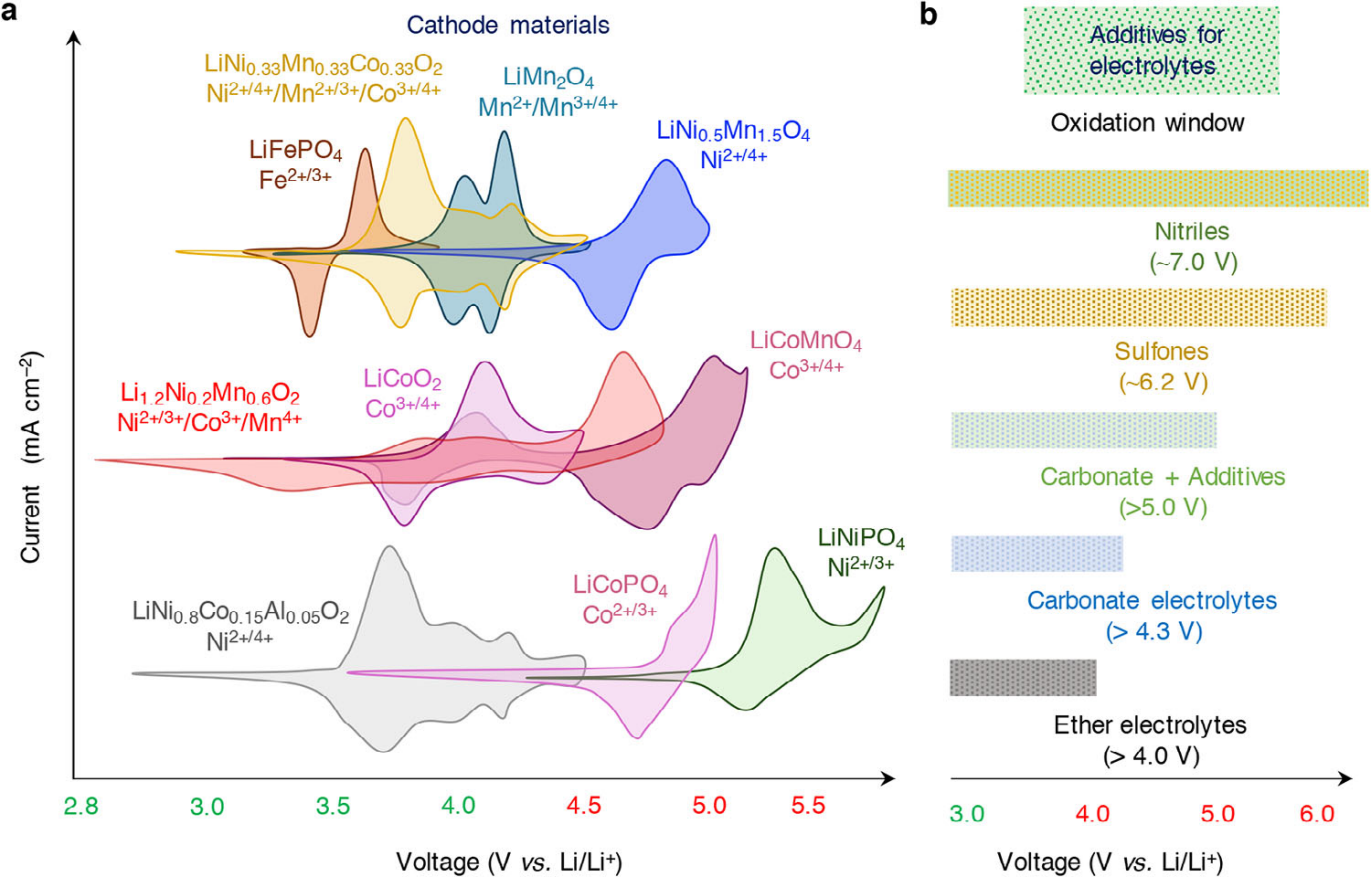
a) The redox potentials of cathode materials employed in commercial Li‐ion batteries and next‐generation high‐voltage cathode materials for high energy density Li‐based batteries. The cyclic voltammograms depicting the redox potentials are characteristic of the cathode materials, while the current represented are for understanding the redox potentials and must not be scaled. b) Electrochemical stability window of electrolytes and various additives to increase the voltage limit of electrolytes capable of achieving electrochemical cycling of high‐voltage cathode materials.

Cathode materials possessing a high voltage and specific capacity could further drive the energy densities of Li‐ion batteries. Several cathode materials are capable of operating at upper cut‐off voltage ranging 4.2 to 5.0 V (versus Li/Li^+^) which include lithium cobalt oxide (LiCoO_2_ V = 4.5 V), lithium manganese oxide (LiMn_2_O_4_ V = 4.5 V), lithium nickel manganese oxide (LiNi_0.5_Mn_1.5_O_4_ V = 4.5–5.0 V), lithium nickel manganese cobalt oxide (LiNi_x_Mn_y_Co_z_O_2_ V = 4.3–4.7 V), lithium‐rich nickel manganese cobalt oxide (LR‐NMC V = 4.6–4.8 V) and LiMPO_4_ (M = Ni and Co V = 4.2–5.8 V). The electrochemical redox properties of high‐voltage cathode materials and the additives that enhance the voltage window of electrolytes are portrayed in **Figure** **14**. It is obvious that an increase in the upper cut‐off voltage exceeding 4.3 V versus Li/Li^+^ positions the cathode material and electrolyte solution under a risk of continuous oxidation of electrolyte solution leading to severe capacity fade.^[^ ^186^, ^187^ ^]^ Such conditions deteriorate the electrode and electrolyte components and further risks the safety of battery operation. From the electrolyte viewpoint, designing an electrolyte solution like high‐concentration, localized high‐concentration, additives, etc., are some of the stimulating strategies investigated to rise the voltage window of electrolytes beyond its expectations.^[^ ^188^, ^189^, ^190^ ^]^ In such an electrochemical setup, realization of a stable structure, passivation layer/film over the cathode, etc., are necessary for the cathode materials to remain stable at a high voltage limit.

**Figure 15:**
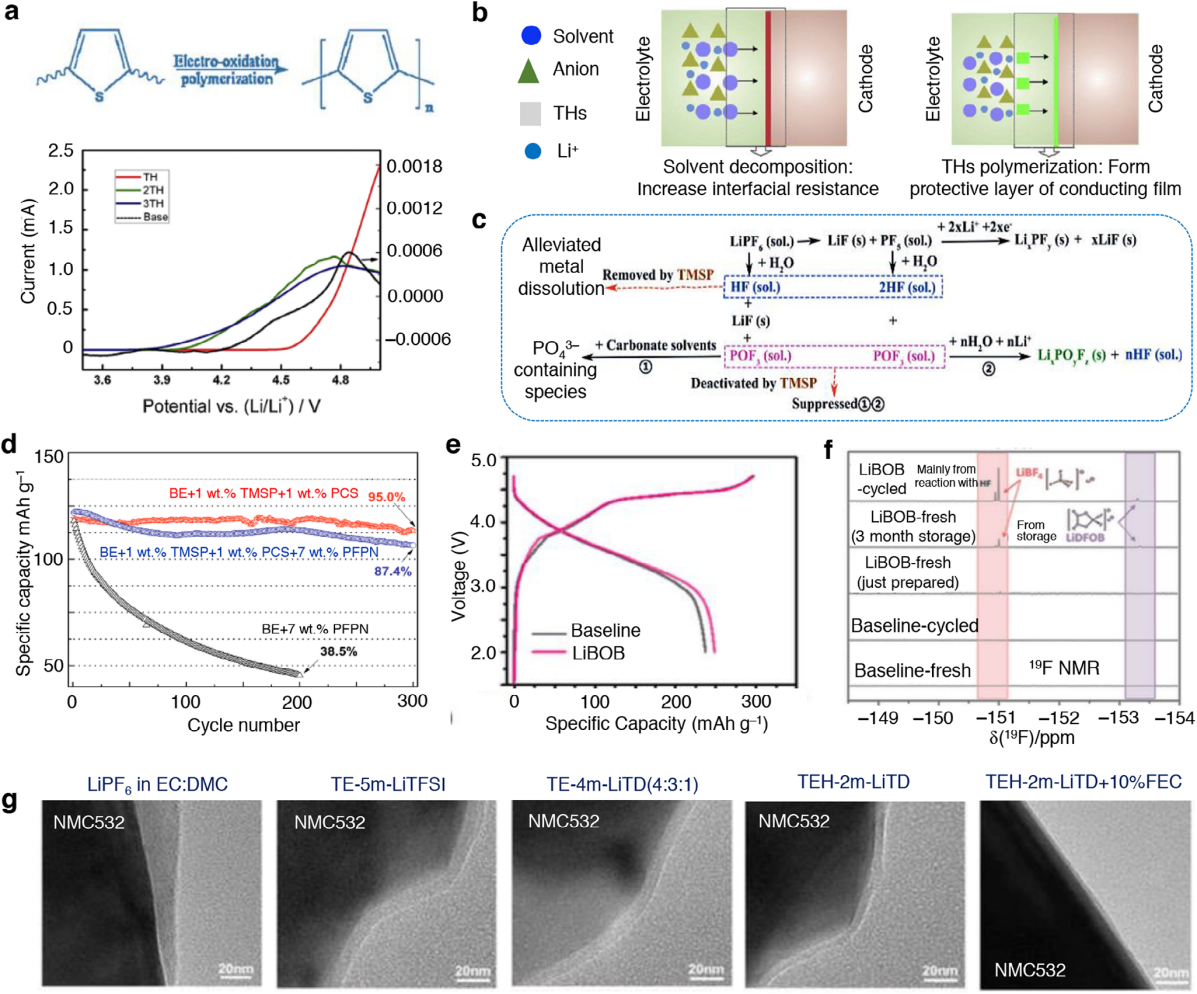
a) Polymerization reaction of thiols and chronoamperometry studies depicting the electrochemical stability of thiols. b) Schematics of Li‐ion battery demonstrating the interface construction of THs compared with blank electrolyte. Reproduced with permission.^[^ ^191^ ^]^ Copyright 2015, Elsevier. c) The possible reaction mechanism and functions of TMSP and PCS additives in graphite ǀǀ LiNi_0.5_Mn_1.5_O_4_ cells. d) Respective cycling stability obtained at 1 C rate at a cut‐off voltage 3.5–4.9 V. Reproduced with permission.^[^ ^194^ ^]^ Copyright 2017, Wiley‐VCH. e) Respective electrolyte saturated graphite ǀǀ Li‐rich cathode full‐cell's charge‐discharge profiles. f) ^19^F NMR spectra for the LiBOB additive‐based electrolyte compared with baseline LiPF_6_ in EC:DMC electrolyte in fresh, stored, and cycled one. Reproduced with permission.^[^ ^196^ ^]^ Copyright 2022, Wiley‐VCH. g) TEM images of cycled NMC532 electrodes depicting the developed interphase in the presence of various electrolyte formulations. Reproduced with permission.^[^ ^199^ ^]^ Copyright 2021, Wiley‐VCH.

Thiophene derivatives that includes 2,2′‐bithiophene (2TH) and 2,2′,5,2′'‐terthiphene (3TH) were reported as an additive for high‐voltage operation of LiCoO_2_ cathode (**Figure** **15**). The oligomers of 2TH and 3TH possessed a lower oxidation potential and as a fact they decompose much earlier than the electrolyte solvent establishing a polymeric conductive coating layer over the LiCoO_2_ particles. A rational comparison of the TH's function as an additive with the blank carbonate electrolyte are portrayed in Figure 15b, where the onset decomposition protected the LiCoO_2_ particles at higher voltage limit. The stable polymeric film formed through the electro‐oxidation restricted further decomposition of solvent and salt at a high voltage. Figure 15a display the linear square voltammetry curves, where an increase in the number of TH unit reduced the anodic stability of the additive. The electrolyte containing 0.1 wt.% 3TH enabled the LiCoO_2_ cathode to achieve a capacity retention of 84.8% at a high cut‐off voltage 4.4 V.^[^ ^191^ ^]^ The polythiophene derived through the electrochemical oxidation of thiophenes created a homogeneous coating layer that imparted a high conductivity and good chemical stability to the electrode.^[^ ^192^ ^]^ The effect of diphenyl diselenide (DPDS) was studied by Park et al. as an additive for high voltage operation of LiCoO_2_ cathode. It was deduced through DFT studies that the DPDS additive possessed a higher HOMO level due to an electron‐donating group and lower LUMO energy levels than the EC and DEC electrolyte solvents. The modified electrolyte containing 0.1% DPDS showed a capacity retention of 95.2% in the graphite ǀǀ LiCoO_2_ cells, whereas the additive‐free cells reported about 88.7% retention. It was found through the ex situ X‐ray diffraction (XRD) studies that the DPDS additive preserved the layered structure of graphite, in contrary the additive‐free electrolyte succumbed to the erosion of graphite layers with repetitive cycles.^[^ ^193^ ^]^

A combination of additives succinic anhydride (SA) and 1,3‐propane sultone containing electrolyte 1 M LiPF_6_–EC/EMC (1:2, v/v) boosted high‐voltage performance. The graphite ǀǀ LiNi_0.5_Mn_1.5_O_4_ (LNMO) cells infiltrated with SA (2 wt.%) + PS (3 wt.%) containing 1 M LiPF_6_–EC/EMC electrolyte showed better electrochemical performances than the individual SA (2 wt.%) and PS (3 wt.%) additive containing 1 M LiPF_6_–EC/EMC electrolyte. The improved cyclability of carbonate‐based electrolyte with SA and PS additive was due to the formation of stable SEI film over the graphite anode and their oxidation stability at high voltage.^[^ ^74^ ^]^ Xu et al. reported tris(trimethylsilyl) phosphite (TMSP) and 1,3‐propanediolcyclic sulfate (PCS) as a binary functional additive for high voltage LiNi_0.5_Mn_1.5_O_4_ cathode. The combination of binary additives decomposes and involve in the modification of interfacial films on both the anode (SEI) and cathode (CEI) electrodes. Likely, the DFT simulations and ex situ XPS results validated that the PCS undergo reduction at the MCMB anode forming sulfite species, some of which migrate to the cathode and oxidize to Li_2_SO_4_ and alkyl sulfides (C–S–S–C) species. The modified interfacial films are conductive and resistant toward oxidative/reductive decomposition at a high voltage and elevated temperature. The mode of TMSP action controlling the irreversible species and HF is depicted in Figure 15c. The Li‐ion battery MCMB ǀǀ LiNi_0.5_Mn_1.5_O_4_ tested at room temperature (Figure 15d) and 50 °C exhibited a capacity retention of 95.0% and 79.5% at 1 C rate and showed cyclability until 300 and 200 cycles, respectively.^[^ ^194^ ^]^ Liao et al. developed and reported an orthochelated borate salt, lithium bis(monofluoromalonate)borate (LiBFMB) for high voltage LiNi_0.5_Mn_1.5_O_4_ cathode material. The presence of fluorine in the borate anion of LiBFMB involved in rising the oxidation potential and promoted the ion dissociation. Half cells based on LiNi_0.5_Mn_1.5_O_4_ cathode saturated with individual LiBOB and LiPF_6_ based EC:DMC:DEC electrolyte experienced a lower resistance (*R*_total_) 135.1 Ω and 437.3 Ω, respectively than the LiBFMB added electrolyte cells at 1028 Ω. A better capacity retention was observed with salts LiBOB and LiPF_6_ than the LiBFMB cells. Interfacial chemistry of CEI formed on LiNi_0.5_Mn_1.5_O_4_ cathode in the presence of individual boron‐based additives in comparison to LiPF_6_ salt showed a significant difference in the thickness of interfacial film, which followed a decrease from LiBFMB to LiBOB and LiPF_6_. Interpretation of XPS results in‐line with the cyclic voltammogram studies found that the LiBFMB and LiBOB remained stable without/or a minimal decomposition at high potentials (4.9 V). It is known that the choice of solvent being crucial for the SEI's physical, mechanical, and compositional properties, using PC as a sole solvent with LiBFMB demonstrated a comparatively lower resistance than the EC:DMC:DEC electrolyte. A combination of high anodic stability, good solute solubility, and minimal cell impedance accounted for reasonable C.E of 97% after 30 cycles.^[^ ^195^ ^]^ Li et al. studied the effect of LiBOB additive to understand the capacity decay and voltage fade in Li‐rich layered oxide cathodes. The carbonate‐based electrolyte formulated with LiBOB salt enhanced the Li‐rich cathode's performance at a high‐voltage 4.5 V: a specific capacity of 248 mAh g^−1^ (Figure 15e) without a significant capacity fade until 70 cycles and a capacity retention of 95.5% after 150 cycles. It was found that the Li‐rich cathode underwent a minimal phase transformation and a uniform interphase in the presence of LiBOB based electrolyte. Further, the formation of B–F species validated through ^19^F NMR shown in Figure 15f certainly benefitted the Li‐rich cathode by scavenging the HF in an electrolyte. As a result, the corrosion of cathode surface, metal dissolution, and redeposition on the graphite were largely avoided.^[^ ^196^ ^]^ Similarly, the C.E and cyclability of Li‐rich Li_1.2_Ni_0.2_Mn_0.6_O_2_, (Ni/Mn = 1/3) cathode were improved through the addition of about 2% LiBOB as an additive in LiPF_6_ based electrolyte. The synergy of Li‐salts, LiBOB and LiPF_6_ promoted a sustained release of lithium difluoro(oxalate)borate (LiDFOB) anion, which efficiently scavenged the superoxo radical and HF in the electrolyte solution. Thus, the CEI developed in the presence of dual‐salts with BOB‐derived polymeric B–F/B_x_O_y_ enhanced the cycling stability and 92.5% capacity retention.^[^ ^197^ ^]^ A solvent depleted Li‐ion solvation sheath electrolyte was developed by sieving the additional solvent through Cu‐BTC (C_18_H_6_Cu_3_O_12_) MOF. It was noted that the modified electrolyte composed of crowned solvent‐depleted dominant contact‐ion pair without any free solvent molecules. A large electrochemical window, enlarged from 4.5 V to 5.4 V, stable CEI film, and good CE were reported with NMC811 and LiCoMnO_4_ cathodes and Li metal anode.^[^ ^198^ ^]^

**Figure 16:**
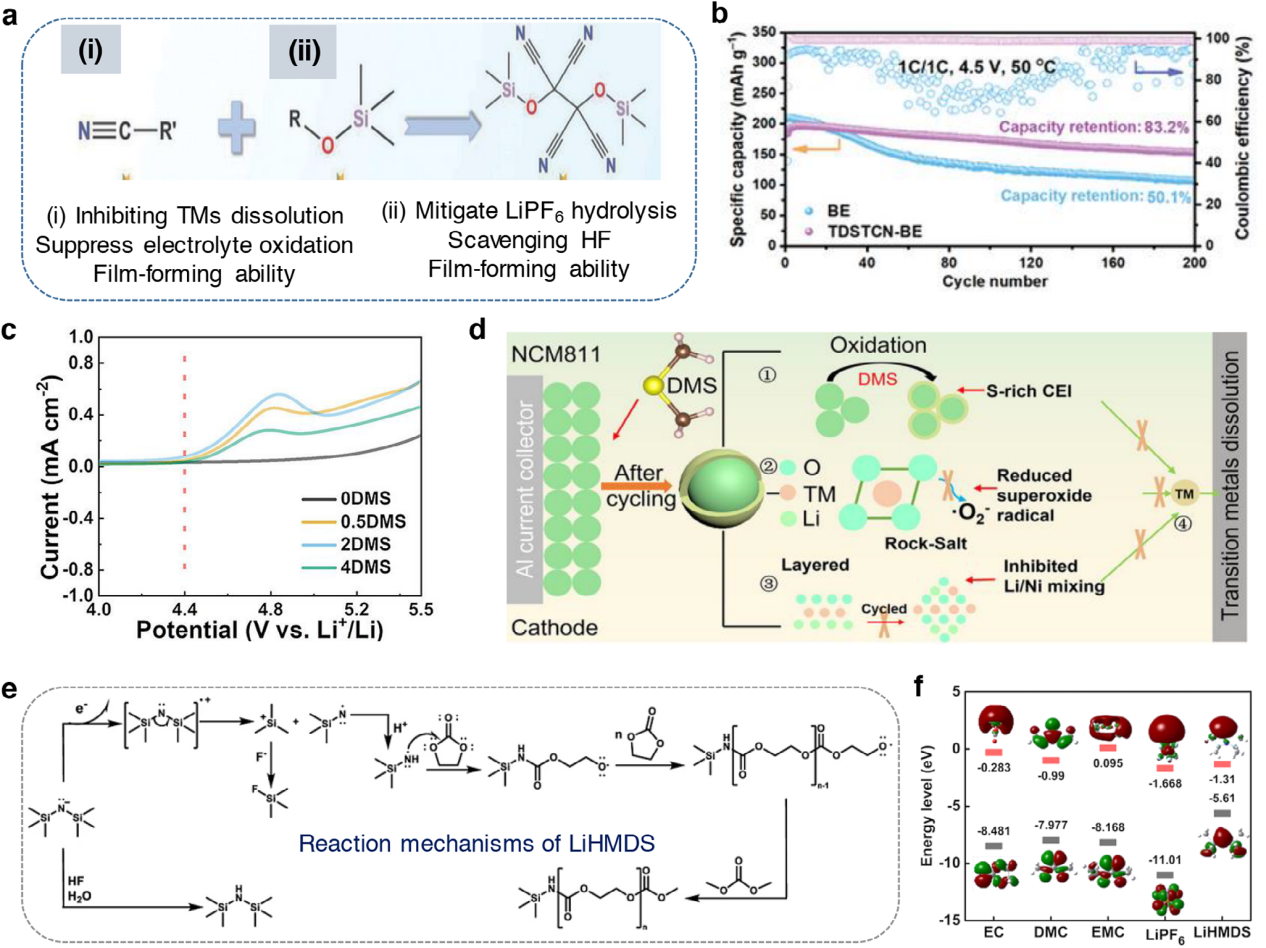
a) Schematics depicting the design of TDSTCN additive and its multifunctional properties. b) Cycling stability of graphite ǀǀ NMC cells tested at 1 C, high‐temperature of 50 °C with a cut‐off voltage 4.5 V. Reproduced with permission.^[^ ^200^ ^]^ Copyright 2023, Wiley‐VCH. c) Linear sweep voltammetry curves shown for various electrolyte formulations without DMS to 4% DMS additive. d) Schematic illustrating the roles of DMS; formation of sulfur‐rich SEI, deactivate superoxide radical, and mitigating cation mixing and metal dissolution. Reproduced with permission.^[^ ^202^ ^]^ Copyright 2024, American Chemical Society. e) Reaction mechanism of LiHMDS with two‐stages; preferential oxidation on NMC811 surface and scavenge HF and H_2_O in the electrolyte. f) Energy level diagram, HOMO and LUMO energy of LiHMDS compared with the conventional LiPF_6_ salt and EC, DMC, and EMC solvents. Reproduced with permission.^[^ ^203^ ^]^ Copyright 2022 The Authors, Springer Nature.

Lin et al. employed dual‐salt strategy containing Li salts, LiTFSI and LiDFOB to alter the concentration of electrolyte capable of operating at a high cut‐off voltage under the conditions of low and high temperature. Electrolyte formulated with TMS, ethyl acetate, and FEC under complex proportions enabled the NMC532 cathode to remain stable at a cut‐off voltage 4.6 V at wide temperature range 25 °C to –80 °C. The Ni‐rich cathodes tested at a 4.6 V retained about 89% of its capacity after 200 cycles at 1 C rate, while at a low temperature –40 °C about 50% of the capacity could be recovered. The TEM images displayed in Figure 15g for the cycled NMC532 cathode at various electrolyte denoted that the TEH‐2m‐LiTD+10% FEC developed a smooth and thinner CEI about 5 nm than the dual‐salt HFE into TE‐4m‐LiTD and other electrolyte formulations. It was noted that an increase in the electrolyte concentration, the Li‐ion coordination with the solvent reduced, whereas the localized high concentrated electrolyte containing TMS, FEC, and FEC approached nearly zero denoting the role of a hydrofluoroether diluent.^[^ ^199^ ^]^ High voltage and high‐temperature cyclability of Ni‐rich cathodes were reported by Ren et al. via cyano‐siloxane based multifunctional additive, 2,2,7,7‐tetramethyl‐3,6‐dioxa‐2,7‐disilaocytane‐4,4,5,5,‐tetracarbonnitrile (TDSTCN). **Figure** **16**a illustrate the chemical structure and notable attributes of TDSTCN on the properties of NMC cathode. The addition of 0.5 wt.% TDSTCN to a carbonate‐based electrolyte enabled graphite ǀǀ LiNi_0.9_Mn_0.05_Co_0.05_O_2_ full‐cell to realize an increased cycle life of 800 cycles and capacity retention of 81.2% at room temperature. Alongside, a good performance improvement was notable at 50 °C with a maximum cut‐off voltage 4.5 V (Figure 16b) achieving a capacity retention of 83.2% and cyclability up to 200 cycles. The synergistic effect of –CN and Si–O groups of TDSTCN were effective toward HF scavenging in the electrolyte and inhibited the dissolution of transition metal, which likely promoted for the development of a stable and robust interfacial layer on both the Ni‐rich and graphite electrodes.^[^ ^200^ ^]^ Chen et al. reported a borate‐functionalized disiloxane compound, 1,1,1,3,3‐pentamethyl‐3‐(3‐(4,4,5,5‐tetramethyl‐1,3,2‐dioxaborolan‐2‐yl)propyl) disiloxane (PMBPDS) as an additive for safe operation of high voltage Ni‐rich cathodes. An improved cyclability and capacity retention of Ni‐rich cathodes were observed even at a low concentration, about 0.2 wt.% PMBPDS formulated LiPF_6_ in EC:EMC:DMC electrolyte employed graphite ǀǀ LiNi_0.8_Mn_0.1_Co_0.1_O_2_ cells exhibited a higher capacity retention of 70.6% after 200 cycles at a cut‐off voltage 4.5 V. The metal dissolution in the electrolyte solution, especially the content of Ni in the electrolyte solution was reduced to about six times to 0.381 mg L^−1^ from 2.833 mg L^−1^ when compared with additive‐free electrolyte. The possible control of metal dissolution, minimal cell impedance and an improved cyclability up to 4.5 V were anticipated due to construction of a stable CEI in the presence of PMBPDS additive contributed through the polymerization products disiloxane moiety and dioxaborolane.^[^ ^201^ ^]^ Dimethyl sulphide (DMS), a thioether additive was tested for high‐voltage operation of Ni‐rich cathodes. The linear square voltammetry curve depicted in Figure 16c revealed the oxidation at 4.4 V for the DMS‐containing electrolyte, which denotes the prior oxidation of DMS than the solvents to establish a stable protective layer. Lithium‐ion half‐cell Li ǀǀ NMC811 and full cell graphite ǀǀ NMC811 in the presence of DMS exhibited a long term cyclability with a capacity retention of about 85% (cut‐off voltage 4.6 V) and 75% (cut‐off voltage 4.4 V), respectively. The schematics illustrated in Figure 16d details the course of DMS action and its positive attributes on the NMC cathodes. Typically, the DMS additive through its electron‐donating group targeted and deactivated the superoxide radicals evolved from the NMC811 cathodes at a high‐voltage cycling. This significantly resulted in a stabile inorganic‐rich CEI and contributions from sulfur‐rich species offered structural stability and enhanced cyclability to NMC811 cathodes.^[^ ^202^ ^]^ Lithium hexamethyldisilazide (LiHMDS) as an additive formulated with fluorine‐based non‐aqueous electrolyte showed an improved cyclability of NMC cathodes at 4.5 V. The LiHMDS preferably oxidized before the solvent forming radical anions which captured the protons of carbonate solvents and polymerized to form a stable CEI layer as portrayed in Figure 16e. Understanding through energy level diagram (Figure 16f) revealed a high HOMO energy of –5.61 eV than the conventional salt and solvents. Alongside, the intrinsic organic base nature of LiHMDS and strong binding energy of HMDS^−^ and H_2_O and HF enabled quick capture and construction of uniform, thin, and robust CEI on NMC cathodes. Even a low amount LiHMDS additive about 0.6 wt.% enabled improved cyclability to the NMC811 cells at 4.5 V for over 1000 and 500 cycles at 25 °C and 60 °C, respectively.^[^ ^203^ ^]^

**Figure 17:**
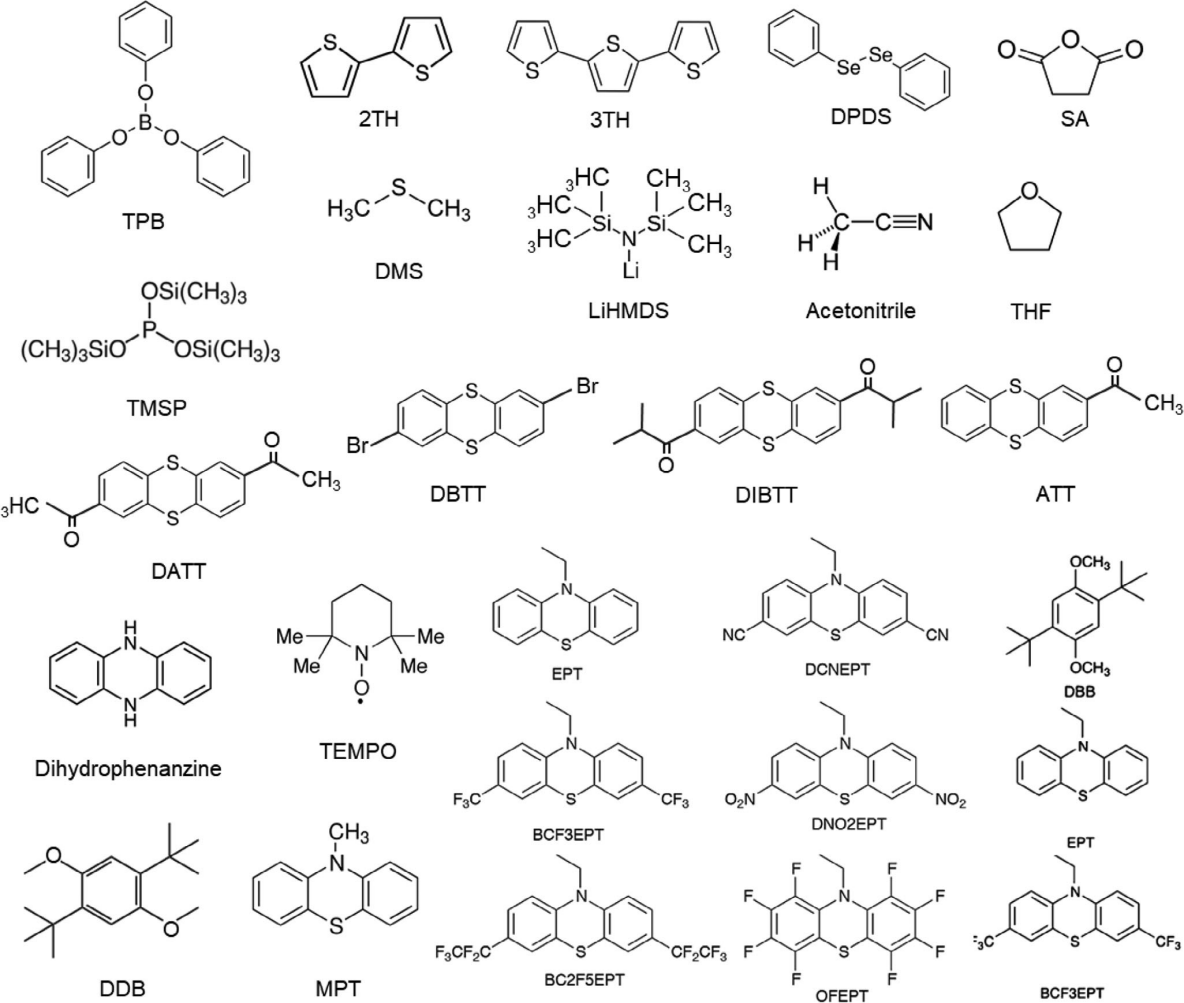
Chemical structures of functional additives employed for capacity retention and high‐voltage operation of Li‐based batteries.

Sulfone‐based electrolyte containing LiTFSI and TMS incorporated FEC was reported to improve the properties of high voltage cathode and Li metal anode. Typically, the high oxidation stability of sulfone at the cathode and film‐forming ability of FEC on Li metal altered their interface amenable for both the electrodes. The interface developed in the presence of sulfone composed of fluorine and sulfur‐rich species, LiSO_2_F which effectively mitigated the metal dissolution and assisted uniform Li deposition. Sulfone‐based electrolyte infiltrated Li ǀǀ NMC811 battery demonstrated a high C.E of 99.3% and a capacity retention of 86.1%.^[^ ^204^ ^]^ Moon et al. designed a concentrated nitrile electrolytes LiTFSI in succinonitrile/acetonitrile (SN/AN) containing LiNO_3_ and InF_3_ as co‐additives. The solvent mixture SN/AN offered high oxidation and thermal stability to the Li metal anode. Nitrile‐based electrolyte containing (LiNO_3_+InF_3_) additives demonstrated stable cycling for Li metal coupled with either LiNi_0.33_Mn_0.33_Co_0.33_O_2_ or 5 V class LiNi_0.5_Mn_1.5_O_4_ cathode materials. The symmetric Li cells exhibited sable Li plating/stripping for over 2500 h at a current density of 0.1 mA cm^−2^. Lithium metal batteries constructed using LiNi_0.33_Mn_0.33_Co_0.33_O_2_ and LiNi_0.5_Mn_1.5_O_4_ cathodes demonstrated a capacity retention of 73% after 200 cycles and 90.4% after 100 cycles, respectively.^[^ ^205^ ^]^ Ether‐based electrolytes in general possess high ionic conductivity and are promising electrolytes for Li–metal batteries.^[^ ^206^, ^207^ ^]^ Wang et al. used data‐driven ML to predict additives for anode and cathode electrodes amenable for high voltage LiNi_0.5_Mn_1.5_O_4_ cathodes. About 28 additives refined through the performance metrics like area‐specific impedance, impedance rise, and final specific capacity were used for ML predictions. The electrolyte formulation containing binary additives LiBOB and SA with a baseline electrolyte 1 M LiPF_6_ in EC/EMC (1:9) identified through ML model demonstrated high specific capacity of 95 mAh g^−1^ among the predicted samples.^[^ ^208^ ^]^ In spite, the poor oxidation stability of ether electrolytes < 4 V versus Li/Li^+^ are a concern, especially when a Li metal as an anode is matched with high‐voltage cathode materials like LiNi_0.33_Mn_0.33_Co_0.33_O_2_, LiNi_0.5_Mn_1.5_O_4_, etc. Jiang et al. showed that an optimized combination of LiNO_3_ salt and VC solvent formulated with an ether electrolyte improved high voltage cyclability up to 4.4 V and a good ionic conductivity 11.52 mS cm^−1^. The modified electrolyte employed graphite ǀǀ LiNi_0.33_Mn_0.33_Co_0.33_O_2_ cells showed a capacity retention of 62% and 63% at voltage limit 4.4 V under operation at 25 °C and low temperature –20 °C, respectively. The synergistic effect of LiNO_3_ and VC and their ability to penetrate inner solvation shell of ether electrolyte involved in the film forming process. The restructured organic–inorganic layer acted as a protective layer suppressing the side reaction which is due to the electrolyte decomposition at a high cut‐off voltage.^[^ ^209^ ^]^ Dual‐salt electrolyte additive was reported by Wen et al. to effectively improve high‐voltage operation of Li‐metal batteries. Lithium salts, namely lithium trifluoroacetate and lithium nitrate (LiTFA–LiNO_3_) blended with carbonate electrolyte imparted multiple roles to stabilize high‐voltage operation of Li‐metal batteries; in situ generation of inorganic‐rich interphase, mitigating the moisture in electrolyte via an interaction between TFA^−^ and PF_6_^−^ anions, and strengthened interphase through NO_3_^−^ anions. The LiTFA–LiNO_3_ blended carbonate electrolyte offered high‐voltage operation of NMC532 and NMC622 cathodes up to 4.3 V and 4.4 V, respectively with a high reversibility, rate capability, and extended cycle‐life.^[^ ^210^ ^]^ Some of the chemical structures of functional additives reported for capacity retention and high‐voltage operation are portrayed in **Figure** **17**.

The redox limit of cathode material and the stability of an electrolyte dictates the high‐voltage operation. A co‐operation among the electrode–electrolyte component in conjunction with additive is necessary to improve the voltage limit. Layered cathodes LiCoO_2_, LiNi_x_Mn_y_Co_1‐x‐y_O_2_, LiNi_0.80_Co_0.15_Al_0.05_O_2_ and Li‐rich materials demonstrated an extended voltage stability in the presence of additive electrolyte mixtures. Functional additives, thiophene, diphenyl diselenide, and disiloxane even incorporated in traces, about 0.1–0.2 wt.% raised the voltage stability of LiCoO_2_ and Ni‐rich NMC cathodes. Combination of electrolyte additives SA+PS and LiNO_3_+LiTFA offered dual beneficial role as both SEI and voltage regulator, while LiNO_3_+VC additive mixture restructured the interphase via modulating the solvation shell. Boron‐based additives LiBFMB and LiBOB leveraged the structural changes and reformed Li‐rich cathode's CEI film. Functional additive incorporated electrolytes certainly stabilized the phase transformation, altered solvation shell, and robust interface leading to dominated surface chemistry of cathodes. As a result, stable cycling of Li‐ion/Li–metal batteries at high cut‐off voltage limit were achieved. However, limiting the concentration of additives below 5% and novel electrolyte formulation possessing high solvent stability, solute, and additive that are compatible with the high‐voltage cathodes would be ideal to achieve a higher cut‐off voltage.

### 4.3 Overcharge Protection Additives

**Figure 18:**
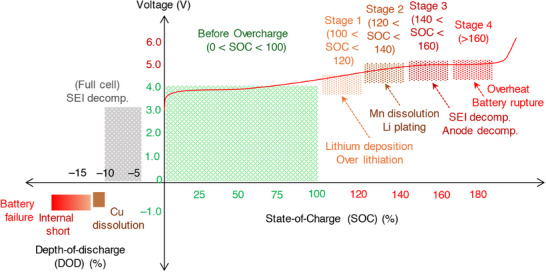
Summary of overcharge/over‐discharge conditions promoted challenges in a Li‐ion battery containing Ni─Mn cathode *vs*. graphite anode. The serious implication like physical‐chemical‐structural‐mechanical changes in a Li‐ion battery as a function of state‐of‐charge (SOC) and depth‐of‐discharge (DOD), respectively during overcharge/over‐discharge cycling conditions are summarized.

Overcharging a Li‐ion battery certainly damages the internal structure of a cell like severe electrolyte decomposition, abnormal side reactions, electrode deterioration, Li plating, etc. An overcharge condition in a Li‐ion battery measured in terms of state‐of‐charge (SOC) exceeding over 100% triggers a serious of concerns as illustrated in **Figure** **18**. During an overcharge, the generation of heat due to an excess energy induces a chain of safety breaching processes which may be an initial factor to cause “thermal runaway” in Li‐ion batteries. The collective risk factors triggered at an excessive SOC pose a severe safety issues experienced as overheat, explosion, and fire. On the other side, a minimal over‐discharge measured as depth‐of‐discharge (DOD) approaching about –10% collapse the internal contact in a Li‐ion battery.^[^ ^211^, ^212^ ^]^ Although, the over‐discharge does not impose safety issues, however, the cells suffer from SEI decomposition, dissolution of copper current collector and internal short‐circuit.^[^ ^213^, ^214^, ^215^ ^]^ From the perspective of a battery performance, even a minimal overcharge beyond the acceptable voltage limits (vary with battery's cell chemistry) could reduce the discharge capacity and an over‐discharge might end up in an increased cell impedance.^[^ ^216^, ^217^ ^]^ To overcome the overcharging issue, strategies such as its restriction, elimination, and early detection via smart electronics/chips are necessary to improve the Li‐ion battery safety.

Overcharge protectors or redox‐shuttle additives added to a liquid electrolyte protects the battery under overcharge conditions. In general, a compound that must meet the criteria as an overcharging protection additive ought to possess an intrinsic oxidation reaction that is above the charging voltage of a Li‐ion battery's cut‐off voltage limit.^[^ ^218^ ^]^ Apart, they are expected to be electrochemically stable within the operational voltage and readily soluble in an electrolyte solution retained for long term cycling. The function of a redox additive as an overcharge protector is such that they remain inactive or does not participate in any reaction under normal charging. Upon exceeding the nominal cut‐off voltage of a Li‐ion battery, the redox additive oxidizes at the positive electrode leaving the oxidized product to diffuse to the negative electrode to undergo reduction reaction. After the battery is charged, the redox additive shuttles between the positive and negative electrode absorbing excess charge. Thus, an establishment of an internal anti‐overcharge mechanism through the redox additives significantly improves the safety of Li‐ion batteries.

Additives for overcharging are mainly based on redox‐active inorganic compounds such as I^−^/I_2_ and Br^−^/Br_2_,^[^ ^219^, ^220^ ^]^ organic compounds containing N, O, and S, and coordination compounds like ferrocene. The inherent reduction–oxidation potential of overcharge protection additives determines their application in a suitable Li‐ion battery chemistry. The charge and discharge reactions of lithium iodide (LiI) in controlling the overcharge was studied in an electrolyte containing LiAsF_6_ in tetrahydrofuran (THF). During the overcharge process, the additive LiI undergoes oxidation to form I_2_ which was highly favourable for initiating the ring‐opening polymerization of THF molecule. To avoid such polymerization reaction, a high amount of LiI dissolved in a liquid electrolyte is preferred, such that stable species like LiI_3_ and I_2_ are produced. However, the LiI formed through the reaction between Li‐ions and I_2_ reduces the stability of SEI film formed on graphite anode, which is in contrary to the Li anode. Since, the redox potential of I_2_ falls close to 3.1 V versus Li/Li^+^, such additives are effective for 3 V–class Li‐ion batteries.^[^ ^192^ ^]^

Sulfur‐based compounds are also effective overcharge protectors which proceeds as follows, sulfur (S) experiences an oxidation reaction forming sufur radical (S**^·^**), which subsequently dissolves in the electrolyte and gets reduced to pristine sulfur on the anode side. A series of sulfur based thianthrene‐type compounds which include 2,7‐diacetylthianthrene (DATT), 2,7‐dibromothianthrene (DBTT), 2,7‐diisobutanoylthianthrene (DIBTT), and 2‐acetylthianthrene (ATT) were reported as overcharging protection additives. The nature of substituents attached to the thianthrene played a dominant role in dictating the redox potentials that ranged between 4.0–4.4 V versus Li/Li^+^. For instance, the presence of electron withdrawing bromine in 2,7‐dibromothianthrene is active at a redox potential of 4.4 V, while an unsubstituted thianthrene presented redox activity at 4.1 V.^[^ ^221^ ^]^ Ferrocene and its derivatives are other group of redox active overcharge protection additives. The redox potential of ferrocene could be tuned through the substituents and typically ranges between 3.0–3.5 V versus Li/Li^+^.^[^ ^222^ ^]^ The effect of dihydrophenanzine derivatives were studied as overcharging protection additive in liquid‐ or polymer‐based electrolyte Li‐ion batteries. Derivatives possessing 2‐hydroxypropyle and ethyl substituents on both nitrogen atoms are electrochemically tested with 1 M LiClO_4_ in DME: PC electrolyte. The hydroxy‐based dihydrophenanzine exposed a redox shuttle and induced potential limitations, while such behavior was not observed with N‐based dihydrophenanzine due to limited mobility and pronounced equilibrium constant for the complex formation.^[^ ^223^ ^]^

**Figure 19:**
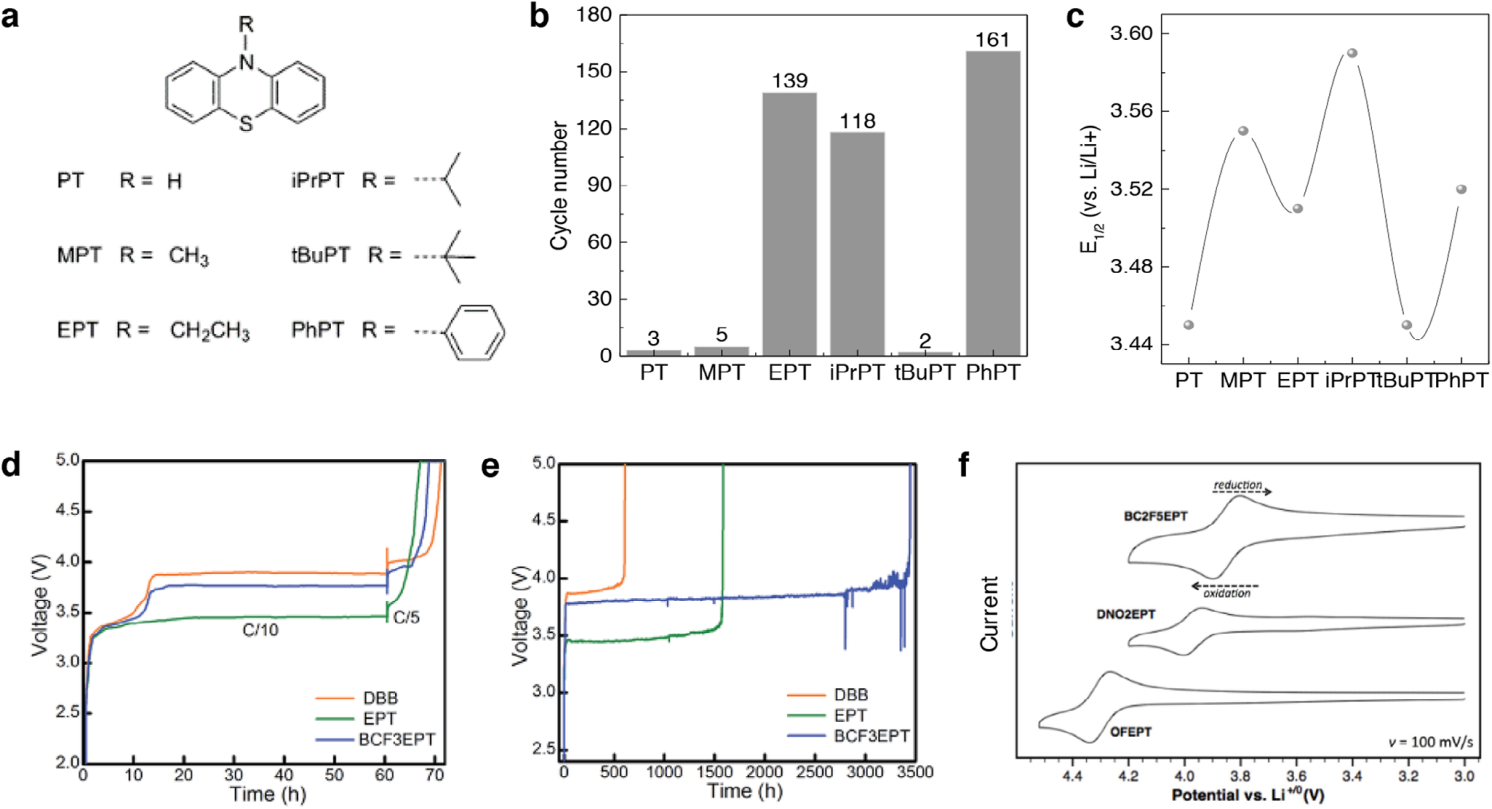
a) Structure of phenothiazine and its *N*‐substituted derivatives. b) Cycling stability of graphite ǀǀ LiFePO_4_ batteries using various phenothiazine, numbers represent the maximum number of cycles a battery survived. c) Oxidation potentials of phenothiazine *vs*. Li/Li^+^. d) Charge tests at 0.1 C rate for 60 h and 0.2 C for 25 h until 5.0 V. Reproduced with permission.^[^ ^227^ ^]^ Copyright 2015, Wiley‐VCH. d,e) Stability of BCF3EPT additive compared with DBB an EPT through charge tests d) at C/10 and C/5 and e) until the potential 5.0 V. Reproduced with permission.^[^ ^229^ ^]^ Copyright 2014, Royal Society of Chemistry. f) Cyclic voltammogram curves (Carbon as working, Pt as Counter, and Li foil as reference electrode) for various additives BC2F5EPT, DNO2EPT, and PFEPT dissolved in LiPF_6_‐EC:EMC electrolyte at a scan rate of 100 mV s^−1^. Reproduced with permission.^[^ ^230^ ^]^ Copyright 2016, Royal Society of Chemistry.

A series of alkoxybenzene compounds were screened as probable redox active overcharge protectors by Dahn et al. Compounds with redox couples that included 2,2,6,6‐tetramethylpiperidiniyl‐oxide (TEMPO), di‐*tert*‐butyl‐2,5‐dimethoxybenzene (DDB), and methylphenothiazine (MPT) were successful in overcharge protection that lasted for >100 cycles in a Li_4_Ti_5_O_12_ ǀǀ LiFePO_4_ Li‐ion battery. The DDB additive‐based electrolyte tested in graphite ǀǀ LiFePO_4_ configuration showed higher cycling stability for over 200 cycles.^[^ ^224^ ^]^ Several derivatives of TEMPO, 4‐methoxy‐TEMPO and 4‐cyano‐TEMPO were developed and were effective in preventing the overcharge. However, the lower redox potential of TEMPO within the charge potential of LiFePO_4_ cathode required replacing an electron withdrawing fluorine with hydrogen atom in the methyl groups. Some of the theoretically predicted TEMPO derivatives containing halogen substitution are 4,4‐difluro‐TEMPO, 3,3,5,5‐tetrafluro‐TEMPO, and 3,3,4,4,5,5‐hexafluro‐TEMPO.^[^ ^225^ ^]^ Phenothiazine based redox shuttle compounds with substituents R = CH_3_, OCH_3_, Cl, Br, and CF_3_ (**Figure** **19**a) were studied for their effect on overcharge protection. Among the substituted phenothiazine, methyl‐substituted *N*‐ethylphenothiazine possessed lowest oxidation potential than the cathode and thus could not meet the criteria for overcharge protection. Derivatives of phenothiazine containing chlorine and bromine formulated LiPF_6_ in EC:EMC electrolyte could limit the overcharge to certain extent.^[^ ^226^ ^]^ Further, phenothiazine substituted with alkyl chains and phenyl group were developed, where *N*‐ethylphenothiazine (EPT), isopropyl phenothiazine (*i*PrPT), and *N*‐phenyl phenothiazine (PhPT) exhibited overcharge cycling for >100 cycles in a Li_4_Ti_5_O_12_ ǀǀ LiFePO_4_ (Figure 19b) battery. In contrary, the derivatives methyl phenothiazine (MPT) and *N*‐tertbutyl phenothiazine (*t*BuPT) with smallest and largest steric substitution adjacent to *N*‐substituent could withstand only for fewer overcharge cycles. Among the above substituents, except PT and *t*BuPT, the oxidation potential in 1.2 M LiPF_6_ in EC: DMC (3:7) electrolyte for all the phenothiazine additives were above 3.5 V versus Li/Li^+^ (Figure 19c). The lower stability of *t*BuPT was found to be due to the decomposition forming PT, when the compound is oxidized.^[^ ^227^ ^]^

The redox potential of phenothiazine was increased through the 3,7‐disubstituted derivatives of ethylphenothiazine. Modifying the electron‐donating and electron‐withdrawing substituents enabled to tune the redox shuttle of desired 3,7‐disubstituted *N*‐ethylphenothiazine. Through 3,7‐disubstitution, several additives were developed which included 3,7‐dichloro‐*N*‐ethylphenothiazine (DCIEPT), 3,7‐dibromo‐*N*‐ethylphenothiazine (DBrEPT), 3,7‐dimethyl‐*N*‐ethylphenothiazine (DMeEPT), 3,7‐bis(trifluromethyl)‐*N*‐ethylphenothiazine (BCF3EPT), and 3,7‐dicyano‐*N*‐ethylphenothiazine (DCNEPT). Among the above derivatives, BCF3EPT additive about 0.08 M formulated 1.2 M LiPF_6_ in EC: EMC electrolyte demonstrated improved performance for the graphite ǀǀ LiFePO_4_ batteries with 100% overcharge protection. Despite the halogenated derivatives of ethylphenothiazine displaying higher oxidation potential, the polarizability played a dominant role in achieving improved cycling stability.^[^ ^228^ ^]^ The BCF3EPT redox compound along with EPT and DBB survived C/10 charging (Figure 19d) and cycled at 0.1 C until 5.0 V (Figure 19e), additive BCF3EPT showed better stability of 3448 h against 1580 h and 606 h for EPT and DBB, respectively in LiPF_6_ in EC: EMC electrolyte. The diffusion co‐efficient for both the neutral and oxidized species of BCF3EPT were comparable with that of the DBB and EPT redox shuttles and relatively exposed higher cycle life at high voltage.^[^ ^229^ ^]^ Further, the role of electron withdrawing groups such cyano‐, fluoro‐, and nitrate‐ attached on to periphery of phenothiazine were found to increase the redox potential. Among the redox shuttle, *N*‐ethyl‐3,7‐bis(pentafluroethyl)phenothiazine (BC2F5EPT), *N*‐ethyl‐3,7‐dinitophenothiazine (DNO2EPT), and *N*‐ethyl‐1,2,3,4,6,7,8,9‐octaflurophenothiazine (OFEPT), the oxidation peak (Figure 19f) for OFEPT occur at 4.30 V (versus Li/Li^+^), while BC2F5EPT and DNO2EPT compounds showed about 3.86 V and 3.97 V, respectively. The modified OFEPT derivative‐based carbonate electrolyte enabled the high voltage LiNi_0.8_Co_0.15_Al_0.05_O_2_ (NCA) and graphite anode to successfully cycle for over 450 h under an overcharging condition.^[^ ^230^ ^]^

Silane‐based dimethoxydiphenylsilane (DDS) showed overcharge protection through the formation of polymerized layer. Typically, the DDS additive exposed to overcharging at 4.9 V polymerized and built a safe layer of polymer on the surface of the electrode and separator. The electrolyte containing DDS does not significantly alter the ionic conductivity and the electrochemical performances of LiCoO_2_ cathode, however a stable overcharge protection was under action.^[^ ^231^ ^]^ Feng et al. designed and developed a resorcinol bis(diphenyl phosphate) (RDP) by single‐step phosphorylation reaction. The RDP additive presented a good compatibility with LiPF_6_ in EC:EMC electrolyte, where an increase in the content of RDP from 0 to 72% suppressed the flammability of electrolyte down to 0 s g^−1^ from 49 s g^−1^. The graphite ǀǀ LiMn_2_O_4_ cells containing RDP‐based electrolyte stabilized at 4.4 V even at 100% overcharge. It was concluded that the overcharge protection of RDP arises due to the electrochemically triggered polymerization at 4.4 V which consume the overcharge current.^[^ ^232^ ^]^ Additive that are effective SEI film‐forming agents too showed better overcharge protection capability. Vogl et al. studied N‐ethyl‐2‐pyrrolidone (NEP) as an overcharge protection additive in LiFePO_4_ and LiMn_2_O_4_ cathodes. The oxidation products observed on the surface of the overcharged cathode were studied through XPS. In the presence of NEP, the LiFePO_4_ composed of thick layer of decomposition products containing carbon and oxygen prevented overcharging, while the dissolution of Mn and MnO formation is lowered in the LiMn_2_O_4_ cathodes.^[^ ^233^ ^]^

Overcharge/discharge protectors are redox‐active compounds, they oxidize at the positive electrode developing an oxidized product which diffuses to the negative electrode and experiences a reduction reaction. Under overcharge conditions, the redox additive shuttles between the positive and negative electrode absorbing excess charge. Especially, elements Br and I based compounds were active overcharge protectors. Sulfur‐based thianthrene compounds DATT, DBTT, DIBTT, and ATT upon ooxidation released soluble sulfur radical (S**^·^**), which further reduced to pristine sulfur acting as a barrier on the anode electrode. Phenothiazine derivatives are effective redox shuttle compounds, modulating the electron withdrawing and electron‐donating substituents R = CH_3_, OCH_3_, Cl, Br, and CF_3_ influenced the oxidation potential and cycling stability. Such overcharge protecting‐additives could be relied upon to certain cycles of over‐charge/discharge. However, prolonged prevention needs a high amount of additives, which may likely hamper the electrochemical performance‐based properties. Alongside, overcharge protecting‐additives, a smart sensing device could provide robust protection to the Li‐ion/Li–metal batteries over an extended lifetime.

### 4.4 Wide‐Temperature Operation Additives

**Figure 20:**
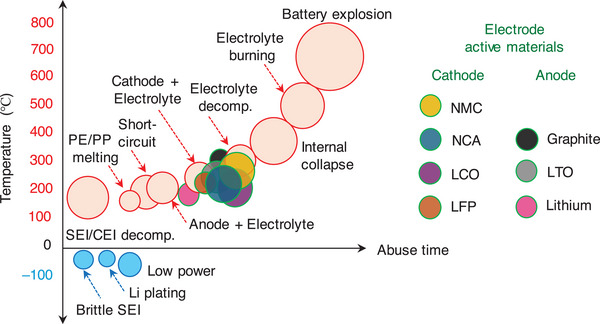
Categorical operational challenges of Li‐ion/Li–metal batteries as a function of temperature and abuse time. The battery components considered are anodes including graphite, Li_4_Ti_5_O_12_, and Li metal, polypropylene/polyethylene separator, carbonate electrolyte, and commercial oxide and phosphate based lithiated cathode materials.

In general, the operational temperature limits of commercial carbonate electrolyte‐based Li‐ion batteries fits within a narrow range of –20 °C to 55 °C for discharge and 0 °C to 45 °C for charge.^[^ ^234^ ^]^ The temperature surrounding the battery such as low and high temperature significantly influence the electrochemical performance of a Li‐ion battery. The stability of an electrolyte solution under extreme temperature is governed by the parameters *T*_b_ and *T*_m_ of the solvent. Typically, the Li‐ion battery being exposed to wide operating temperature conditions are likely to suffer from altered electrolyte properties, changes in the physicochemical properties of electrolyte and electrode and their interfacial components. From an electrochemical viewpoint, an altered ion migration and its diffusion, ionic conductivity, and resistances across the electrode and electrolyte interface is inevitable. The changes observed in a Li‐ion battery associated with an increase in the temperature as a function of time are schematically illustrated in **Figure** **20**. The respective variations observed as high–low temperature versus abuse time are plotted considering various battery chemistries consisting of LFP, LCO, NMC, and NCA as cathodes and Li metal, LTO, and graphite as anodes in carbonate electrolytes. This section focuses on improving the interfacial properties, ionic conduction, and resistances of electrode‐electrolyte at wide‐temperature operating zones.

#### 4.4.1 Low–Temperature Cycling

The basic criteria for designing a low‐temperature electrolyte are, low melting point, limited Li‐ion affinity, and stable interface. Alongside, the viscosity of a liquid electrolyte must be retained to realize a good ionic mobility and wettability of an electrode. In general, the change in the viscosity of a liquid electrolyte in Li‐based batteries is possible under two conditions, typically during the charge‐discharge process, which is minimal and any temperature variations during the operation. Electrochemically, the change in the viscosity of electrolytes are common for the Li‐S batteries. The sulfur electrokinetics during the discharge process proceed through a sulfur to polysulfides transition involving subsequent solid‐to‐liquid (S_8_→Li_2_S_4_) and liquid‐to‐solid (Li_2_S_4_→Li_2_S) phases. During the transition S_8_→Li_2_S_4,_ the solid S_8_ converts to Li_2_S_4_ and dissolves in a liquid electrolyte and further transitions to solid Li_2_S state.^[^ ^235^ ^]^ Considering the influence of temperature; at a low temperature, the conventional electrolytes are prone to an increase in the viscosity and solvent dependent crystallization together leads to failure of batteries. The temperature‐related parameters *T*_b_ and *T*_m_ of electrolyte solvents critically influences the viscosity of a liquid electrolyte at an elevated and low temperature. A summary of the physical properties of ester and ether solvent for Li‐metal and Li‐ion batteries are provided in **Table** **2**. The dependence of an electrolyte viscosity on the temperature being observed from the electrochemical perspective are as follows; an elevated charge transfer resistance, slow Li^+^ transport kinetics, increased interface resistance, suppressed mobility of Li^+^ at electrode–electrolyte interphase, and reduced ionic conductivity. Apart, the properties of Li kinetics differ largely at the electrodes, where a slow Li‐ion diffusion promotes Li plating on the graphite surface under low temperature cycling.^[^ ^236^ ^]^ Since, the content of EC is higher in the commercial electrolytes, an attempt to reduce the EC amount likely impacts the interfacial properties.^[^ ^237^ ^]^ Formulating electrolytes with a low melting point solvent or a co‐solvent mixture could control the solidification of liquid electrolyte at low temperature. A more comprehensive understanding and the principles governing the design of low temperature electrolyte for Li‐ion batteries are reviewed elsewhere.^[^ ^238^, ^239^ ^]^

**Table 2:** Summary of physical properties of ester and ether solvent for Li‐metal and Li‐ion batteries.

Solvent	*T*_m_ [°C]	*T*_b_ [°C]	*T*_f_ [°C]	Dielectric constant [*ε*]	Density *ρ* [g cm^−3^]	Viscosity *η* [cP]
Ethylene carbonate	36.4	248	160	89.8	1.32	1.9
Propylene carbonate	−49	242	132	65	1.20	2.53
Dimethyl carbonate	4	90	18	3.1	1.06	0.63
Diethyl carbonate	−74	126	31	2.8	0.97	0.75
Ethyl methyl carbonate	−53	110	−	2.96	1.01	0.65
Ethyl acetate	−84	77	−3	6.0	0.90	0.45
Methyl acetate	−98	57	−13	6.7	0.93	0.40
Methyl butyrate	−85.8	102.8	11	5.48	0.90	0.54
Ethyl butanoate	−93	120	19	−	0.88	0.71
Methyl propionate	−87.5	79.8	−2	6.20	0.92	0.43
1,3‐dioxolane	−95	78	2	−	1.07	–
Dimethoxyethane	−58	83	−9	–	0.87	0.45
Dimethyl sulfoxide	18.5	189	88.9	46.7	1.1	2.0
Tetrahydrofuran	−136	75	−14.5	–	0.86	0.53
Sulfolane	27.5	285	165	44	1.26	0.01
Dimethyl formamide	−60.4	153	58	37	0.94	0.8
Diglyme	−64	162	57	7.23	0.94	1.88
Tetraglyme	−45	216	106	7.9	0.99	2.73

**Figure 21:**
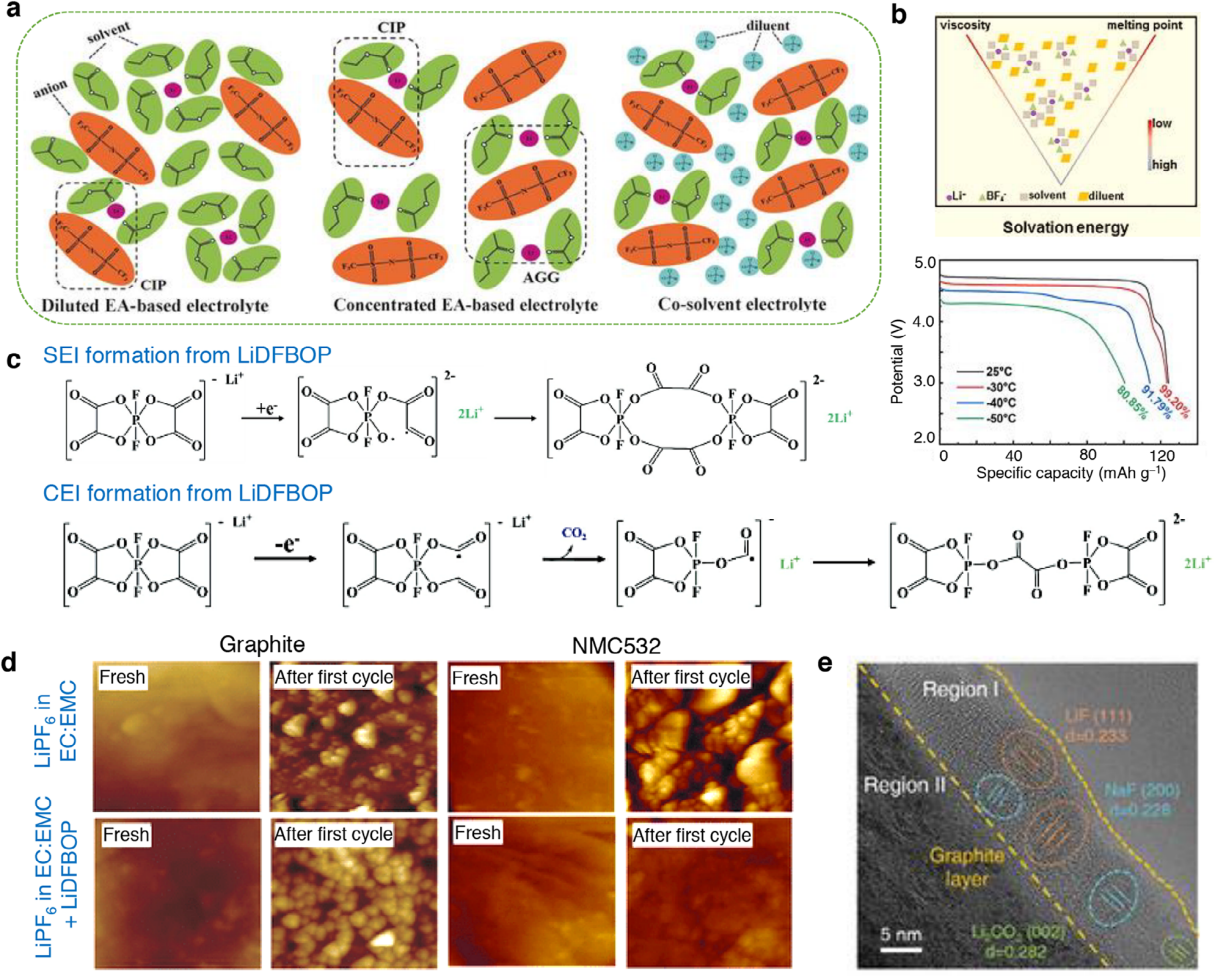
a) Schematics illustrating the solvation structure of electrolytes; diluted, concentrated, and co‐solvent electrolytes. Reproduced with permission.^[^ ^241^ ^]^ Copyright 2019, Wiley‐VCH. b) Localized high‐concentration carbonate electrolyte based on methyl acetate and charge profile of LiNi_0.5_Mn_1.5_O_4_ cathode showing good capacity at low operation temperature. Reproduced with permission.^[^ ^244^ ^]^ Copyright 2022, Elsevier. c) Mechanism of SEI and CEI formation in the presence of lithium difluorobis(oxalate) borate (LiDFBOP) additive. d) AFM images of fresh graphite and NMC532 electrodes and cycled ones tested with LiPF_6_ in EC:EMC and LiDFBOP containing electrolytes. Reproduced with permission.^[^ ^250^ ^]^ Copyright 2018, Wiley‐VCH. e) Cryo‐TEM analysis of SEI on graphite anode using FWSE+Na electrolyte. Reproduced with permission.^[^ ^253^ ^]^ Copyright 2023, Wiley‐VCH.

A mixture of carbonate solvent EC/EMC with 30 mol.% of EC possess liquidus point (point at which first solid particles develop) above 0 °C, which is still fractional for the operation at low temperature. Significant research efforts have been undertaken to optimize the electrolyte formulations involving Li salts, solvents, co–solvents, and additives to realize a stable and smooth operation of Li‐based batteries at low/high temperature. The consensus of using HCEs was reported to certainly improve the electrochemical stability due to an increased concentration of Li salts. For instance, Ming et al. developed a moderately high concentration 1.2 M LiPF_6_ electrolyte in EMC and methyl acetate (MA) solvents. The unique solvated ions endowed through the EMC+MA electrolyte composition developed a stable interface and also restricted the undesirable decomposition.^[^ ^240^ ^]^ Co‐solvents or diluents formulated with concentrated electrolytes is an effective strategy to improve the localized concentration with low viscosity and strong oxidation resistance resulting in stable operation of cell at a low temperature. The addition of cyclic carbonate PC as a co‐solvent with EC showed effective in suppressing the crystallization, which is why PC is the most regarded solvent choice for low temperature Li‐ion batteries. Dong et al. reported dichloromethane (DCM) as an inert co‐solvent to a concentrated ethyl acetate (EA)–based electrolyte. Taking advantage of limited solubility, low freezing point (–95 °C) and low viscosity (0.44 MPa s) of DCM solvent, the developed electrolyte formulation enabled the concentrated electrolyte to retain the original solvation structure as shown in **Figure** **21**a. Furthermore, several properties of the DCM‐based electrolytes such as viscosity at 0.35 Pa s, enhanced voltage window up to 4.85 V, and ionic conductivity of 0.6 mS cm^−1^ at a low operating temperature of –70 °C were realized. As a result, the Li ǀǀ polyimide ǀǀ Li battery that employed a DCM based concentration gradient electrolyte exhibited specific energy of 178 Wh kg^−1^ at 64 W kg^−1^ of specific power with a power density of 17 Wh kg^−1^ at a specific power of 2877 W kg^−1^.^[^ ^241^ ^]^ Chen et al. demonstrated the influence of local solvation structure on the charge transfer behaviors of Li metal at a critically low temperature. Diethyl ether possessing a low melting point of –116 °C could remain stable and the respective electrolyte formulation's solvation sheath evolves into contact‐ion pair structures, which deviated from the common solvent‐separated ion pair structures that prevail in an ether electrolyte.^[^ ^242^ ^]^ Systematic understandings reveal that the de‐solvation process is the limiting step for the low‐temperature operation of Li‐based batteries. A balanced solvation/de‐solvation of a weakly‐solvating electrolyte formulated as LiFSI in acetonitrile and fluorobenzene electrolyte enhanced the Li‐ion diffusion in the bulk medium and improved Li‐ion de‐solvation kinetics. The graphite half cells infiltrated with a weakly‐solvating electrolyte demonstrated a capacity of 302.7 mAh g^−1^ at a high current rate of 8 C and good cyclability at low temperature.^[^ ^243^ ^]^

The film forming ability of an electrolyte is crucial, does apply for batteries exposed or operated at a low temperature. Methyl acetate‐based concentration electrolyte was developed with a typical formulation of 4 M LiBF_4_ in FEC/MA/1,1,2,2‐tetrafluroethyl methyl ether (TFME) which showed an optimal performance at a low temperature operation (Figure 21b) due to a balanced viscosity and melting point. The highly concentrated electrolyte containing TFME as a co‐solvent maintained a stable SEI film at the graphite anode eventually due to the existence of LiF richness on the surfaces. The synergy between LiF and the solvent FEC certainly decreased the interfacial resistance of CEI film. The Li‐ion battery configured as Li ǀǀ LiNi_0.5_Mn_1.5_O_4_ employing the MA‐based electrolyte exhibited a discharge capacity of 80.85% at a current rate of 0.2 C under –50 °C (Figure 21b) when compared to a room temperature cycling. Apart, the electrochemical window broadened to about 5.4 V enable to achieve a stable SEI film possessing abundance of LiF at the anode.^[^ ^244^ ^]^ Liao et al. reported that the capacity of graphite‐based anode at a low temperature of –40 to 20 °C could be improved by the addition of FEC, about 2 wt.%. The formation of a smooth and conductive interphase due to the FEC component certainly reduced the charge transfer interphase at a low temperature contributing to an improved electrochemical performance.^[^ ^245^ ^]^ Further, adding a small fraction of FEC about 0.5 wt.% improved the cyclability of graphite ǀǀ LiNi_0.33_Mn_0.33_Co_0.33_O_2_ cells at –40 °C.^[^ ^246^ ^]^ Lv et al. developed a dual‐salt LiPF_6_ electrolyte containing a mixture of additives butyl acrylate (BA), EC, and LiBF_4_. A combination of properties from the additive and solvent such as low melting of BA and the film forming ability of EC, respectively along with high dielectric constant benefited cyclability at –40 °C. Mixed additives LiBF_4_+BA+EC containing LiPF_6_ in DMC: EMC (3:5, w/w) improved the solubility, melting point, and conductivity of the electrolyte. At a low cycling temperature –40 °C, the specific capacity using NMC811 cathode was about 119.3 mAh g^−1^ against 186.5 mAh g^−1^ at 25 °C.^[^ ^247^ ^]^ Fluoroethylene carbonate, a known film forming additives for the graphite anode was extended as an additive for low temperature operation of graphite ǀǀ LiFePO_4_ based Li‐ion batteries. At a concentration of 2 vol.% FEC, the cyclability and rate performance were improved at low temperature, where the specific capacity of FEC‐based electrolyte showed 22% increment in the capacity than the pristine electrolyte at –20 °C. It was noted that an improved ionic conductivity of SEI film developed through the FEC additive enabled faster migration of ions at the electrode/electrolyte interphase.^[^ ^248^ ^]^ Sultone as an additive demonstrated a good film forming ability with graphite anode, while also supported low temperature operation. The introduction of small amount of PS < 0.5 wt.% in 1 M LiPF_6_ in PC/DEC (1:1 vol.) and EC: DEC (7:3 vol.) electrolytes facilitated the intercalation of Li‐ions, while remained effective even upon cycling at –5 °C.^[^ ^249^ ^]^

The interface developed on both the anode and cathode are due to the decomposition of electrolyte solvents. In particular, the interface developed via salt‐type additives are more ionically conductive than the one developed through the decomposition of electrolyte and organic additives. Liao et al. reported a new strategy where the decomposition of lithium difluorobis(oxalate) phosphate (LiDFBOP) enabled simultaneous construction of low impedance interface on both the anode (SEI) and cathode (CEI) as illustrated in Figure 21c. The LiDFBOP additive in combination with 1 M LiPF_6_ in EC/EMC (1/2 wt.%) electrolyte tested in a graphite ǀǀ LiNi_0.5_Co_0.2_Mn_0.3_O_2_ cells showed a capacity retention of 49% at –30 °C, while the base electrolyte cells displayed only 14% retention. The preferential decomposition of LiDFBOP than the bulk electrolyte components developed interface that is quite ionically conductive at low temperature while also remain protective. The surface of the cycled graphite anode and NMC532 cathode were relatively smooth (Figure 21d) in the presence of LiDFBOP than the LiPF_6_ based electrolytes.^[^ ^250^ ^]^ Phosphor based organo‐compounds as additives are also effective to improve low temperature operation of Li‐ion batteries. Tris(trimethylsilyl)phosphite (TMSP or TMSPi) additive was found to limit the overpotential of graphite ǀǀ LiNi_0.8_Co_0.15_Al_0.15_O_2_ cells at –40 °C. A low dosage of about 0.5 wt.% TMSP formulated with a carbonate electrolyte showed an improved capacity. An electrolyte solvation strategy was adopted to improve the low temperature operation and fast charging aspects of NMC cathode and Li metal anode. Modified electrolyte developed by substituting EC with FEC and 1,2‐difluorobenzene as a diluent facilitated the Li‐ion de‐solvation kinetics through the intermolecular interaction between the fluorobenzene and solvent molecules.^[^ ^251^ ^]^ Fluorinated compounds as a part of electrolyte solvent offered good ionic conductivity, wide electrochemical window, and flame retardant property. On the other side, the lower temperature operation of non‐aqueous fluorinated electrolytes is not regarded as a good choice due strong affinity between Li‐ion and fluorinated solvents. Fan et al. employed FEC into highly fluorinated non‐polar solvents to improve the operation temperature limits of NCA cathode. Electrolyte formulation based on FEC and salts LiFSI or lithium bis(pentafluoroethanesulfonyl)imide (LiBETI) with diethyl carbonate (DEC), fluoroethylene carbonate, methyl(2,2,2‐trifluoroethyl) carbonate in either tetrafluoro1‐(2,2,2‐trifluoroethoxy)ethane or methoxyperfluorobutane were designed. Modified electrolyte not only showed a wide temperature operation range from –125 to 70 °C but also wide voltage window 0.0 to 5.6 V. The specific capacity and cycling stability of Li ǀǀ NCA cells in the presence of 1.28 M LiFSI‐FEC/FEMC were found to be better than the 1 M LiPF_6_ in EC:DMC electrolyte at both low and high temperature operation.^[^ ^252^ ^]^ Heteroatom cation‐based additive was proposed by Zheng et al. to tune the solvation and interfacial structure of graphite and Ni‐rich cathodes. The preferential reduction of Na^+^–(solvent/anion) facilitated the migration of Li‐ions through the LiF/NaF boundaries. As a result, the graphite ǀǀ NMC532 cells configured with N/P ratio 1 demonstrated a discharge capacity of 109 mAh g^−1^ with an energy density of 270 Wh kg^−1^ at a low temperature of –40 °C. Ex situ cryo‐TEM analysis of cycled graphite anode revealed bi‐layered regions (Figure 21e), outer layer dominated with LiF and NaF species followed by carbonate salt based inner region which is in contact with graphite.^[^ ^253^ ^]^

Resistive and fragile interphase and poor ionic conductivity of Li‐based batteries at a low temperature collectively limit the electrochemical performance. Solvents of low freezing points introduced as co‐solvents or diluents and altering the solvation chemistry of electrolytes via additive chemistries certainly controlled the properties of electrolyte like viscosity and ionic mobility. Introducing co‐solvents PC, FEC, DCM, TFME, PS etc., about 0.2–5 wt.% suppressed the crystallization while favorable for improving the ion conductivity of electrolyte. In addition, the conductive and stable interface constructed in the presence of additives altered the kinetics at a low temperature. Self‐sacrificial activity of LiDFBOP and Li‐ion de‐solvation using EC+FEC with fluorobenzene, respectively afforded reasonable conductivity at low temperature. Since most of the electrolytes were specific to the graphitic anode, engineering electrolyte formulations for a variety of electrode chemistries are demanding. Furthermore, a comprehensive understanding of the properties of solvent, salt, and its compatibility at a low temperature are needed.

#### 4.4.2 High‐Temperature Cycling

The geologically diverse climatic conditions, like high altitude and desert environment, present formidable challenges for the normal operation of batteries, especially application like e‐mobility or utilities in aerospace or defense sectors. It is worth noting that the batteries stationed at temperature, either cold or hot zones could be accustomed to climatic changes. However, applications like electric vehicles or handheld devices need an electrochemically triggered protection like using additives where the Li‐based batteries could fit with the narrow spectrum of temperature −20–55 °C. Operation of batteries at an elevated temperature certainly accelerates the reactivity at the interface leading to an arbitrary growth, eventually a thicker SEI. Furthermore, the low flash point electrolyte solvents are susceptible to adverse safety issues at higher temperatures. Likely, the carbonate‐based Li‐ion battery electrolytes could withstand a temperature of 55 °C for discharge and 45 °C for charge.^[^ ^234^ ^]^ As the temperature exceeds 60 °C, the LiPF_6_ salt loses its stability and its thermal decomposition producing solid LiF and gaseous PF_5_ species. Furthermore, an aberrated condition arises in the presence of water contaminants, where the LiPF_6_ decomposition is accelerated, leading to corrosive hydrofluoric acid.^[^ ^254^ ^]^ Such a corrosive environment erodes the active materials and the current collector, leading to a failure of Li‐ion battery. For instance, the spinel LiMn_2_O_4_ cathodes succumb to HF attack at different state of charge leading to several detrimental conditions like irreversible and insulative side products like MnF_2_, increased surface resistance, continuous metal dissolution, exposed surface, and drastic capacity fade.^[^ ^255^ ^]^ Addition of VC as an additive to the EC‐based electrolyte showed an improved cycling performances and high temperature storage.^[^ ^84^ ^]^ The effect of 2,2‐dimethoxy‐propane (DMP) as an additive was studied in a carbonate based 1 mol dm^−3^ LiPF_6_ in EC: DEC (1:1 w/w) electrolyte for half and full cell Li‐ion batteries. Although, the scavenging property of DMP toward water is well known, the film forming ability with graphite anodes at elevated temperature could collectively improve the cyclability of Li‐ion batteries. Even at a low concentration of DMP about 0.005 wt.%, the modified electrolyte showed the lowest irreversible capacity of 6.32% against 7.49% for the control electrolyte. The Li‐ion batteries tested at a wide range of temperatures (23, 45, and 60 °C) showed similar electrochemical properties, where the of DMP revealed that the properties of SEI film modified with electrolyte. Electrochemical cycling of graphite ǀǀ LiCoO_2_ full cell employing DMP based electrolyte at 60 °C showed better discharge properties, which was eventually due to the development of a stable SEI film with DMP additive at a high temperature.^[^ ^256^ ^]^ Dimethyl acetamide (DMAc) was used as an additive to improve the thermal stability and SEI film in a LiPF_6_ EC:DMC:DEC (1:1:1 wt. ratio) electrolyte. The addition of about 1% DMAc enabled the liquid electrolyte to remain stable at a high temperature of 85 °C for about 6 months without any noticeable precipitation and colour change. Using DMAc additive‐based electrolyte, the discharge capacity of graphite ǀǀ LiFePO_4_ cells tested at 60 °C showed a higher retention of about 87.2% than the pristine electrolyte at about 55%. The improved thermal stability of LiPF_6_ brought by DMAc was due to the suppression of autocatalytic thermal decomposition of the electrolyte via the formation of a stable PF_5_–DMAc complex.^[^ ^257^ ^]^ A combination of additives 1,3‐propane sultone and vinylene carbonate in 1 M LiPF_6_ in EC: EMC (3:7, vol.) were found to improve the cycling performance of Li‐ion batteries at a high temperature of about 55 °C. Differential capacity plots revealed that the additives VC and PS are reduced prior to the reduction of EC forming an altered SEI structure. The formation of alkyl sulfonate and poly(VC) through the decomposition of PS and VC developed an SEI which possessed high ionic conductivity and thermal stability.^[^ ^258^ ^]^

**Figure 22:**
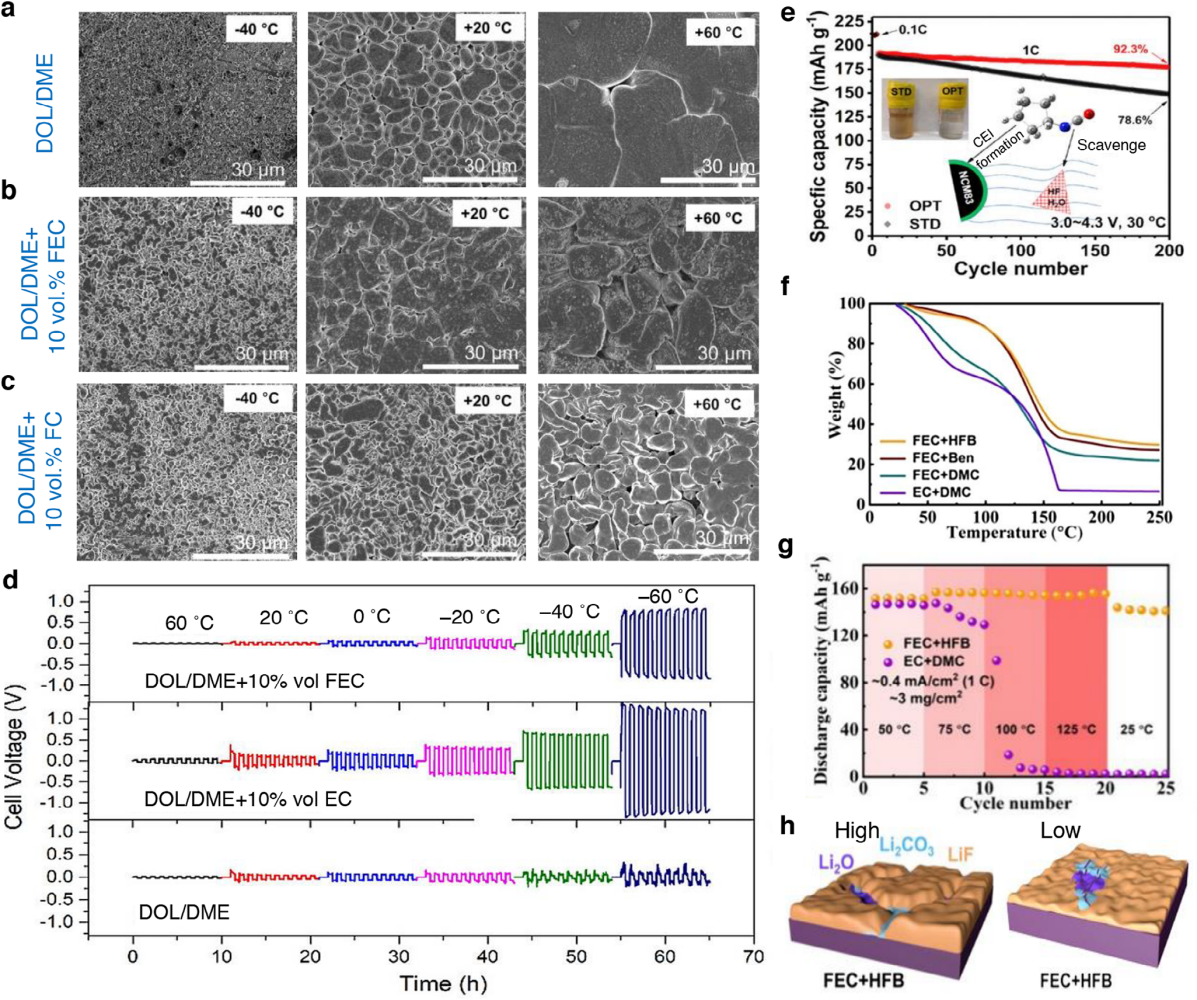
a–c) SEM micrographs depicting the morphological changes of Li deposition on a stainless steel at wide‐operating temperature –40, 20, and 60 °C using DOL:DME, DOL:DME+10% FEC, and DOL:DME+10% FC electrolytes. d) Lithium plating/stripping studies at a wide‐temperature limit using three different electrolyte formulation. Reproduced with permission.^[^ ^261^ ^]^ Copyright 2020, American Chemical Society. e) Cycling stability of NMC cathodes obtained using LiPF_6_ in EC:EMC blank and cyclopentyl isocyanate+LiPF_6_ in EC:EMC electrolyte. Reproduced with permission.^[^ ^270^ ^]^ Copyright 2021, American Chemical Society. f) Thermal stability of various electrolytes studied through thermogravimetric analysis. g) Cycling performance of Li metal anode and LiFePO_4_ cathode obtained using LiPF_6_ in EC:DMC and FEC+HFB electrolyte at wide‐temperature. h) Schematic illustration of SEI layer developed in the presence of FEC+HFB additive based electrolyte at high and low temperature. Reproduced with permission.^[^ ^271^ ^]^ Copyright 2024, American Chemical Society.

Phosphor based additives are effective to improve the cycling performance of Li‐ion batteries at room and high temperature. Xu et al. reported that the addition of binary additives containing TMSP and 1,3‐propanediolcyclic sulfate (PCS) to 1 M LiPF_6_ in EC/DEC/EMC carbonate electrolytes improved the cycling performance of graphite ǀǀ LiNi_0.5_Mn_0.5_O_4_ cells at room temperature and at 50 °C. The Li‐ion cell containing a binary additive (TMSP+PCS)‐based electrolyte tested at 50 °C exhibited a high‐capacity retention of 79.5% after 200 cycles at a current rate of 1 C. It was noted that the capability of TMSP toward scavenging the generated HF acid and stabilizing LiPF_6_ supported the cell to outperform at high temperature.^[^ ^194^, ^259^ ^]^ Further, aliphatic ester methyl acrylate in combination with (TMSP and PCS) was used as a co‐solvent with 1 M LiPF_6_ in EC/DEC/EMC electrolyte to improve the cycling performance of LiNi_0.5_Mn_1.5_O_4_ cathode. The quaternary electrolyte system containing graphite ǀǀ LiNi_0.5_Mn_1.5_O_4_ Li‐ion battery showed cycling capability at a wide temperature ranging –60 °C to 50 °C. A good capacity retention of 99.7% after 200 cycles was notable even at –5 °C, while the blank electrolyte‐imposed cell presented 74.3% retention. At a high temperature of 50 °C, the blank electrolyte exhibited a capacity of 62.8 mAh g^−1^, whereas a high capacity of 108 mAh g^−1^ was obtained for the modified electrolyte. It was noted that suppressing the reactivity of MA and MCMB carbon via binary functional (TMSP+PCS) additive was crucial to improve the cycling stability of high voltage cathode at low temperature cycling.^[^ ^260^ ^]^ A combination of electrolyte solvents about 10 vol.% of either EC or FEC in a base DOL:DME mixture improved the Li plating/stripping behavior at both low and high temperatures (**Figure** **22**a–d). The SEM micrographs portrayed in Figure 22a depicted the morphology of Li deposited on a stainless steel at 4 mAh cm^−2^ as a function of temperature with three different combinations of electrolyte, namely, DOL:DME, DOL:DME+FEC and DOL:DME+EC. Typically, at a high temperature of 60 °C, the particle size decreased following the order pure, FEC, and EC‐modified electrolyte. In contrary, at 20 °C, DOL:DME+FEC electrolyte showed high particle sizes.^[^ ^261^ ^]^

Lithium bis(oxalate)borate was used to protect the surface of Li‐rich Li_1.17_Ni_0.17_Mn_0.5_Co_0.17_O_2_ cathodes from the electrolyte decomposition. The carbonate electrolyte 1.3 M LiPF_6_ in EC:EMC:DMC containing LiBOB additive enabled the Li‐rich cathodes to be stable via the formation of a stable protective layer composed of semi carbonate‐like components. A high discharge capacity of 194 mAh g^−1^ and a capacity retention as high as 77.6% was attained at 60 °C. Notably, the cyclability of Li‐rich cathodes were enhanced both at room and high temperature through the LiBOB additive‐based electrolyte, as the decomposition of LiPF_6_ and HF formation are largely suppressed. The surface chemistry of CEI developed in the presence of LiBOB gave notable effects; it was likely that the superoxide (O_2_^−^) anion formed from the reduction of O_2_ gas evolved due to the activation of Li_2_MnO_3_ (discharge process) may get trapped by the electron deficient boron of LiBOB. The resultant CEI acted as a barrier to alleviate the undesired decomposition of electrolyte due to the O_2_ attack.^[^ ^262^ ^]^ Xu et al. reported a boron‐based additive, lithium tetramethyl borate (LTMB, LiB(OCH_3_)_4_ as a film forming additive for high voltage cathodes. Carbonate based electrolyte 1.2 M LiPF_6_ in EC:EMC (3:7 vol.) formulated with LTMB showed good cyclability of graphite ǀǀ LiNi_0.5_Mn_1.5_O_4_ cells even at 55 °C. The presence of borate based passivating layer was found to be beneficial for improving the electrochemical performance.^[^ ^263^ ^]^ Lithium bis(2‐methyl‐2‐fluromalonato)borate (LiBMFMB) as an additive showed improved cyclability of LiNi_0.5_Mn_1.5_O_4_ cells. It was reported that the concentration of LiBMFMB is essential, addition of 0.05 M LiBMFMB additive formulated with LiPF_6_ in EC:DEC:DMC electrolyte presented a C.E of about 96.9%, which increased further to 99.6% after 200 cycles, while 0.02 M displayed about 98.5%.^[^ ^264^ ^]^ Qiao et al. designed a bulky anion‐based Li salt lithium trifluro(perfluro‐tert‐butyloxyl) borate (LiTFPFB) as an additive, where the F^−^ in LiBF_4_ was substituted with a fluoroalkoxyl group. The developed LiTFPFB exhibited a better compatibility with Al current collector than the LiBF_4_ and LiTFSI based electrolytes. The Li Cu cells containing LiTFPFB/PC electrolytes demonstrated a C.E of 80.6% at the end of 50 cycles against 60.3% for LiBF_4_‐based electrolyte.^[^ ^265^ ^]^ Further, Han et al. designed lithium diflurobis(oxalate) phosphate (LiDFBP) additive, where two oxalate groups and two fluorine atoms are bonded to a central phosphorous core. Lithium‐rich electrodes cycled using LiDFBP‐based LiPF_6_ electrolyte underwent minimal phase transformation from layered to spinel‐like and rock‐salt structure, where the SEI thickness were about 3 nm of rock salt and 4 nm spinel‐like phases. At a high‐temperature cycling 60 °C, the LiDFBP‐based electrolyte contained Li‐rich cells displayed 90% capacity retention at the end of 100 cycles. Likely, the structural stability of LiDFBP additive was anticipated due to the formation of stable SEI, which was due to the polymer‐like species through the reaction of phosphorous radical of LiDFBP with the EC molecules.^[^ ^266^ ^]^ The effect of tributyl borate (TBB) on the interfacial stability of LiNi_0.5_Mn_1.5_O_4_ cathode at 55 °C was studied by Huang et al. Modified electrolyte containing 0.5 vol.% of TBB in LiPF_6_ in EC:EMC:DMC enabled the Li ǀǀ LiNi_0.5_Mn_1.5_O_4_ cells to maintain a discharge capacity of 99.4 mAh g^−1^ with 83.3% capacity retention at the end of 50^th^ cycles between the voltage range 3.5 to 5.0 V under 55 °C. The SEI derived in the presence of TBB additive suppressed the oxidative decomposition of carbonates and the combination of TBB and PF_6_^−^ effectively inhibited the hydrolysis of LiPF_6_ and limited the generation of HF.^[^ ^267^ ^]^

The high temperature cyclability of LiMn_2_O_4_ based batteries was improved using a single electrolyte lithium salt Li(FSO_2_)(C_4_F_9_SO_2_)N (LiFNFSI). Electrochemical testing of LiMn_2_O_4_ cells wetted using LiFNFSI in EC/DMC electrolyte at 60 °C exhibited a prolonged operation for about 400 times than the conventional cells that employed LiPF_6_ in EC/DMC electrolyte solution. It was concluded that the LiFNFSI salt effectively suppressed the dissolution of Mn ions from the spinel LiMn_2_O_4_ cathode and also developed a Li‐ion conducting SEI films at a high temperature.^[^ ^268^ ^]^ Cyanate‐based functional additive is effective toward improving the high‐temperature operation of NMC cathodes, as they develop a stable and denser interphase with low resistance. Hexamethylene diisocyante (HDI) about 0.1% in combination with LiPF_6_ in EC:EMC electrolyte developed a dense interphase with minimal interfacial resistance. The graphite ǀǀ NMC622 cells presented an improved cyclability and a capacity retention of 82.6% at the end of 600 cycles in room temperature, and at 45 °C exhibited a capacity retention of 96.5%.^[^ ^269^ ^]^ Cyclopentyl isocyanate (CPI) based carbonate electrolyte improved the interfacial stability of Ni‐rich LiNi_0.83_Co_0.12_Mn_0.05_O_2_ cathodes. The LiPF_6_ in EC:EMC (3:7) electrolyte containing about 2 wt.% CPI improved the electrochemical performance of LiNi_0.83_Co_0.12_Mn_0.05_O_2_ cathode demonstrating a high voltage stability up to 4.4 V with a capacity retention of 81.2% after 200 cycles at 0.1 C rate. Even at a high current rate of 1 C, a good capacity retention of 92.3% (Figure 22e) was attained at 60 °C, the Ni‐rich cathodes exposed a capacity retention of 84.7% after 200 cycles. It was noted that the existence of cyano– group involved in the polymerization of HF and H_2_O in the electrolyte to develop a polyimide‐like interphase which adds stability to electrode/electrolyte layer at high temperature.^[^ ^270^ ^]^

**Figure 23:**
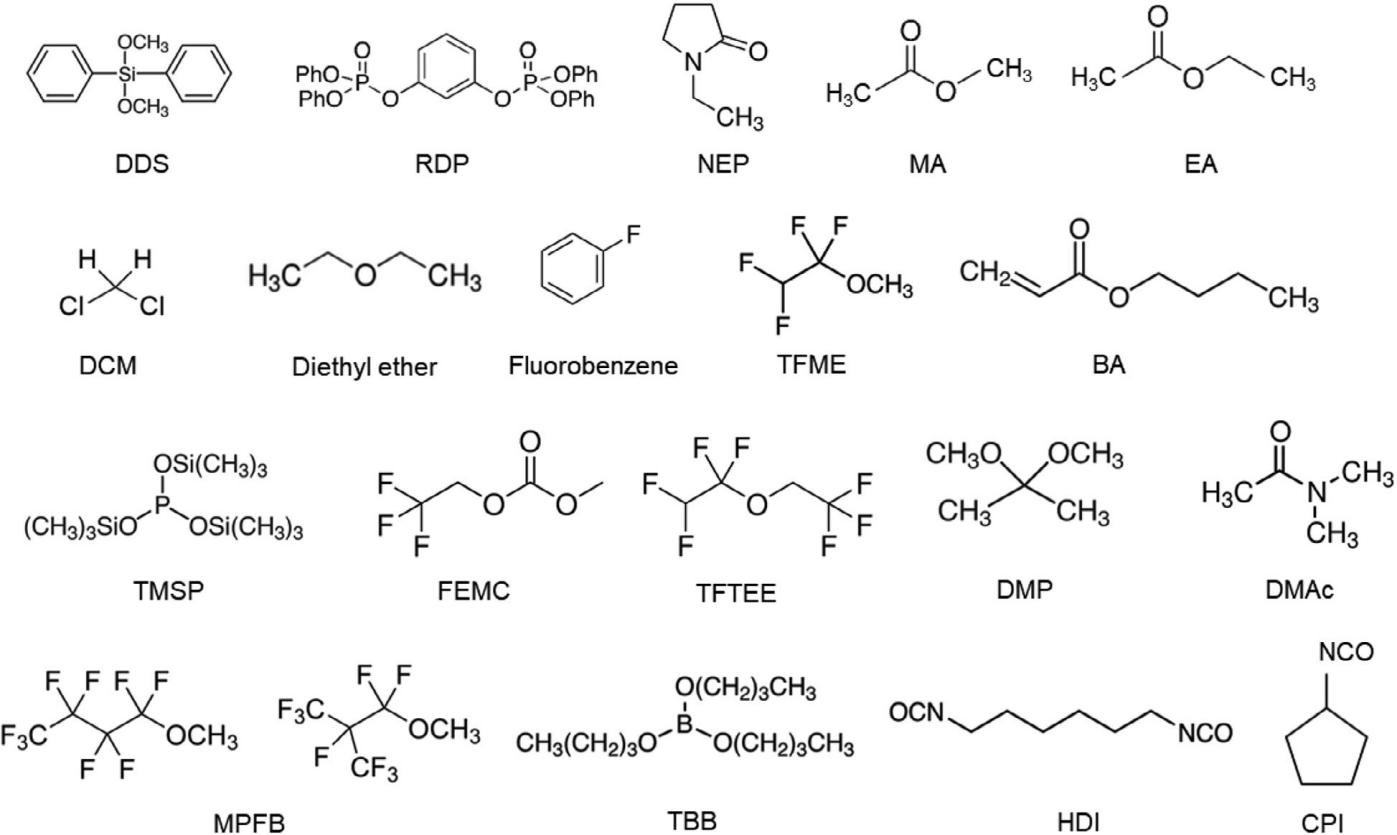
Chemical structures of functional additives for overcharge protection and wide‐temperature operation of Li metal and Li‐ion batteries.

All‐fluorinated electrolyte containing LiTFSI, FEC, and hexafluorobenzene (HFB) showed a wide‐temperature Li metal cyclability from –50 °C to 110 °C. Thermal studies revealed a comparably higher stability for the FEC+HFB electrolyte than the conventional EC+DMC electrolyte (Figure 22f). The Li stripping/plating behavior at 60 °C and at a high current density of 10 mA cm^−2^ remained stable for over 2000 h along with wide range temperature performance from 25 °C to 125 °C (Figure 22g). Half‐cells Li ǀǀ LiFePO_4_ showed good stability at–30 °C and at 60 °C delivering energy densities 287.77 Wh kg^−1^ and 327.21 Wh kg^−1^, respectively. It was inferred that the (FEC+HFB) additive played a critical role in improving the mechanical and chemical properties necessary for a long‐term electrochemical cycling. Considering the wide operation temperature limits of all fluorinated electrolytes, the LiF enriched interphase due to the decomposition of (FEC+HFB) afforded better mechanical properties to the interphase than the most commonly observed Li_2_CO_3_ and Li_2_O species. At a low temperature (Figure 22h), the Li‐ion transference certainly improved due to the coordination between TFSI^−^ and HFB which resulted in a weakened Li‐ion solvation and increased anion restriction. On the extreme temperature conditions (Figure 22h), the prevalence of LiF via (FEC+HFB) strengthened the interphase and inhibited the dissolution of Li_2_CO_3_ and Li_2_O interphase.^[^ ^271^ ^]^ **Figure** **23** depicts the chemical structure of functional additives that were employed for overcharge protection and wide‐temperature operation of Li‐based batteries.

High temperature operations of Li‐based batteries certainly induce accelerated reactivity at the interface leading to a thicker and resistive SEI. Suppression of autocatalytic electrolyte decomposition, stable film formation/altering surface chemistry, and scavenging targeted radicals via functional additives showed promising electrochemical performances. Additives‐based on boron like LiOB, LTMB, LiBMFMB, LiTFPFB and LiDFBP incorporated at minimal concentration < 2 wt.% demonstrated ability to form robust interfaces, mitigate hydrolysis of PF_6_^−^ ions, and inhibited O_2_ evolution and acid attack. Phosphor and cyanate‐based additives were efficient in developing mechanically robust interphase, especially for LiNi_0.5_Mn_1.5_O_4_ and Ni‐rich cathodes at high temperature cycling. A notable statement arises whether the operation of batteries at high temperature is necessary, however, the discussion may be satisfied not only due to any changes in the external temperature but also the internal thermal changes due to fast‐charging aspects.

From the perspective of practicability, improvements attained at low and high temperature performance of Li‐based batteries containing functional additive‐based electrolytes are adaptable for specific stationary batteries operated at a certain temperature, like exploration device at sub‐zero geographic regions or high temperature in space crafts. In certain cases, geographical locations might impose extreme and wide temperature variation limiting the operability of portable devices. However, a wide temperature operation from – to + (°C) require the design and development of more robust additives and electrolyte combination that remain stable and does the function without any adverse reaction. Designing a solvent that ought to retain the properties of an electrolyte at a wide temperature range akin to the room temperature cycling would be highly regarded. Such electrolyte engineering requires critical understanding on the properties of solute, solvent, ion mobility, interface at wide temperature in relation to electrochemical performances.

### 4.5 Acid and Water Scavenging Additives

Water contamination is inevitable in Li‐ion/metal battery's electrolyte solutions, especially from the components' solvents and salt. The presence of water, even in traces, is detrimental to the operation and stable cycling of Li‐based batteries. It is likely that the interaction between the conducting salt LiPF_6_ and water initiates the decomposition reaction leading to several irreversible products. Certainly, the presence of water in a liquid electrolyte undergoes reduction reaction to generate acid, which accumulates over a continued electrochemical cycling and corrodes the electrode materials and components. The mechanism of effect of water on LiPF_6_ follows,^[^ ^272^ ^]^

(2) $$PF_{5}+H_{2}O\to \mathrm{PO}F_{3}+2\mathrm{HF}$$

(3) $$2\mathrm{HF}+2\mathrm{Li}\to 2\mathrm{LiF}+H_{2}$$

(4) $$H_{2}O+\mathrm{LiP}F_{6}\to \mathrm{PO}F_{3}+\mathrm{LiF}+2\mathrm{HF}$$

(5) $$\mathrm{PO}F_{3}+H_{2}O\to H_{2}\left[PO_{3}F\right]$$

(6) $$H_{2}\left[PO_{3}F\right]+\mathrm{HF}\to H\left[PO_{2}F_{2}\right]$$

(7) $$H_{2}O+H_{2}\left[PO_{3}F\right]\to H_{3}PO_{4}+\mathrm{HF}$$

As notable from the series of reactions, the generation of HF could corrode or damage the SEI film. Purification process and sacrificial additives are effective strategies to eliminate the traces of water in an electrolyte. Functional additives carry the advantage of being at the electrolyte environment; particularly the Lewis base additives as scavengers are employed to formulate an electrolyte.

Dimethyl acetamide as an additive (about 1%) in 1 M LiPF_6_ in EC:DMC:EC electrolyte inhibited the effect of water with the LiPF_6_ salt. Mechanistically, the additive DMAc acted precisely by capturing the Lewis acidic PF_5_ moiety to form a stable (DMAc‐PF_5_) complex. Modified electrolyte tested in a MCMB ǀǀ LiNi_0.8_Co_0.2_O_2_ battery exhibited a minimal capacity loss of 0.38% per cycle against 0.76% for the blank electrolyte.^[^ ^273^ ^]^ Silane/silazane‐based compounds showed effectiveness toward the suppression of the acid generation by reacting with water molecules. Typically, the silyl groups (–SiR_3_, where R is an alkyl or aryl) can react with both acid and water contaminants forming stable byproducts: silyl groups as strong bases can react with acidic species like PF_5_ and HF through the donation of electron and also react with water to form silyl hydroxyl and more stable siloxanes. Therefore, the hydrolysis of electrolyte could be prevented, leading to stable SEI and CEI films. Hexamethyl disilazane (HMDS) was studied as a bifunctional additive to scavenge both H_2_O and HF species. Carbonate based electrolyte containing a small amount of HMDS additive about 500–1000 ppm suppressed the acid generation in Li_1.01_Mn_1.99_O_4_ cathode. Owing to the control of acid generation, the Mn dissolution was largely suppressed even upon exposure to about 80 °C.^[^ ^274^ ^]^ Further, the heptamethyl‐disilazane (HEMDS) additive was effective in preventing the acid generation in a carbonate electrolyte for the graphite ǀǀ LiMn_2_O_4_ cells. Even a small amount of about 0.1% HEMDS‐based carbonate electrolyte demonstrated good stability and effectively alleviated the HF content in the electrolyte that was generated post storage for about a week in room temperature. Modified HEMDS‐electrolyte showed performance improvement in graphite ǀǀ LiMn_2_O_4_ cells both room temperature and after storage at 60 °C. It was noted that the scavenging action of additive stems from the reaction between HEMDS and HF forming a stable compound.^[^ ^275^ ^]^ Another study by Wu et al. reported that the addition of about 2 vol.% of HEMDS additive to LiPF_6_ in EC: DMC: EMC (vol.%) electrolyte does not alter its ionic conductivity. The Li ǀǀ LiMn_2_O_4_ cells containing various concentrations of HEMDS‐based electrolyte showed capacity retention followed as 93% for 2 vol.% and 87.7% for 6 vol.% of additives. With a marginal amount of 3 vol.% HEMDS, the Li ǀǀ LiMn_2_O_4_ cells demonstrated a good thermal stability which was due to the preservation of spinel framework and crystallinity of the LiMn_2_O_4_ cathode through the additive.^[^ ^276^, ^277^ ^]^ Wu et al. reported that the combination of HEMDS and ethanolamine (MEA) additives formulated with LiPF_6_ in EC: DMC: EMC (vol.%) electrolyte preserved the surface of LiMn_2_O_4_ cathode. Owing to a strong passivation layer, the Mn dissolution from the LiMn_2_O_4_ particles was effectively suppressed. Understanding through the diffraction studies revealed that the crystal lattice of LiMn_2_O_4_ was maintained under the (HEMDS+MEA) additive‐based carbonate electrolyte, while a severe surface degradation of cathode was notable for the cells containing a blank electrolyte.^[^ ^278^ ^]^

**Figure 24:**
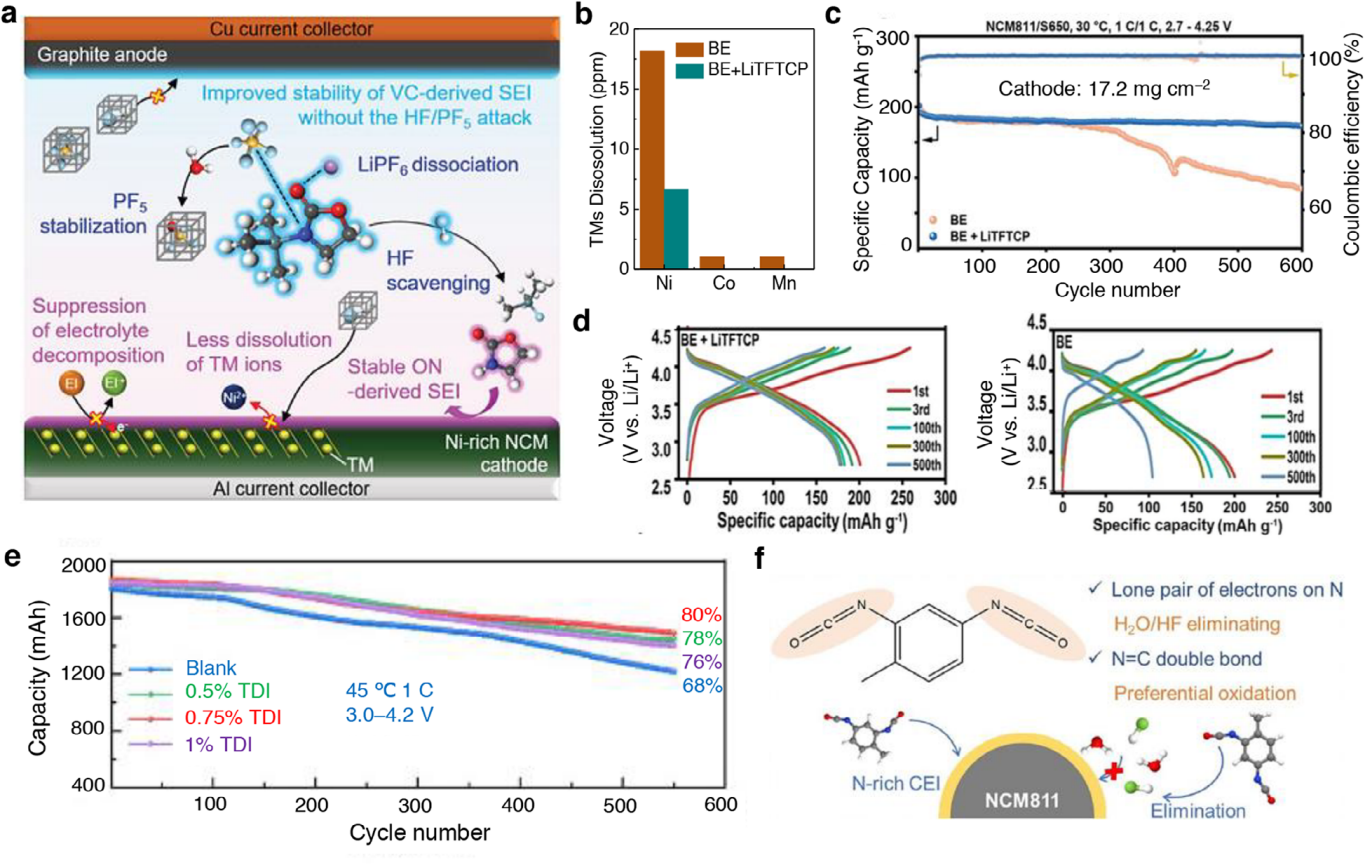
a) Schematics depicting the mechanism of 3‐(trimethylsilyl)‐2‐oxazolidinone (TMS‐ON) additive in constructing a stable interphase. Reproduced with permission.^[^ ^279^ ^]^ Copyright 2020, Wiley‐VCH. b) Comparison of metal dissolution in an electrolyte solution the presence of additive free LiPF_6_ and LiTFTCP additive. c,d) Capacity retention and corresponding voltage profiles of NMC811 cathodes obtained using blank LiPF_6_ and LiPF_6_+LiTFTCP electrolytes. Reproduced with permission.^[^ ^281^ ^]^ Copyright 2024, Wiley‐VCH. e) Capacity retention plot of NCM811 cathodes at a high temperature using LiPF_6_ EC:EMC and various concentrations of tolylene‐2,4‐diisocyanate (TDI)+LiPF_6_ EC:EMC electrolytes. f) Schematics portraying the benefits of TDI to improve the NMC cathode's electrochemical performance. Reproduced with permission.^[^ ^282^ ^]^ Copyright 2024, Elsevier.

Silyl‐based 3‐(trimethylsilyl)‐2‐oxazolidinone (TMS‐ON) additive showed multifunctional properties, which include film forming ability and acid scavenging properties as schematically portrayed in **Figure** **24**a. The addition of 0.5 wt.% TMS‐ON to 1 M LiPF_6_ in EC:EMC:DEC containing 1 wt.% VC electrolyte improved the performance of graphite ǀǀ LiNi_0.7_Co_0.15_Mn_0.15_O_2_ cells displaying a high‐capacity retention of 80.4% at 45 °C. Stabilization of LiPF_6_ electrolyte via TMS‐ON additive mitigated the acid generation and prompted improved performances of Ni‐rich cathode. The restricted hydrolysis of LiPF_6_ in the presence of TMS‐ON additive allowed PF_5_ to be stable, while the HF in the electrolyte was simultaneously captured following a strong interaction of fluoride anion and high affinity Si in N–Si moiety. Likely, as‐formed intermediate pentavalent silane subsequently formed a stable trimethylsilyl fluoride.^[^ ^279^ ^]^ Silane‐based 3‐aminopropyl)triethoxysilane (APTS) and 3‐glycidyloxypropyl)trimethoxysilane (GLYMO) were reported as an additive for high‐voltage LiNi_0.5_Mn_1.5_O_4_ cathode. The effect of both the individual additive APTS and GLYMO of each 0.5 wt.% blended with 1 M LiPF_6_ in EC:DMC:DEC forming an individual electrolyte was designed. The respective role of additives on the cyclability of LiNi_0.5_Mn_1.5_O_4_ were studied in half‐cells at room temperature and at high temperature. At a current rate of 1 C, both the modified APTS and GLYMO electrolytes contained cells showed an improved capacity retention of 92% and 72%, respectively after 350 cycles at room temperature. It was noted that the preferential oxidation of APTS and GLYMO resulted in the formation of a thin and stable CEI layer. The existence of Si─O groups in both the additive scavenged the acid and the ─NH_2_ groups of GLYMO assisted in constructing a stable CEI layer. In contrary to room temperature cycling, at 55 °C, the APTS electrolyte‐based cells demonstrated a high‐capacity retention of 72% against 15% for the GLYMO‐based cells. It was deduced that the build‐up of CEI layer's thickness over an increase in the cycles, which was about twice higher than that of APTS‐based cells worsens the performance degradation of LiNi_0.5_Mn_1.5_O_4_ cells containing GLYMO‐based electrolytes at high temperature.^[^ ^280^ ^]^ Cyanate‐based functional groups are effective film forming additives, which utilize their lone pair of electrons to downplay the required interfacial environment in the electrolyte solution. Hexamethylene diisocyanate (HDI) as an additive showed high temperature operation of graphite ǀǀ NMC622 cells through the development of a denser interphase, which remained stable even at elevated temperature.^[^ ^269^ ^]^ Cyano‐groups were introduced to lithium fluorinated phosphate to develop a lithium tetrafluoro(1,2‐dihydroxyethane‐1,1,2,2‐tetracarbonitrile) phosphate (LiTFTCP) additive. A significant suppression in the metal dissolution was observed with the LiTFTCP electrolyte than the blank electrolyte (Figure 24b). The LiPF_6_‐based carbonate electrolyte formulated with even a low concentration of 0.5 wt.% LiTFTCP additive extended the cycle life of SiO_x_–graphite ǀǀ NMC cells (Figure 24c,d) along with a good thermal stability. It was identified that the LiTFTCP additive altered the interfacial chemistry of both the anode and cathode. The effective suppression of HF in the electrolyte along with the existence of cyano groups imparted a good thermal stability to the CEI and also promoted the formation of LiF‐rich SEI layer. The multifunctional properties of LiTFTCP and its ability to alter the surface of both the electrodes benefit to safeguard the Li‐ion battery by limiting the electrode degradation, electrolyte decomposition, and thermally induced gas and heat‐release.^[^ ^281^ ^]^ Zeng et al. proposed tolylene‐2,4‐diisocyanate (TDI) as a bifunctional additive to curb acid and water contaminants in an electrolyte, especially at a high temperature. The TDI additive incorporated electrolyte employed graphite ǀǀ NMC811 cells remained stable even after 1000 cycles with a capacity retention of 94% at a current rate of 1C. At an elevated temperature of 45 °C, the NMC811 based cells were able to retain about 80% of the capacity (Figure 24e). The existence of lone pair of electrons of the isocyanate group in TDI inhibited the generation of HF and H_2_O (Figure 24f), which led to the formation of a thin, yet dense CEI interfacial film.^[^ ^282^ ^]^

**Figure 25:**
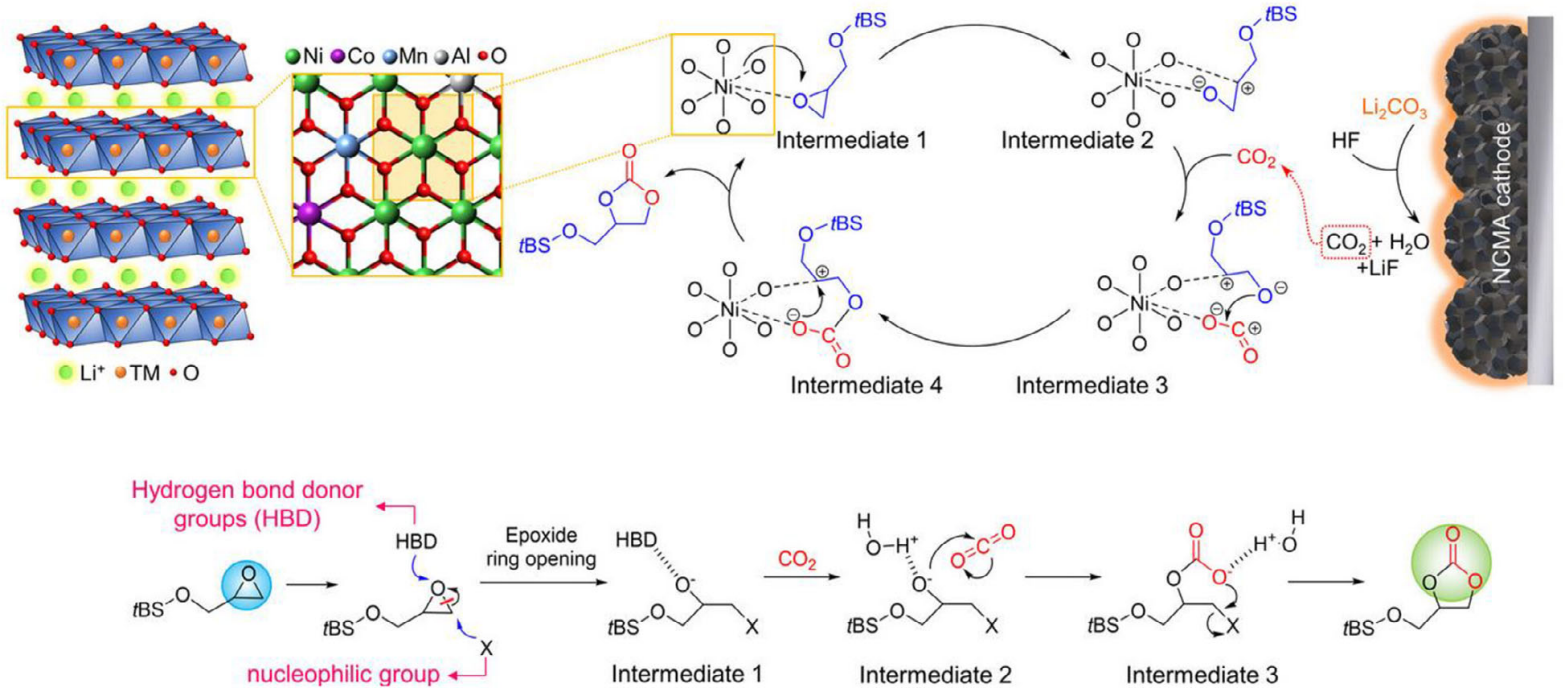
Plausible mechanism of CO_2_ generation and scavenging activity of epoxy group of *tert*‐butyldimethylsilyl glycidyl ether (*t*BS‐GE) additive. Reproduced with permission.^[^ ^283^ ^]^ Copyright 2023, American Chemical Society.

Ether‐based additive *tert*‐butyldimethylsilyl glycidyl ether (tBS‐GE) as an additive showed good interfacial properties in Ni‐rich cathode showing improved electrochemical performances. The interphase developed in the presence of tBS‐GE mitigated metal dissolution and showed effective toward scavenging acid and suppressed gas evolution. It was deduced that the cyclic carbonation at the NMCA cathode via tBS‐GE epoxide and CO_2_ produced Li_2_CO_3_ species. The intermediate generated by tBS‐GE additive reacted with CO_2_ forming alkyl‐carbonate‐like species. Later, the alkyl carbonate intermediates involved in an intermolecular ring closure with subsequent release of PF_5_ as a nucleophile forming cyclic carbonate (**Figure** **25**). Thus, it was likely that the epoxide group of tBS‐GE scavenged CO_2_ benefitting an improved cyclability. Electrolyte formulated with 0.1 wt.% tBS‐GE in 1.15 M LiPF_6_ in FEC:DMC (25:75, v/v) in graphite ǀǀ LiNi_0.9_Co_x_Mn_y_Al_z_O_2_ (NMCA, x+y+z = 0.1) cells showed about 85.5% capacity retention and high discharge capacity of 163 mAh g^−1^ after 500 cycles at 1 C rate.^[^ ^283^ ^]^ Li et al. reported vinyltrimethylsilane (VTMS) additive for high‐voltage operation and as acid scavenger for Ni‐rich cathodes. The decomposition of VTMS prior to the decomposition of native carbonate solvents enable to develop a stable interfacial film. Further, the stabilization of PF_5_ by overcoming the hydrolysis of LiFP_6_ effectively eliminated the HF from the electrolyte.^[^ ^284^ ^]^ Wang et al. reported 1,4‐phenylene diisocyanate (PPDI) as a multifunctional additive for graphite ǀǀ NMC622 cells. The PPDI additive developed a stable interphase on the graphite anode and effectively scavenged the HF and H_2_O species. Ex situ XRD studies demonstrated that the SEI developed in the presence of PPDI maintained the structural integrity of the graphite. The functional electrolyte formulated with PPDI enabled the graphite ǀǀ NMC622 cells to cycle over 200 cycles with a capacity retention of 81.3% at 45 °C.^[^ ^285^ ^]^

The solute and solvent are the primary source of contamination which along with the assembling line, such as dry room/box are the secondary source. Maximum protection from moisture or water is enabled, however, traces of contamination are inevitable. Silane‐based functional additives like HEMDS, TMS‐ON, APTS, and VTMS demonstrated scavenging properties for both acid and water impurities. Mechanistically, the strong basic character of silyl group reacted with acidic species like PF_5_ and HF forming a stable silyl‐based byproduct, while the interaction between silyl and water molecules formed stable siloxane species potentially stabilizing the interfacial films. Since the contaminants are traces in an electrolyte solution, early removal and pre‐determined additive concentration would be ideal.

### 4.6 Gas Evolution

**Figure 26:**
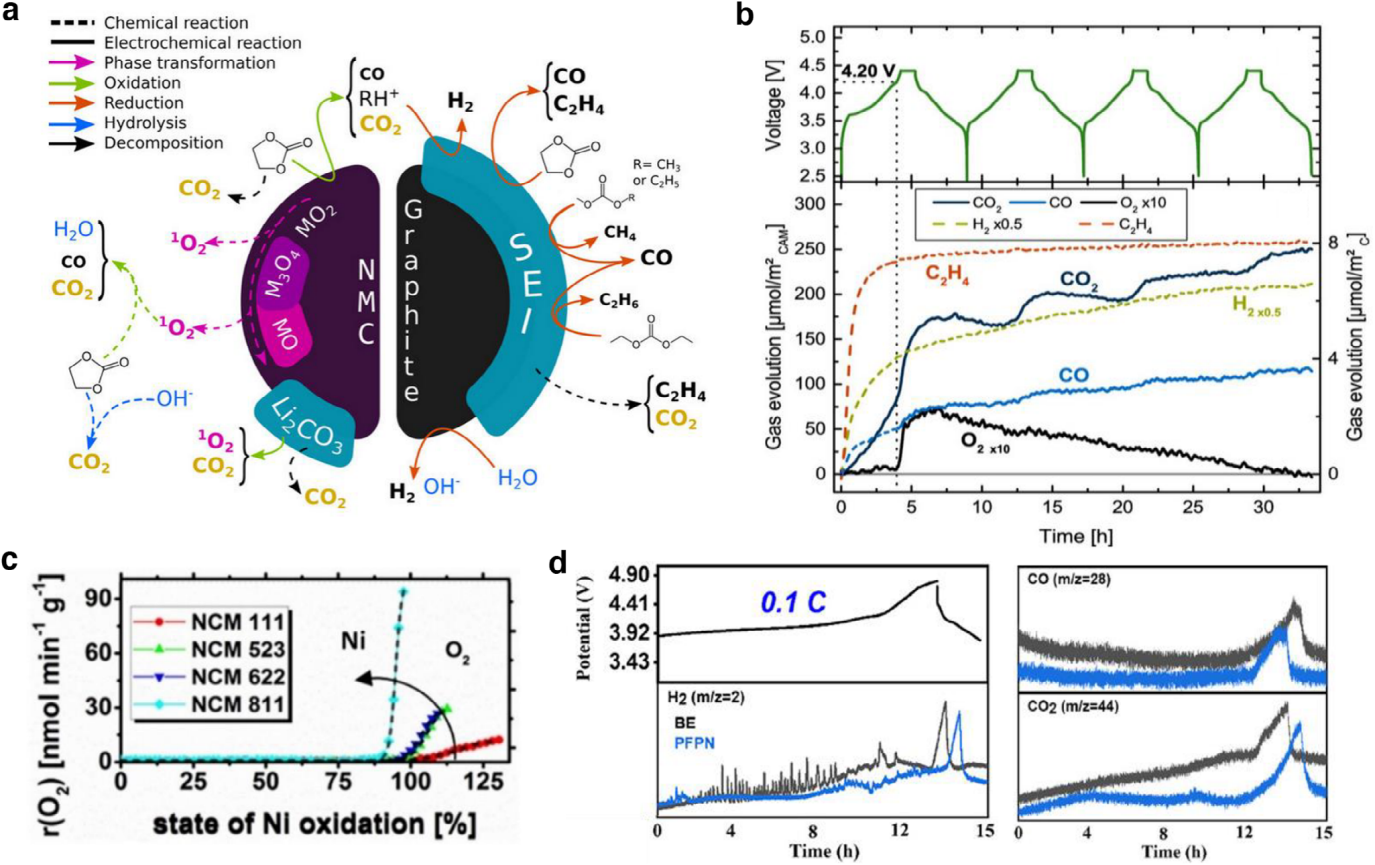
a) Pictorial representation of gas evolution due to several parasitic reactions during the operation of Li‐ion batteries, the gas evolution is based graphite ǀǀ NMC configuration. Reproduced with permission.^[^ ^288^ ^]^ Copyright 2023, The Electrochemical Society. b) The evolution of gas in graphite ǀǀ NMC cells containing LiPF_6_ in EC electrolyte at 0.2 C rate determined through an online electrochemical mass spectrometry (OEMS). Reproduced with permission.^[^ ^301^ ^]^ Copyright Authors 2017, The Electrochemical Society. c) Dependence of O_2_ evolution in various state of Ni oxidation in several stoichiometric NMC cathodes. Reproduced with permission.^[^ ^302^ ^]^ Copyright 2017, American Chemical Society. d) Charge‐discharge profile of NMC cathodes and the respective gas concentrations H_2_, CO, and CO_2_ over time compared with blank and PFPN‐containing electrolyte. Reproduced with permission.^[^ ^308^ ^]^ Copyright 2023, American Chemical Society.

The evolution of gaseous products in a Li‐ion battery can be identified under two conditions; i) formation cycle(s) and ii) consecutive charge/discharge cycles. Several source of gas evolution in Li‐based batteries includes the decomposition of electrolyte solvents on both the anode (reduction) and cathode (oxidation), and oxygen release (singlet oxygen or reactive oxygen, ^1^O_2_) from cathode material upon charge/discharge process.^[^ ^126^, ^286^, ^287^ ^]^ Detailed studies on the gassing mechanism in Li‐ion batteries were discussed by Salomez et al., and a pictorial representation of various sources of gas evolution in graphite ǀǀ NMC Li‐ion batteries is presented in **Figure** **26**a.^[^ ^288^ ^]^ During the formation cycle, the electrodes are electrochemically activated which is an essential process to construct a stable interface. It is widely demonstrated that the formation process offers positive influence on the battery performance in a longer run. Besides, there are several irreversible reactions due to electrolyte reduction/oxidation generating solid compounds and gases products. Solid particles are deposited on the surface of the electrodes and constitute a part of SEI and CEI films. Certainly, the interfacial morphology and structural properties have a lasting impact on the cycling performance.^[^ ^289^, ^290^ ^]^ Gaseous products such as CO, CO_2_, and H_2_ are part of electrolyte decomposition which are usually removed through degassing at the early stage.^[^ ^291^, ^292^ ^]^ Alongside, several hydrocarbons like CH_4_, C_2_H_4_, and C_2_H_6_ evolve during the cycling, while C_2_H_4_ is more prevalently observed in EC‐based electrolytes.^[^ ^293^ ^]^ For instance, formation process of graphite anode in a carbonate‐based electrolyte leads to the evolution of ethylene along with traces of hydrogen (due to water contamination) and carbon monoxide.^[^ ^294^, ^295^ ^]^ Studies by Laszczynski et al. reported that the evolution of gases in graphite ǀǀ NMC811cells increased with the cut‐off voltage due to stimulated parasitic reactions.^[^ ^296^ ^]^ Likely, the generated gases obstruct the surface of the electrode which might limit Li‐ion diffusion and could cause uneven current distribution. More serious is that the produced gases increase the pressure and cause changes in the volume of the batteries. Few reports that tracked the fate of water and gas evolution revealed a mismatch over the amount of moisture present in the electrolyte and the quantity of hydrogen generated during the electrochemical reaction.^[^ ^297^, ^298^ ^]^ In addition, there are mixed understanding on the influence of water on the cycling performance of Li‐ion batteries.^[^ ^299^, ^300^ ^]^ Apart, the gaseous CO produced during the electrochemical oxidation of carbonate molecules further reacts with the evolved lattice oxygen from the cathode particles to generate CO_2_ gas. Online electrochemical mass spectrometry (OEMS) studies revealed that the evolution of CO and CO_2_ gas at high voltage is due to the chemical reaction of lattice oxygen with the electrolyte (Figure 26b).^[^ ^301^ ^]^ Further, Streich et al. demonstrated that the cathode potential exceeding over 4.3 V gave rise to surface reconstruction producing substantial release of CO and CO_2_ gases. It was noted that the Ni content and the ratio of Ni/Co (Figure 26c) govern the onset, rate, and extent of the cathode particle surface reconstruction.^[^ ^302^ ^]^ It should be noted that the cycling conditions impact the ratio of CO_2_/CO gases produced and vary from 0.82 to 2.42 despite the similar electrochemical decomposition of electrolyte.^[^ ^303^ ^]^ A more comprehensive understanding on the gas evolution and its quantification in Li‐ion batteries was reported elsewhere.^[^ ^304^ ^]^ A summary of various sources of gas evolution during electrochemical cycling is provided in **Table** **3**.

**Table 3:** Summary of source of gas evolution during electrochemical cycling.

Gas	Source	Electrochemical condition	Potential (*vs*. Li/Li^+^)	Cell chemistry	
				Electrode	Electrolyte
H_2_	H_2_O	Reduction at anode	≤1.6 V	Graphite ǀǀ Li	LiTFSI in EC:EMC
	SEI damage	High anode potential	≥0.9 V	Graphite ǀǀ NMC	LiPF_6_ in EC:EMC
CO	EC decomposition	Reduction at anode	–	Graphite ǀǀ NCA	LiPF_6_ in EC:DEC
	DMC decomposition	Trans‐esterification	–	Graphite ǀǀ LCO	LiPF_6_ in DMC
CO_2_	SEI decomposition	Cycling at 55 °C	–	Li_2_CO_3_ ǀǀ LMC	LEDC+LiPF_6_ in DMC
	Electrolyte oxidation	Cathode	≈4.4 V	Li ǀǀ LCO	LiPF_6_ in EC:DEC
	Carbonate decomposition	PF_5_	–	–	LiPF_6_ in EC, EC, EMC, DMC
C_2_H_4_	SEI damage	High anode potential	≥ 0.9 V	Graphite ǀǀ LNMO	LiPF_6_ in EC:EMC
	EC decomposition	Reduction at anode	≤ 0.8 V	Graphite ǀǀ NMC	LiPF_6_ in EC:EMC
O_2_	Lattice oxygen–Cathode	High voltage operation	≈4.3 V	Li ǀǀ NMC	LiPF_6_ in EC:EMC

Vinylene carbonate, a well‐known film forming additive, is also effective toward suppressing the evolution of gas in a propylene carbonate‐based electrolyte.^[^ ^112^ ^]^ The additive VC works through the suppression of the reduction reactions related to the carbonate solvents, which eventually restricts the formation of alkyl carbonates and gaseous by‐products. Schwenke et al. investigated the composition of SEI and the evolution of CO_2_ in the presence of VC and FEC additives. It was known that the additives formulated as an individual electrolyte with 1 M LiPF_6_ in PC: EMC (3:7 wt.%) evolved CO_2_ attached to the polymeric structures. The subsequent step reduced the CO_2_ to inorganic carbonates like Li_2_CO_3_ and LiHCO_2_. Based on the observation, it was concluded that the presence of traces of CO_2_ in combination with H_2_O benefited the initial stage of SEI formation.^[^ ^305^ ^]^ The reduction of CO forming a stable lithium carbonates rather than its evolution as a gaseous product supported the report of Zhuang et al.^[^ ^306^ ^]^ In a different combination, addition of about 2% PS and VC formulated as individual electrolyte solutions significantly suppressed ethylene evolution. An early decomposition of VC and PS additives than the carbonates were estimated to suppress the ethylene gas. Further, the reduction products poly(VC) from VC and lithium alkylsulfonate (RSOLi) from PS additives inhibited the generation of ethylene, where a 60% reduction in ethylene evolution was noted for the combined (VC+PS) additive‐based electrolyte solution. However, the formation of gases during the consecutive cycling poses a threat to the operation of batteries.^[^ ^258^ ^]^

Cyclic sulfates as additives, including ethylene sulfate, trimethylene sulfate, and propylene sulfate, were investigated in graphite ǀǀ NMC111 cells. The electrolyte containing ethylene sulfate and trimethylene sulfate in combination with 2% VC demonstrated an improved C.E, while such tendency was not prominent for the propylene sulfate. Alongside, the electrolyte based on combination (TMS+VC) tested in graphite ǀǀ LiNi_0.33_Mn_0.33_Co_0.33_O_2_ cells does not show any evolution of gases, while the (DTD+VC) produced a significant amount of gases. It was noted that the VC acted as a gas reducing agent along with sulfate additives.^[^ ^157^ ^]^ Sulfone‐type additive propane sulfone showed positive impact on controlling the gas formation in Ni‐rich cathode materials at 60 °C. Electrochemical performance of LiNi_x_Mn_y_Co_z_O_2_ (Ni ≥ 0.6) cells in the presence of 2% PS additive containing carbonate electrolyte showed about 98.9% of initial capacity retention. It was deduced that the existence of sulfone‐based RSOSR and RSO_2_SR species through the electrochemical decomposition of propane sulfone (2% PS) significantly improved the surface properties of interphase. The reformed sulfone‐based interphase not only afforded an improved electrochemical performance but also enhanced the thermal stability of the cells.^[^ ^307^ ^]^ Ethoxy(pentafluoro)cyclotriphosphazene (PFPN) as a multifunctional additive improved the interfacial properties of SEI and CEI films. The amicable energy level of PFPN additive, low LUMO (−0.30 eV) and high HOMO (−8.23 eV) energy than the carbonate solvents (Figure 9a) enabled the PFPN to react ahead of the electrolyte solvents. Thus, the preferably developed SEI and CEI interfaces through the reduction and oxidation of PFPN additive benefited in improving the electrochemical performances of Li metal and NMC811 cathode. The electrolyte containing PFPN suppressed the structural changes of NMC811 at a high voltage certainly limiting the evolution of gases H_2_, CO, and CO_2_ (Figure 26d) through the development of a stable and uniform protective film. Lithium‐metal battery configured with NMC811 containing 7 vol.% PFPN in LiPF_6_‐EC:DMC:EMC electrolyte showed an improved capacity of 206.8 mAh g^−1^ and long‐term stability for over 200 cycles at a cut‐off voltage of 3.0–4.5 V.^[^ ^308^ ^]^

The evolution of gaseous products not only deteriorates the performance of rechargeable Li‐based batteries but also poses a serious risk to the safety factor. The root cause of gas evolution, the electrolyte decomposition, and structural collapse of cathode material were precisely controlled in the presence of the functional additives. Mechanistically, the functional additive worked via onset decomposition, conversion to carbonates, preserving the crystal structure of cathode material, and constructing a robust surface passivation layer. Sulfur and phosphorus containing additives control the evolution of gas by stabilizing the electrolytes, reducing the side reactions, and forming a protective interphase. Additives like propane sulfone and cyclic sulfates preferentially decompose and develop stable layers on the electrode surface, thereby preventing any side reactions and further decomposition. Although the additives worked on different strategies, obvious control in the gaseous products certainly contributed to better cyclability and stability of Li‐ion/Li‐metal batteries. However, a thorough understanding of the kinetics of solvent decomposition aligned with electrochemical cycling and the origin of gases must be undertaken.

### 4.7 Flame Retardant Additives

Carbonate based electrolyte solutions are highly volatile and are combustible in nature. The flammability of a material in terms of time could be classified as: non‐flammable t < 6 s g^−1^, flame retardant 6 < t < 20 s g^−1^, and flammable t > 20 s g^−1^.^[^ ^309^ ^]^ Phosphor and fluorine‐based compounds capable of generating P· and F· radicals are effective fire safety protection agents. These elements interrupt the combustion chain reactions of electrolyte decomposition products via scavenging the reactive free radicals like H· and OH· forming a thermally insulating barrier.^[^ ^310^ ^]^ It is worth mentioning that an increase in the concentration of additives raises the viscosity of electrolyte solution, which may result in a high impedance and poor capacity retention. More likely, the practical cost and environmental constraints of fluorinated additives are worth reconsidering. Thus, a trade‐off between the additive and electrochemical performances should be engineered and addressed promptly. The use of fire safety additives is expected to decompose on demand rather than the initial decomposition of solvent or film forming agents. This section details the progress achieved in mitigating the flame retardant properties in Li‐based batteries.

The effect of flame retardant ability of triphenyl phosphate (TTP) was studied in a carbonate LiPF_6_ in EC: EMC electrolyte. Modified TPP electrolyte showed a decrease in the ionic conductivity at −20 °C, while the cycling stability and capacity retention of MCMB anode do not change. Electrolytes containing a high concentration of TTP about 10 to 15% showed self‐extinguishing property, where the decomposition of TTP dictates the mechanistic action of flame retardant through either radical scavenging or thermally insulating barrier.^[^ ^311^ ^]^ Dufek et al. studied the trade‐off between the electrochemical performances and fire safety of Li‐ion batteries. Triphenyl phosphate and silicon‐containing phosphate as fire safety additives along with film forming additives like VC, VEC, and VEC were formulated with 1 M LiPF_6_ in EC: EMC (3:7) electrolyte. The addition of TPP and WC certainly improved the fire safety, and the electrolyte combination with 10% TPP and 5% FEC demonstrated good electrochemical performances for the MCMB ǀǀ Li cells and 10% TPP and 2% FEC for the NMC ǀǀ Li cells. It was concluded that the FEC additives provided improvements to the carbonate electrolytes containing TPP and Si‐containing phosphonate additives.^[^ ^312^ ^]^ Electrolyte formulated with gamma butyrolactone (GBL) and 1,1,2,2‐tetrafluoroethyl‐2,2,3,3‐tetrafluoropropyl ether (FEPE) with a lithium difluoro(oxalate)borate (LiODFB) salt improved the fire safety and cyclability of NMC532 based Li‐ion batteries. The 1 M LiODFB with GBL:FEPE (7:3) electrolyte formulation showed a comparable viscosity of about 4.13 mPa s and conductivity of 7.12 mS cm^−1^, while the blank 1 M LiPF_6_ in EC:EMC electrolyte stood at 4.00 mPa s and 10.80 mS cm^−1^, respectively. Additive‐based electrolyte showed a self‐quenching and better fire safety properties due to a high flash point of GBL ≈98 °C. The NMC532 full‐cells employing LiODFB in GBL:FEPE electrolyte exhibited an improved capacity retention of 82.2% after 500 cycles at 1 C rate, which was comparatively higher than the LiODFB in GBL and LiPF_6_ in EC:DMC electrolytes demonstrating capacity retention 71.2% and 77.6%, respectively. Even at wide operation temperatures, the LiODFB in GBL:FEPE electrolytes presented good stability and electrochemical performances in NMC532 full‐cells; at a low temperature of −40 °C and a high temperature of 60 °C, they exhibited a specific capacity of about 100 and ≈124 mAh g^−1^ against 17.1 and 121 mAh g^−1^ for the blank electrolytes, respectively.^[^ ^313^ ^]^ Siloxane‐based 1,3‐bis(cyanopropyl)tetramethyldisiloxane was reported as an electrolyte solvent that improved the electrochemical and thermal stability of Li–metal batteries. The highly concentrated electrolyte formulated as 3.5 M LiFSI in disiloxane possessing 0.5 wt.% LiDFBOB and 5 wt.% FEC as additives certainly improved the interfaces, SEI, and CEI films. As a result, the electrochemical performances of Li metal and NMC811 cathodes could attain an energy density of 614 Wh kg^−1^. In order to suppress the reduction of nitrile groups in disiloxane, a high concentration and coordination of Li^+^–FSI^−^ is required to prevent any adverse reaction of nitriles with the Li metal anode.^[^ ^314^ ^]^

**Figure 27:**
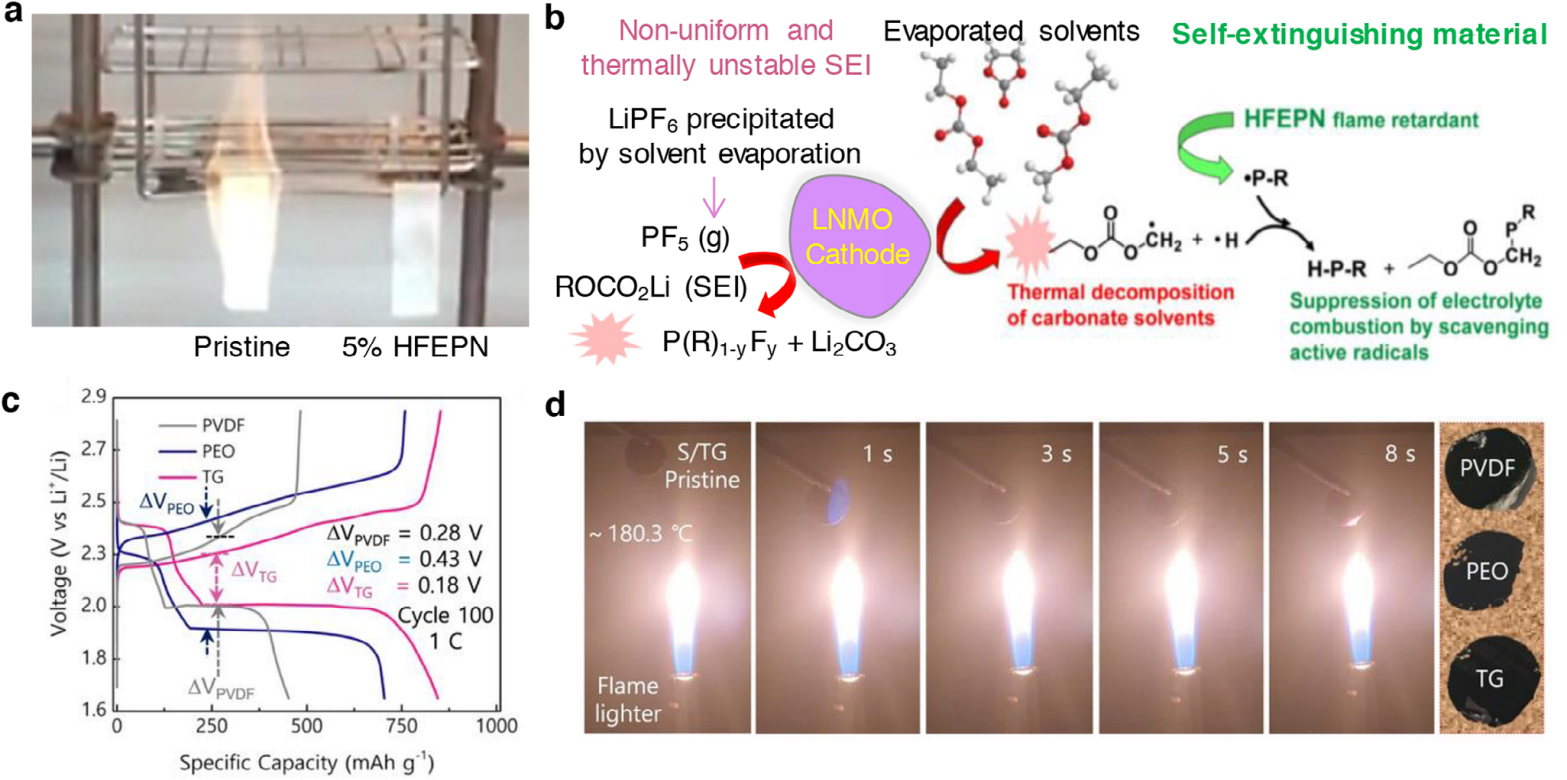
a) Flame test depicted for electrolytes (wetted over glass filter paper) without and with HFEPN additive. b) Schematics portraying the mode of thermal action of LiPF_6_ electrolyte and HFEPN flame retardant additive. Reproduced with permission.^[^ ^318^ ^]^ Copyright 2016, Wiley‐VCH. c) Discharge/charge profile of sulfur cathode fabricated using tragacanth (TG), polyvinylidine difluoride (PVDF), and poly (ethylene oxide) (PEO) as binders. d) The robustness of tragacanth‐based sulfur cathodes against flame followed by the recovered sulfur electrodes TG, PVDF, and PEO post flame tests. Reproduced with permission.^[^ ^323^ ^]^ Copyright 2022 The Authors, Springer Nature.

The fluorinated non‐flammable electrolytes possess high ionic conductivity and an extended electrochemical window. However, the reductive property of fluoroethylene carbonate on the anode triggers adverse effects on the battery performance. Prop‐1‐ene‐1,3‐sultone (PES) was proposed as an additive with FEC:DMC electrolyte to improve the interfacial properties and cycling stability of high‐capacity Si–G anode material. The electrolyte containing additive (ethoxy)pentafluorocyclotriphosphazene as a flame retardant improved the high voltage operation of Si–G ǀǀ LiCoO_2_ full‐cells to 4.45 V attaining over 80% capacity retention at a 1 C rate.^[^ ^315^ ^]^ The concentration of alkyl phosphates impacts the flame retardant property; however, the adverse effect often relates its incompatibility with the graphite anode. Partially substituting hydrogen with a fluorine atom at the terminal chain alkyl groups are effective in overcoming the performance barrier of Li‐ion cells. Murmann et al. investigated a rational relationship on the degree of fluorination, flame retardant, and electrochemical properties in the organophosphate additives. Three different phosphates, triethyl phosphate (TEP), tris(2,2‐difluoroethyl) phosphate (TDFEP), and tris(2,2,2‐trifluoroethyl) phosphate (TTFEP) were considered. Individually formulated additives TEP, TTFEP, and TDFEP with electrolyte LiPF_6_ in EC:DEC showed that an increase in the concentration of phosphates reduced the self‐extinguishing time. Still, the partially fluorinated TDFEP showed better flame suppression which contemplates that the fully fluorinated terminal methyl groups are necessary for restricting the flammability. The Li metal‐based cells constructed using MCMB and NMC electrodes showed better cycling performance containing TDFEP (30%) modified electrolyte.^[^ ^316^ ^]^ A fluorocyclophosphazene, (ethoxy)‐pentafluoro‐cyclo‐triphosphazene compound was reported by Xia et al. as a bifunctional additive, high‐voltage and fire safety performance. The carbonate electrolyte 1 M LiPF_6_‐EC: DMC (3: 7 vol.) formulated with 5% PFPN demonstrated good fire retardant properties and was compatible with the graphite anode and LiCoO_2_ cathode. Alongside, a notable capacity retention of 90% at a higher cut‐off voltage 4.5 V was shown by Li ǀǀ LiCoO_2_ battery containing modified PFPN electrolyte.^[^ ^317^ ^]^ Kim et al. reported a fluorinated hyper‐branched cyclotriphosphazene, i.e., hexakis‐(2,2,2‐trifluoro‐ethoxy)‐cyclo‐tri‐phosphazene (HFEPN) as a fire retardant additive. The electrolyte 1.15 M LiPF_6_–EC: EMC: DEC (3:5:2 vol.) containing 5% HFEPN self‐extinguished at about 2.9 s, while the blank electrolyte sustained combustion for over 6.5 s (**Figure** **27**a). In addition to the fire safety of HFEPN, the specific capacity of LiNi_0.4_Mn_1.6_O_4_ cells in the presence of HFEPN was improved to 114 mAh g^−1^ from 97 mAh g^−1^ at a 5 C rate for the blank electrolyte. The fire safety mechanism of HFEPN is schematically displayed in Figure 27b, where the decomposition of HFEPN additive produces phosphorus‐centric R–P• radical, which can further recombine with the hydrogen and carbon radicals generated via electrolyte decomposition to effectively suppress the flame.^[^ ^318^ ^]^

**Figure 28:**
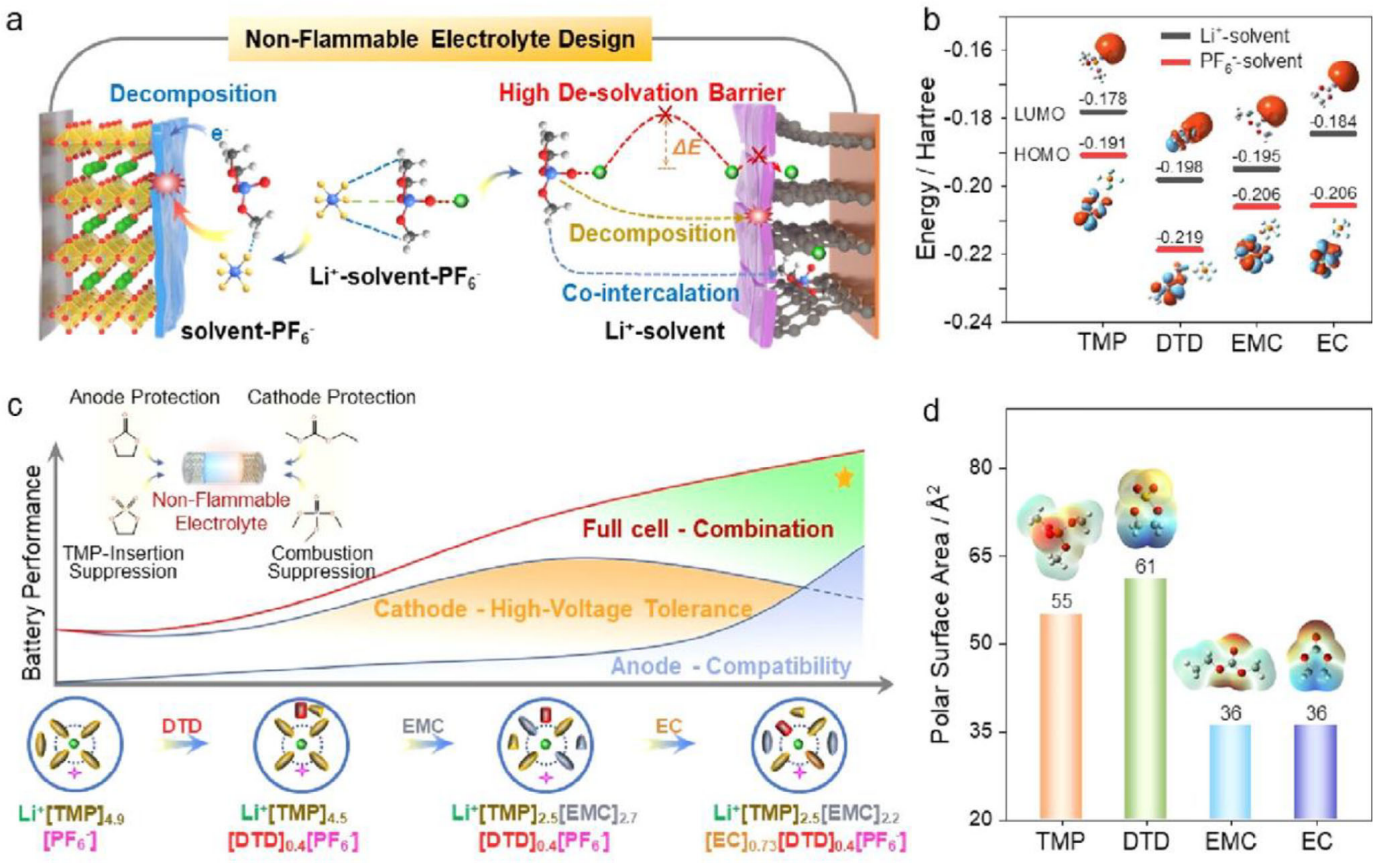
a) Pictorial representation of the effects caused by the LiPF_6_ electrolyte on the anode and cathode electrodes. b) LUMO energy of TMP and DTD solvents compared with the conventional EC and EMC solvents. c) Non‐flammable high‐voltage electrolyte design and the solvation structure model of TMP‐based electrolyte. d) Polar surface area of EC, EMC, TMP, and DTD solvents. Reproduced with permission.^[^ ^324^ ^]^ Copyright 2024, American Chemical Society.

**Figure 29:**
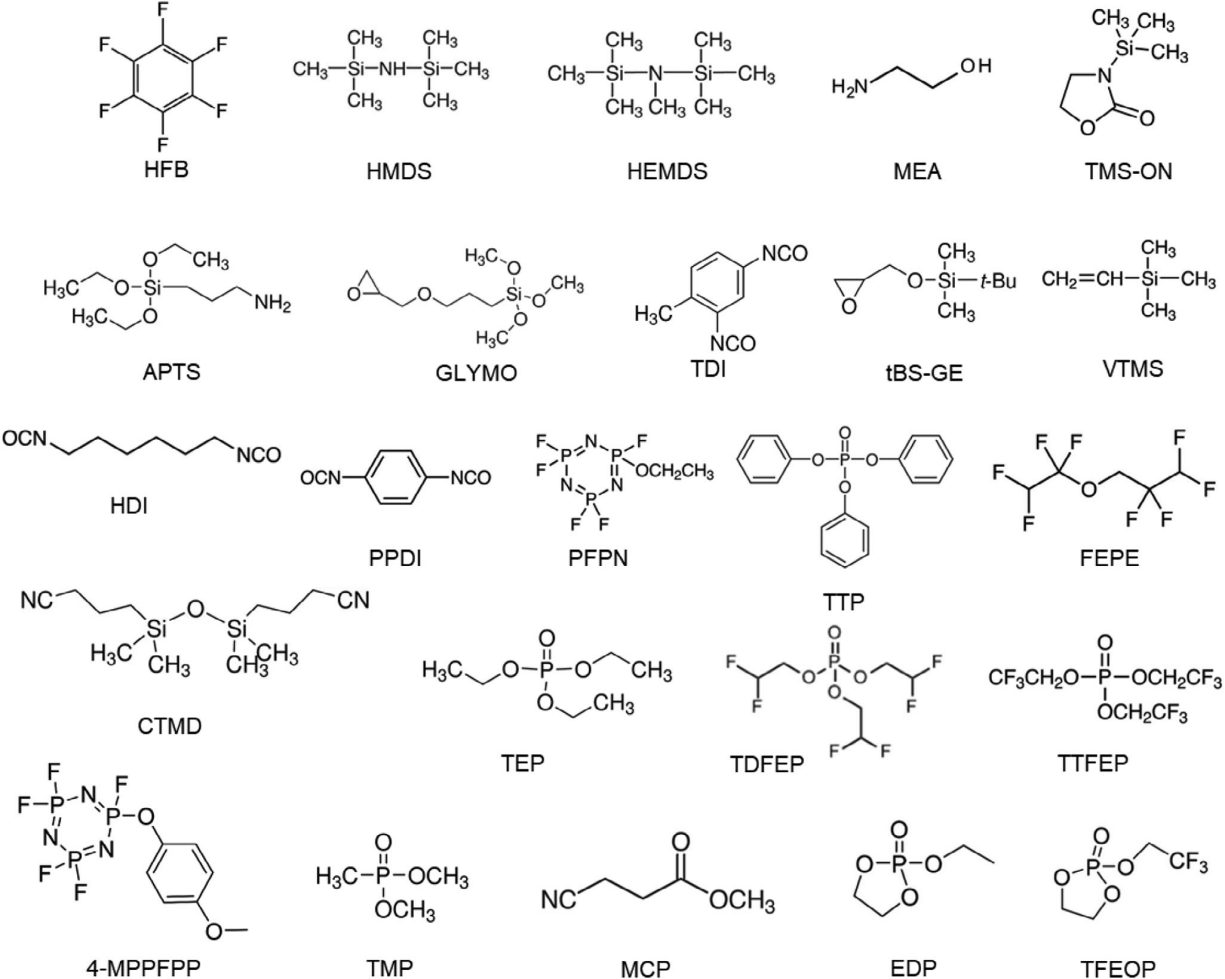
Chemical structures of functional additives adopted for acid and water scavenging and controlling the gas evolution and flame for Li‐based batteries.

The obvious role of cyclo‐triphosphazene and linear polyphosphazene as fire safety additives was substantial in both Li‐ion and Li–metal batteries. Meanwhile, the high molecular weight of cyclo‐triphosphazene raises the viscosity, which may affect the ion mobility leading to poor cell performance. For instance, a loading amount of 20% cyclo‐triphosphazene to 1 M LiPF_6_ in EC:DEC electrolyte almost doubled the viscosity and led to a decline in the capacity of about 25%.^[^ ^319^ ^]^ Dufek et al. developed a low molecular weight phosphazene compound about 450 g mol^−1^ in view of controlling the viscosity of electrolyte when used as an additive. The modified phosphazene containing a silyl moiety on the nitrogen benefits in reducing the viscosity and also limits the interaction of Li and N atoms. A high solubility of Li‐salts was shown by modified phosphazene and maintained a comparable viscosity below 8 cPa s even with a high loading of 35%.^[^ ^320^ ^]^ Another low molecular weight compound phosporimidic based additive, triethoxyphosphazene‐*N*‐phosphoryldiethylester (PNP), was developed by Wu et al. Addition of 10% PNP to 1 M LiPF_6_ in EC: DMC: DEC (1 vol.%) electrolyte displayed a decreased flammability. Lithium metal battery configured either with LiMn_2_O_4_ or LiFePO_4_ showed better cyclability, capacity retention, and C.E.^[^ ^321^ ^]^ Binary additives TMSP and PCS formulated electrolyte developed conductive and robust interfacial SEI and CEI films on anode and cathode, respectively. Further, PFPN as a fire safety additive in combination with TMSP and PCS as 1 wt.% TMSP + 1 wt.% PCS + 7 wt.% PFPN demonstrated a quick self‐extinguishing property as compared with the blank electrolyte. It was concluded that the self‐extinguishing behavior of PFPN was due to the generation of both F· and P· based radicals, which promptly suppressed the H· radicals to limit the combustion.^[^ ^168^ ^]^ Polymerized decomposition products forming an insulating layer is another effective strategy to prevent the carbonate electrolyte‐based Li‐ion batteries from thermal and voltage runaway. Huang et al. synthesized a phosphazene‐based (4‐methoxy)‐phenoxy pentafluorocyclotriphosphazene (4‐MPPFPP) compound and reported as an additive to prevent overcharge and fire protection. The base electrolyte showed a self‐extinguishing time of about 15.08 s cm^−1^, and upon addition of additive 4‐MPPFPP (8 vol.%) to the electrolyte restricted the burning time to 10.7 s cm^−1^. However, the electrochemical performances of Li metal with spinel LiMn_2_O_4_ cathode do not show any capacity improvement at 55 °C.^[^ ^322^ ^]^ Biopolymer based additive, tragacanth gum, was reported as an aqueous processable binder for Li metal‐based Li–S batteries. The sulfur cathodes fabricated using tragacanth gum as a binder demonstrated a higher sulfur redox‐ability and capacity retention than the conventional sulfur electrodes (Figure 27c). Interestingly, the biopolymer‐based sulfur cathodes displayed a good fire retardant capability. From the combustion tests as displayed in Figure 27d, it was found that the sulfur‐based biopolymer electrode remained stable against flame for over 8 s, while the polyvinylidine difluoride (PVDF) and poly(ethylene) oxide (PEO) based sulfur cathodes ignited immediately and burned. The flame retardant properties of tragacanth gum were owed to the construction of a stable thermopolymer upon exposure to a flame.^[^ ^323^ ^]^ Cheng et al. developed a non‐flammable electrolyte solvent trimethyl phosphate (TMP) based on a solvation structure‐mediated model as schematically represented in **Figure** **28**a. Fine‐tuned properties like the interaction of Li^+^–solvent–PF_6_^−^ and the de‐solvation and solvation structure benefited to control the flammability. The electrolyte containing an appropriate composition and ratio of solvent involving LiPF_6_ salt and EMC, EC, and TMP as solvents along with ethylene sulfate (DTD) additive showed good compatibility toward graphite anode and could withstand high‐voltage operation of LiCoO_2_ cathode. A rational comparison of energy levels (Figure 28b) demonstrated a high HOMO of −0.191 eV than the co‐solvents EC (−0.206 eV) and EMC (−0.206 eV). Owing to a well‐constructed solvation shell using combination of DTD, EMC, EC, and LiPF_6_ (Figure 28c), the electrolyte developed a robust SEI along with flame‐suppression ability. The self‐extinguishing property of nearly 0 s g^−1^ was shown by the TMP and DTD additive modified electrolyte as compared to 95.2 s g^−1^ for the conventional electrolyte. A rationally designed electrolyte using TMP and DTD as LiPF_6_ in [TMP]_2.5_[EMC]_2.2_[EC]_0.73_[DTD]_0.4_ employed in graphite ǀǀ LiCoO_2_ full cell exhibited a good cycling stability for over 200 cycles with a capacity retention of 83.2% at 1 C rate. Based on the solvation model and polar surface area (Figure 28d), it was deduced that the weakening of Li^+^–TMP interaction would allow the DTD moiety possessing high polar surface area to enter the first solvation layer, as the normal dissociation of LiPF_6_ salt in EC solvent can permit DTD molecules to actively associate in the solvation process. The developed solvation structure resulted in the EC molecules residing in the first solvation layer with utmost interaction between Li^+^ and EC forming an Li^+^–EC on the graphite surface, which significantly contributed to a more effective SEI structure.^[^ ^324^ ^]^ The chemical structure of the functional additives employed for scavenging acid and water contaminants and controlling the gas evolution and flame is presented in **Figure** **29**.

The functional additives containing elements phosphorus, sulfur, and fluorine showed great progress toward the suppression of flame. Typically, the free radicals P· and F· generated from the additive molecules effectively scavenged the H· and OH· radicals, being responsible for the combustion chain reaction. The resultant reaction between the radicals of additive and electrolyte components developed an insulating barrier that actively terminated any further reaction. Some of the additives like TMP and a mixture with DTD modulated the de‐solvation and solvation structure to restrict flammability. Though the flame retardant additives are effective to a certain extent, the safer option relies on designing a stable solvent and a more robust solution is the advancement of solid‐state electrolytes in the near future.

### 4.8 Anti‐Corrosive Additives

Safeguarding the properties of active components like anode and cathode materials are directly related to the improvement in the electrochemical performance like high capacity, capacity retention, cycling stability, and C.E. On the other side, the passive components, i.e., they do not actively participate in redox reactions but are of equal importance and highly required to achieve a stable operation of Li‐based batteries. The current collector is an essential component to conduct and transfer electrons from the anode and cathode materials to an external circuit. High‐purity foils of copper (Cu) and aluminium (Al) are predominant current collectors for the negative and positive electrode, respectively, due to their high electronic conductivity, processability, and cost‐effectiveness. The choice of current collectors, i.e., Cu for negative and Al for positive electrodes is based on their respective stability within the corresponding potential range. It is worth mentioning that the standard electrode potential for Al/Al^3+^ is about 1.38 V versus Li/Li^+^, which is far below the operating voltage of cathode materials (>3.0 V versus Li/Li^+^) for Li‐ion batteries. Interestingly, the naturally formed Al_2_O_3_ passivation layer on the surface of Al extends the oxidation potential to > 3.5 (versus Li/Li^+^) and certainly benefits in preventing the Al corrosion to a certain extent.^[^ ^325^, ^326^, ^327^ ^]^ However, upon continuous electrochemical cycling, the internal changes that occur with respect to the chemical environment like HF formation do not allow the Al current collector to sustain and remain stable. The corrosion of Al current collector can be represented as follows, where subscripts (ad.) and (dis.) represent adsorbed and dissolved species, respectively.^[^ ^327^ ^]^

(8) $$Al_{2}O_{3}\to 2A{l^{3+}}_{(\mathrm{ad}.)}+3/2O_{2}+6e^{-}$$

(9) $$\mathrm{Al}\to A{l^{3+}}_{(\mathrm{ad}.)}+3e^{-}$$

(10) $$A{l^{3+}}_{(\mathrm{ad}.)}+\mathrm{anion}/\mathrm{solvent}\to \mathrm{Al}-\mathrm{complexe}s_{(\mathrm{ad}.)}$$

(11) $$\mathrm{Al}-\mathrm{complexe}s_{(\mathrm{ad}.)}\to \mathrm{Al}-\mathrm{complexe}s_{(\mathrm{dis}.)}$$

**Figure 30:**
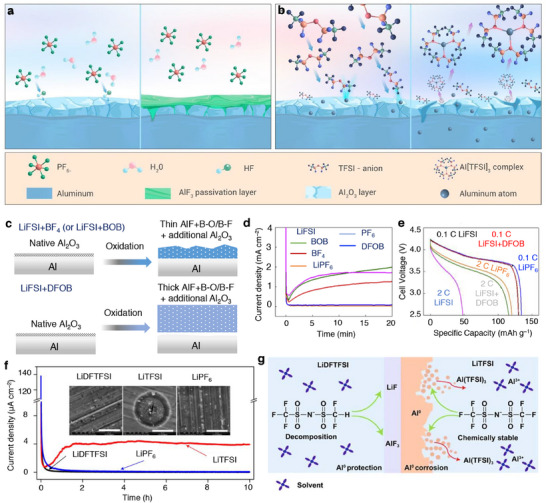
a,b) Corrosion mechanism portrayed for Li‐salts LiPF_6_ and LiTFSI based electrolytes, respectively. a) Fresh Al current collector followed by surface passivated Al exposed to LiPF_6_ electrolyte. b) Fresh Al and solution production in the presence of LiTFSI electrolyte. Reproduced with permission.^[^ ^327^ ^]^ Copyright 2023 The Authors, OAE Publisher. c) Schematic diagram of passive layer formation on Al current collector in the presence of LiFSI+BF_4_ or LiFSI+BOB compared with LiFSI+DFOB electrolytes. d) Chronoamperometry tests for the electrolytes formulated with LiTFSI salt in combination with BOB, BF_4_, PF_6_, LiPF_6_, and DFOB based electrolytes. e) Discharge profiles of graphite ǀǀ LiCoO_2_ cells with respective electrolytes. Reproduced with permission.^[^ ^338^ ^]^ Copyright 2015, Elsevier. f) Chronoamperometry test using LiDFTFSI, LiTFSI, and LiPF_6_ electrolytes and the respective SEM images of recovered Al electrodes. g) Schematic illustration showing the corrosion inhibition activity of LiDFTFSI electrolyte compared with LiTFSI electrolyte. Reproduced with permission.^[^ ^341^ ^]^ Copyright 2017, Springer Nature.

However, they are expected to remain stable over a longer cycle life in the presence of electrolyte solution. The corrosion of Al occurs at a relatively high potential especially with Li‐salts that lacks fluorine like LiClO_4_ or compounds possessing high compatibility with moisture like LiTFSI to limit the long‐term cycling.^[^ ^328^, ^329^ ^]^ For instance, the LiPF_6_ based electrolytes upon cycling generates an insoluble AlF_3_ and LiF species which effectively passivates the surface of Al preventing corrosion or anodic dissolution.^[^ ^330^ ^]^ Owed to the passivating nature of LiPF_6_ salt, they are used as additive or formulated as dual Li‐salt electrolyte to especially prevent Al corrosion.^[^ ^331^ ^]^ The schematic representation of corrosion mechanism of LiPF_6_ and LiTFSI‐based electrolytes in Li‐ion/Li–metal batteries are portrayed in **Figure** **30**a,b.^[^ ^332^ ^]^ Nevertheless, concerns regarding Al corrosion and its effect on the battery performances have not acquired significant interest. The anodic aluminum dissolution and/or corrosion in an electrolyte solution depends on three different factors: the electrolyte composition, surface structure of aluminum, and electrochemical operation conditions. This section discusses the additive's role in mitigating the Al corrosion and the type of mechanism involved in the process.

The compositional and morphological evolutions of the passivation layer developed over the Al surface was systematically studied by Yoon et al.^[^ ^333^ ^]^ Imide‐based salt, LiTFSI is the most considered conducting salt for Li–metal battery electrolytes, especially for Li‐S batteries. However, the LiTFSI‐based ether electrolytes promote the corrosion of Al current collector at a low potential > 3.8 V versus Li/Li^+^ as the C–F bonds of TFSI^−^ anions are stable enough to be oxidized generating a soluble Al(TFSI)_x_ species rather than AlF_3_ layer.^[^ ^334^ ^]^ Thus, the inability of imide salts to develop an effective passivation layer over the Al current collector leads to an anodic dissolution or corrosion, which seriously risks its adaptability in high‐voltage Li‐ion batteries.^[^ ^335^ ^]^ The adoption of electrolyte additives like HF, LiBOB, and LiDFOB are reported to facilitate the formation of a passivation layer over Al.^[^ ^86^, ^336^, ^337^ ^]^ A systematic study on the adoptability of several lithium borates as corrosion inhibitor in an LiFSI‐based electrolyte was reported by Park et al. The combination of LiFSI and DFOB electrolytes constructed thick layer of AlF and boron‐based compounds containing B–O and B–F groups (Figure 30c) over the Al current collector forbids the corrosion. Lithium borates that include LiBF_4_, LiBOB, and LiDFOB compared with LiPF_6_ showed inhibition of Al corrosion following the order, LiDFOB > LiBF_4_ ≈ LiPF_6_ > LiBOB (Figure 30d). The electrolyte (0.8 M LiFSI + 0.2 M LiDFOB) in EC:DEC demonstrated a comparable mitigation of anodic Al dissolution than that of the LiPF_6_‐EC:DEC electrolyte. However, the electrochemical performance of graphite ǀǀ LiCoO_2_ cells does not show much difference in the cycling or rate performances until subjected to 1.5 C rate (Figure 30e). Through ex situ XPS studies, it was revealed that the corrosion inhibition ability of LiDFOB arises from the formation of passivation layer on the Al surface due to the existence of Al–F, Al_2_O_3_, and B–O species.^[^ ^338^ ^]^

Meister et al. studied the effect of imide salts, LiTFSI and LiFTFSI containing electrolytes on the Al anodic dissolution. In particular, the electrolyte containing LiTFSI, the TFSI^−^ anions presented higher ability to suppress anodic aluminum dissolution than the LiFTFSI salt, FTFSI^−^ anions. Further, the study inferred that the role of salt and the type of anion, solvent, and operating temperature critically influenced the stability of Al at a higher potential.^[^ ^332^ ^]^ The choice of non‐fluorinated solvent, methyl 3‐cyanopropanoate (MCP) in combination with LiTFSI salt was studied by Brox et al. toward the suppression of Al dissolution. The cyanoester‐based electrolyte formulated as LiTFSI in MCP remained stable to the anodic dissolution in comparison to the LiTFSI in PC electrolytes. It was noted that the MCP solvent underwent reaction with the electrode and developed a passivation layer through insoluble Al(TFSI)_x_(O)_y_ species. Further, the solvents with a low dielectric constant could mitigate the Al dissolution in LiTFSI‐based electrolytes. Electrolyte containing MCP in LiTFSI displayed improved performance in a graphite ǀǀ NMC111 cell exhibiting a C.E of 94.4% against 86.7% for LiPF_6_ in MCP and 92.3% for LiPF_6_ in PC electrolyte‐based cells, respectively. Apart, the MCP solvent improved the thermal stability of LiTFSI electrolyte when compared to that of the LiPF_6_ in PC and LiPF_6_ in MCP electrolytes.^[^ ^339^ ^]^ Morita et al. elucidated the effect of LiPF_6_ in suppressing Al corrosion in dual salt system, whereas, Zheng et al. introduced a low concentration of 0.05 M LiPF_6_ additive to formulate LiTFSI–LiBOB dual‐salt/carbonate electrolyte for Li–metal batteries. The modified LiTFSI–LiBOB (0.6 and 0.4 M) with LiPF_6_ (0.05 M) in EC:EMC electrolyte not only improved the stability of Al current collector but also demonstrated good cyclability up to 500 cycles with a capacity retention of 97.1% in a Li ǀǀ NMC442 battery. It was concluded that the role of LiPF_6_ was crucial in stabilizing the Al foil and significantly influenced the nature of SEI on the Li anode. The decomposition of LiPF_6_ generating PF_5_, HF, and POF_3_ species acted as a catalyst promoting ring‐opening reaction of EC molecules. Thus, the SEI developed in the presence of LiPF_6_ are conductive and the resultant polycarbonate species covering the Li reduced the side reactions and disentanglement of dead Li from bulk Li metal anode.^[^ ^340^, ^341^ ^]^ The effect of adiponitrile and –CF_3_ substituted adiponitrile on the Al dissolution and solubility of Al(TFSI)_x_ species was investigated in imide‐based LiTFSI salt formulated as LiTFSI in ADN or LiTFSI in ADN–CF_3_ and PC co‐solvents. It was found that the extent of Al dissolution reduced by the substitution of –CF_3_ and the trend followed with the electrolyte solvents PC to ADN to ADN–CF_3_. It was noted that the addition of ADN and ADN–CF_3_ does not form any protection layer as a function of their decomposition, rather the oxidation stability, relative permittivity of solvents observed from low to high as ADN–CF_3_, ADN, and PC, ionic interactions and its transport properties had a positive impact on controlling the dissolution of Al.^[^ ^342^ ^]^

Qiao et al. developed and reported a non‐corrosive sulfonimide Li‐salt, lithium(difluoromethanesulfonyl)(trifluoromethanesulfonyl)imide (LiDFTFSI) which showed good compatibility with Al current collector at a high‐voltage > 4.2 V versus Li/Li^+^. The anodic stability studied through chronoamperometry (Figure 30f) revealed that both the LiDFTSI and LiPF_6_‐based electrolytes exhibited low current densities denoting the resistive nature of Al. Apart, the LiDFTFSI electrolyte possessed an ionic conductivity of 3.7 mS cm^−1^ and a high anodic stability up to 5.6 V (versus Li/Li^+^). Tracking the role of LiDFTDSI revealed that the chemical instability of Al(DFTFSI)_3_ in carbonate solvents readily decomposed to develop a stable passivation species like AlF_3_ and LiF species (Figure 30g). The Li–metal battery coupled with NMC111 cells in the presence of LiDFTFSI electrolyte exhibited similar capacities and comparable C.E.’s with that of the LiTFSI and LiPF_6_ electrolytes during the initial cycles at 1 C; 140 mAh g^−1^ and 85.6% for LiDFTFSI, 139 mAh g^−1^ and 83.8% for LiTFSI, and 138 mAh g^−1^ and 85.2% for LiPF_6_ containing cells. However, a good long‐term stability, capacity retention of 87% and C.E of 97% after 200 cycles was demonstrated by LiDFTFSI cells, meanwhile, approximately 41 mAh g^−1^ at 30 cycles and 25 mAh g^−1^ at 200 cycles were notable for LiTFSI and LiPF_6_ electrolyte, respectively.^[^ ^343^ ^]^

Aluminum foil remains a dominant choice of current collector for the positive electrode due to its chemical stability at higher potential. However, the changes in the chemical environment of electrolyte promote corrosion and anodic dissolution of Al current collector. Optimization of electrolytes containing additives LiTFSI, LiFSI, LiBOB, and LiDFOB enhanced the stability of Al foil by forming a passive layer. Limiting the dissolution or corrosion of Al provided robust support to the electrode resulting in improving capacity retention and cycle life of Li‐based batteries. Designing novel salts or solvents without compromising the fundamental properties of electrolyte solution would be ideal. Alongside, a pre‐coated Al foil that exhibits good compatibility with electrode and electrolyte without compromising the conductivity would be ideal for a long‐term stability under harsh chemical conditions.

## 5 Compatibility of Additive Mixtures: Synergistic Versus Agonistic Effects

Functional additives are tailored toward specific roles, where their compatibility among additive mixtures, battery components, or at multiple stages of battery operation gains critical importance while optimizing additives for reliable battery performances. This section exclusively discusses the issues related to additive mixtures in electrolyte solutions.

The interphase of graphite anode developed in the presence of additives VC and FEC improved the cycle‐life, but as an additive combination, VC and FEC exhibited converse ion migration properties, internal resistance, and cycle‐life in the graphite anode. Similar additive mixture benefited high‐capacity Si and Sn anodes, where the growth of a continuous SEI was restricted favoring an ionic interphase. Vinylene carbonate and its derivative DMVC afforded spatial flexibility to the Si anode's interphase reflecting an improved cycle‐life and acid scavenging properties via –OTMS moiety. Trimethylsilyllithium and VC+FEC blends synergistically constructed a polymeric SEI on Si anode, where the decomposition occurred systematically at 0.9 and 0.6 V leading to favorable performances. Sulfate‐based additives ethylene and trimethylene sulfate with VC conditioned the properties of dual interphase, while the integration of propylene sulfate does raise the cell impedance with adverse effect on C.E.

The cyclability of high‐voltage cathodes was enhanced through TMSP and PCS combination, while sultone and VC additives provided interfacial stability at an elevated temperature performance. The synergistic effect of sulfone and LiFSI mixtures preferentially constructed the anode and cathode interphases, respectively, whereas the LiF, due to LiFSI and complexation and polymerization of sulfone on CEI demonstrated extended cycle life. Boron‐based additives LiBF_4_, LiBOB, and LiDFOB as mixtures with LiFSI were effective inhibitors of Al corrosion, while LiBOB was also successful in stabilizing CEI. However, in an additive mixture, LiBOB is likely to induce anode‐electrolyte interfacial resistance. Nitrile additives SN, AN, and PN showed high voltage operation at the expense of cell resistance, while the incorporation of either sulfite/sulfate or phosphite/sulfite additives could improve the conductivity. Further, the anodic resistance built‐up in the presence of additive mixtures VC and sulfate was minimized using dosage of TMS and DTD additives.

Lithium nitrates are effective as additives for improving the compatibility between Li metal and carbonate electrolytes. However, the solubility of LiNO_3_ being a limiting factor in carbonate solvents, dissolution promoters like DITFA, HFT, CaF_2_, and Sn(OTf)_2_ improved the solubility and realized a good compatibility. Lithium metal anodes in the presence of LiTFSI or LiFSI contained ether‐based electrolytes benefited through a synergistic effect arising from dual additive LiNO_3_ and LiN_3_, while the addition of LiDFP to LiNO_3_ containing electrolytes improved the C.E. and long‐term cyclability. A rational combination of EC and FEC in carbonate electrolyte altered the wide temperature cyclability of Li metal. Film forming additives VC and LiNO_3_ synergistically enhanced the voltage limits of carbonate electrolyte up to 4.4 V.

Additive mixtures or additives with multifunctional properties showed enhanced interfacial properties like conductivity, stability, and strength influenced through synergistic cooperation. As a result, reliable cycling performance even at wide‐temperature, high‐voltage limit, and overcharge protection was notable. Furthermore, the additives ensured the safety of batteries by mitigating the contaminants, gas evolution, flammability, and aluminum corrosion. In the event of an agonistic effect, the cell resistance increased arbitrarily leading to poor battery performance with safety risks.

## 6 Engineering Electrolyte Additives: Guidance for Practical Relevance

The electrolytes, often termed as the “blood” of the battery, remain in mutual contact with both the electrodes, whereby offering a medium for the transport of ions. The role of electrolyte is critical to the electrode–electrolyte interface formation and significantly influences the battery performance, which includes capacity, cycle life, operating temperature, voltage, overcharge/discharge, gas evolution, and safety properties. To ensure an extended performance and safety of Li‐ion/Li–metal batteries, electrolytes contain a small dosage of additives to fulfil targeted roles such as stable interfaces, wide temperature limit, high‐voltage capability, limiting gas evolution, acid and water scavengers, and anti‐corrosion. Certainly, the electrolytes formulated using either mono or additive mixtures demonstrated an improvement in the battery performances, which were thoroughly discussed in relation to their mechanistic actions. However, a more prognostic approach relevant to the application of functional additives in the practical scenario would benefit the community across lab‐scale and industry. This section exclusively discusses the limitations of additives followed by guidance for engineering electrolyte additives focused on the practicality.

### 6.1 Guidance for Engineering Electrolyte Additives: Practical Relevance

Electrolyte containing additives demonstrated improvement in the performance at the lab scale, vectors like coin cell, Swagelok cells, and rarely pouch cells. Meanwhile, additives possess limitations including mass production, purity, compatibility, stability, cell resistance, cost, and toxicity. Considering the challenges and from the perspective of practical batteries, it is necessary to relook toward scaling up the entire value chain of additives from synthesis to its application. A detailed breakdown of the additive's challenges and improvements is as follows.

- *Mass production and purity*: Synthesizing additives on a large‐scale batch faces challenges like scalability, resources, manufacturing cost, and purity. Some of the additives like LiNO_3_ effective for Li metal protection have achieved market penetration; however, the vast collection of additives that have shown promising performance still remain at lab‐scale. Strategies to upscale affordable syntheses methods without compromising the purity of additives would benefit its infiltration in commercial electrolytes to build reliable batteries.
- *Compatibility*: The compatibility of additives can be classified into two categories, electrolyte formulation followed by the manufacturing process. Optimizing electrolyte formulation comprising solvent, co‐solvent, solute, and additives is a straightforward method. However, numerous additive mixtures create adverse reaction at any course, which must be systematically sorted. Further, integrating optimized additive mixtures to the existing manufacturing process is challenging. Thus, it must be ensured that the additives do not interfere with the performance of other battery components or the overall manufacturing process. Machine‐learning directed approaches would be helpful, however, one must be aware that the limited datasets and its reliability for designing novel additives impose substantial challenge.
- *Stability and shelf‐life*: Decomposition of additives during their action on‐demand and degradation over time due to storage may lead to adverse cell reaction. In addition, the byproducts produced after the targeted action of additives might react and trigger any new adverse reaction with any of the battery components. Thus, to obtain reliable battery performance, it is essential to ensure the long‐term stability of additives during the storage and use.
- *Cell resistance*: An increase in the conductivity of electrolyte and ionically conductive interphase has been observed in the presence of additives. Nonetheless, certain additives and their derivatives have an adverse impact on the cell resistance. Thus, the electrolyte formulations containing additives, especially their mixtures, are more vulnerable to cell impedance, which must be addressed while formulating electrolytes.
- *Cost*: The widespread adoption of several of the promising additives for practical batteries is restricted due to expensive and laborious processes. Designing cost‐effective syntheses methods and starting materials would make the additives more accessible and scalable.
- *Toxicity*: The environmental concerns due to the volatile, flammable, and hazardous properties of additives and battery components are alarming. Currently, much of the focus is on the recovery of high‐value metals from the cathode materials of spent Li‐ion batteries. However, the recovery of aged electrolyte gains equal importance, which is currently hampered due to recycling methods, high cost, and the recovery. A decisive transition toward the development of sustainable additives, electrolyte components, and synthesis methods is required.

## 7 Perspectives and Outlook

Electrolytes play an essential role in battery chemistries and are valued more in advancing fast‐charging and next‐generation high energy density batteries. The electrodes and electrolytes being the primary component that dictate the performance metrics of batteries, their inability in preserving physical–chemical properties due to thermodynamic and kinetic conditions promulgate several concerns related to capacity, cycle‐life, and safety of Li‐based batteries. Functional additives as a constituent in an electrolyte formulation effectively controlled several parasitic reactions pertaining to the operation of Li‐ion and Li–metal batteries resulting in an improved electrochemical–safety performances. The electrode‐electrolyte interphases developed in carbon/metal‐based anodes and cathodes in the presence of functional additive‐based electrolytes offered better conductivity, stability, and strength. The resultant interphases impart an enhanced cycling performance, cycle stability, and cycle‐life and are robust enough to even sustain the operations at wide‐temperature, high‐voltage limit, and overcharge protection. Alongside, a virtuous control of safety aspects involving contaminants in an electrolyte solution, gas evolution, flammability, and aluminum corrosion was accomplished in the presence of functional additives. Since the role of additives is specific and target‐oriented, the properties mitigated in a lab‐scale coin cell/Swagelok/pouch cell configuration resulted in an improved performance of Li‐ion and Li‐metal batteries. However, in a commercial battery, the complex reactions and the concerns classified as electrochemical and safety occur simultaneously and on‐demand, respectively, which require precise electrolyte formulations containing solute, solvent, and additives. Some of the issues related to electrode, electrolyte and additives, and device engineering that are worth investigating to further progress the way toward high energy density batteries are discussed below.

- Functional additives are mostly role‐specific, while a few additives offer multi‐functional characteristics. A rational combination of role‐specific and fine‐tuned additive composition would allow to expand the performance and operation limits of Li‐ion/Li–metal batteries. However, an adverse action owed to the additive mixture is probable; for instance, the flame retardant additives boosting the safety of a Li‐based battery do not support wide‐temperature operation. In such case, the additive combinations could compromise the performance as well as the safety of batteries, which must be considered while designing additive mixtures. Further, a thorough optimization and understanding of the functions of additives correlated to the electrochemical performance would be significant for further advancement.
- Wide‐temperature range of electrolyte solutions in the presence of functional additives was substantial. The developments associated with the interfacial layers SEI and CEI, inhibited Li dendrites, good ion conductivity, and suppressed parasitic/side reactions contributed to improved performances. Significant progress in the development of all‐temperature electrolytes requires understanding and addressing solutions to diverse conditions, stability of solute and solvent at extreme case, compatibility among electrode, electrolyte, and separator, structure and strength of electrode/electrolyte interface, solvation structure, and optimized ionic conductivity and fast Li‐ion kinetics.
- Thermal and fire‐safety of batteries upgraded in the presence of functional additives could last to a certain degree. Thus, the preliminary damage to the cells could be averted via additives, while an uncontrolled transition or propagation beyond the capability of additives poses an alarming situation. Several of the electrochemical parameters; cell voltage, charge/discharge conditions, state‐of‐charge (SOC), state‐of‐power (SOP), and state‐of‐health (SOH) are helpful for early diagnosis of physical factors like temperature and pressure. However, the propagation of such factors from the cell to the module and to pack must be counteracted at the earliest through precision sensing techniques, which is a robust and promising way to develop more reliable, safe, and sustainable batteries.
- The role of functional additives is phenomenal in raising the performance and operational safety of lab‐scale Li‐ion/Li–metal batteries. However, their practical constraints in terms of cost, processability, scalability, and environmental compatibility must be carefully revisited and addressed. Manufacturing costs related to additive synthesis, implementation, and formulating an electrolyte solution appear to be substantial. Although electrolytes contain only a nominal dosage of additives, yet an optimized and minimal concentration along with its incorporation during the mainstream electrolyte formulation might cooperatively balance the performance and cost. More alarming is that the environmental impacts of additives due to their toxicity and prompt disposal highlight the need for sustainable materials. Fluorinated compounds as additives in particular are harmful to the biological ecosystem, where a forcible move toward non‐fluorinated compounds would be an alternative. Meanwhile, proper handling, disposal, reclaim, and recycling of electrolyte and battery components would be effective to curb the waste and add value to sustainability.
- Scaling‐up the advanced instrumentation and capabilities would strengthen the understanding, like tracking the primary source for the gas evolution reaction during the electrochemical reactions on a large‐scale application is important. The knowledge acquired so far on the gas evolution and its sensing are limited to miniature devices at the lab‐scale and the conditions. To meet the demands of mega‐batteries adopted in large scale applications like transportation and grid storage, scaling up the concept and instrumentation facilities could help to build better batteries with reliable performance and efficiency.
- Advanced characterization techniques, especially in situ methods are much more relied upon to understand the real‐time information on the electrochemical system. The intricate and complex nature of SEI and its properties being dependent on the electrolyte and electrode, it is necessary to gain vast knowledge on the interfacial films. Several facts pertaining to interfacial films like porosity, strength, evolution of inorganic/organic layer and its chemical species are partially known and still a long way ahead. Alongside, the interface developed in the presence of an additive and their physical‐chemical‐mechanical properties, competitive role of electrolyte‐additive, and type of mechanism must be largely explored. Thus, more reliable advanced measurement systems could fetch handful of scientific knowledge to understand and advance the knowledge on interfacial films that are stable at normal and adverse voltage and temperature conditions.
- A generative move toward safe and high‐voltage batteries stems on the advancement of solid‐state electrolytes. The oxidation stability of solid‐state electrolytes being a material property, a fluoride‐based solid‐state electrolyte allows a high oxidation stability over 6 V versus Li/Li^+^. Limitations such as ionic conductivity, interfacial stability, and decomposition reactions of solid‐state electrolytes at a high voltage must be encountered.
- Multi‐interdisciplinary approach is critically essential to strengthen the core of Li‐ion/Li–metal batteries. Several disciplines but not limited to materials chemistry and engineering like thermodynamic modelling, mathematical models, thermal and energy management, electrical, mechanical, chemical, etc., would allow to mitigate and advance the safety and performance features. Such a multidisciplinary approach and contributions might help to build near‐to resilient batteries that are considered ideal.
- Machine‐learning directed approach to design electrolyte, solute, and additives and performance and life‐time prediction would be a targeted and data‐driven approach to largely support the mission on the advancement of high energy density batteries. A critical knowledge of the compositional ratio, solvation shell and structure, delicate properties of interface, compatibility with active and passive cell components, adverse reaction, and decomposition range pertaining to interface, dendrite‐related simulation, cell performance, aging, cycle‐life, etc., could immensely support the practicality of advanced batteries. A rational experiment–theoretical relation is necessary to foresee the battery advancements from chip to lab scale to industrial and commercial applications.

## Conflict of Interest

The authors declare no conflict of interest.
